# Ultra-deep sequencing reveals high prevalence and broad structural diversity of hepatitis B surface antigen mutations in a global population

**DOI:** 10.1371/journal.pone.0172101

**Published:** 2017-05-04

**Authors:** Mikael Gencay, Kirsten Hübner, Peter Gohl, Anja Seffner, Michael Weizenegger, Dionysios Neofytos, Richard Batrla, Andreas Woeste, Hyon-suk Kim, Gaston Westergaard, Christine Reinsch, Eva Brill, Pham Thi Thu Thuy, Bui Huu Hoang, Mark Sonderup, C. Wendy Spearman, Stephan Pabinger, Jérémie Gautier, Giuseppina Brancaccio, Massimo Fasano, Teresa Santantonio, Giovanni B. Gaeta, Markus Nauck, Wolfgang E. Kaminski

**Affiliations:** 1 Roche Diagnostics International Ltd, Rotkreuz, Switzerland; 2 Bioscientia Institute for Medical Diagnostics, Ingelheim, Germany; 3 Department of Molecular Genetics and Microbiology, MVZ Labor Dr. Limbach & Kollegen GbR, Heidelberg, Germany; 4 Roche Diagnostics GmbH, Penzberg, Germany; 5 Department of Laboratory Medicine, Yonsei University College of Medicine, Severance Hospital, Seoul, South Korea; 6 Roche Diagnostics GmbH, Mannheim, Germany; 7 Hepatology Department, Medic Medical Center, Ho Chi Minh City, Vietnam; 8 Gastroenterology Department, Ho Chi Minh City University Medical Center, Ho Chi Minh City, Vietnam; 9 Division of Hepatology and Department of Medicine, University of Cape Town and Groote Schuur Hospital, Cape Town, South Africa; 10 AIT Austrian Institute of Technology, Health and Environment Department, Molecular Diagnostics, Vienna, Austria; 11 Cerba Spécimen Services, Saint-Ouen l'Aumône, France; 12 Infectious Diseases and Viral Hepatitis Unit, Second University of Naples, Naples, Italy; 13 Infectious Diseases Unit, Department of Clinical and Experimental Medicine, University of Foggia, Foggia, Italy; Centre de Recherche en Cancerologie de Lyon, FRANCE

## Abstract

The diversity of the hepatitis B surface antigen (HBsAg) has a significant impact on the performance of diagnostic screening tests and the clinical outcome of hepatitis B infection. Neutralizing or diagnostic antibodies against the HBsAg are directed towards its highly conserved major hydrophilic region (MHR), in particular towards its “a” determinant subdomain. Here, we explored, on a global scale, the genetic diversity of the HBsAg MHR in a large, multi-ethnic cohort of randomly selected subjects with HBV infection from four continents. A total of 1553 HBsAg positive blood samples of subjects originating from 20 different countries across Africa, America, Asia and central Europe were characterized for amino acid variation in the MHR. Using highly sensitive ultra-deep sequencing, we found 72.8% of the successfully sequenced subjects (n = 1391) demonstrated amino acid sequence variation in the HBsAg MHR. This indicates that the global variation frequency in the HBsAg MHR is threefold higher than previously reported. The majority of the amino acid mutations were found in the HBV genotypes B (28.9%) and C (25.4%). Collectively, we identified 345 distinct amino acid mutations in the MHR. Among these, we report 62 previously unknown mutations, which extends the worldwide pool of currently known HBsAg MHR mutations by 22%. Importantly, topological analysis identified the “a” determinant upstream flanking region as the structurally most diverse subdomain of the HBsAg MHR. The highest prevalence of “a” determinant region mutations was observed in subjects from Asia, followed by the African, American and European cohorts, respectively. Finally, we found that more than half (59.3%) of all HBV subjects investigated carried multiple MHR mutations. Together, this worldwide ultra-deep sequencing based genotyping study reveals that the global prevalence and structural complexity of variation in the hepatitis B surface antigen have, to date, been significantly underappreciated.

## Introduction

Despite broad immunization programs in numerous countries since 1982, hepatitis B virus (HBV) remains widely prevalent with an estimated 240 million chronically infected subjects worldwide [1, 2]. Every year more than 780,000 deaths can be attributed to complications of chronic hepatitis B including cirrhosis and hepatocellular cancer.

HBV is differentiated into eight well characterized genotypes (A-H) [3, 4] and two recently discovered additional genotypes (I, J) [5, 6]. HBV genotypes are categorized by > 8% differences in their nucleotide sequence [7]. Numerous studies have demonstrated that different genotypes show different geographical distribution and are associated with disease progression, treatment outcome and prognosis. HBV genotype A is widely prevalent in America, Europe and sub-Saharan Africa [8]. Genotypes B and C are common in Asia and North America. Genotype D is found commonly in Africa, the Middle East and Europe. Genotype E is typically found in sub-Saharan Africa, whereas genotype F is prevalent in South America. Genotypes G and H are observed in Europe and Central America, respectively.

Detection of the surface antigen (HBsAg), the major HBV envelope protein, is pivotal for diagnosis of HBV infection and routinely used for testing of individuals with suspected HBV infection, therapeutic monitoring of infected subjects and screening of blood donors [9, 10]. Numerous variations in the HBV S gene give rise to a diversity of HBsAg mutations which are associated with immune escape, occult infection, and diagnostic escape [11, 12]. The average frequency of mutations within the HBsAg S gene has been found to be 11% in unselected North American populations and is increased to 47% in South Korean subjects with chronic HBV infection [13, 14].

Vaccine escape mutations of the HBsAg protein can result in viral infection that develops in a vaccinated subject. Neutralizing antibodies against the HBsAg protein are directed towards the highly conserved major hydrophilic region (MHR) of the surface protein (amino acids 99–170) that harbors the immunodominant “a” determinant region (amino acids 124–147). HBsAg variations that result in amino acid substitutions in the region 124–147 of the surface protein can induce conformational changes in the “a” determinant epitope so that it is not recognized by the neutralizing anti-HBs antibodies and thus escapes the control by vaccine-induced anti-HBs antibodies. This is particularly problematic in immunocompromised subjects as evidenced by recent work demonstrating that 75% of immunosuppressed subjects with HBV reactivation carried at least one HBsAg mutation, predominantly located in the MHR [15].

Another major problem posed by HBsAg mutations is their lack of detectability resulting in false negatives for HBsAg serological testing [16–18]. A recent meta-analysis encompassing 11,221 non-redundant HBV sequences indicated that a group of 8 HBsAg mutations associated with diagnostic failure (P120T, T126S, Q128H, G130N, S143L D144A and G145A/R) were prevalent at a frequency of 1% [19]. Moreover, a study in a pool of 4.4 million Dutch blood donations identified 23 HBsAg negative but HBV DNA positive individuals in different phases of HBV infection. The authors reported multiple S gene escape mutations in these subjects, in particular in the genotype D positive fraction [20].

The lack of HBsAg detection with serum HBV DNA levels compared to those usually detected in serologically evident (overt) HBV infection has been defined as ‘‘false” occult hepatitis B infection (OBI). This is typically due to mutations in the S gene (escape mutants), producing a modified HBsAg that is not recognized by some commercially available assays [21]. In contrast, in true OBI HBsAg is not detectable neither is serum HBV DNA or it is present in very low concentrations.

Given the global importance of HBV infection and the multifaceted impact of variations in the HBsAg protein on its diagnostics, clinical progression and treatment outcome, comprehensive knowledge of the diversity and frequency of these HBsAg mutations is essential. In the past two decades, evidence for the existence of HBsAg MHR mutations has largely been obtained from studies conducted in relatively small subject cohorts [22–29] with only few exceptions [13, 30]. In these studies, genotyping was routinely performed on the basis of conventional Sanger sequencing which is much less sensitive than recently developed ultra-deep sequencing methods [31]. In light of these significant limitations, the size of the worldwide pool of HBsAg MHR mutations is currently unclear and awaits systematic characterization.

In the present study, we explored, for the first time, the genetic diversity of the HBsAg MHR in a large, global cohort of unselected hepatitis B virus infected subjects using the highly sensitive ultra-deep sequencing method.

## Materials and methods

### Study population

A total of 1553 subjects originating from the continents of Africa, America, Asia and Europe, respectively, with confirmed HBV infection were recruited to the study. Serum or plasma samples were collected from the subjects between January 1 and December 31, 2014.

The combined study cohort included blood samples from randomly selected subjects with documented chronic HBV infection (HBs Ag positive for > 6 months, n = 562) and unselected HBs Ag positive subjects (n = 991) who were recruited from blood donation centers or vendors in Europe, South Africa, and the USA, respectively. The latter subcohort was included in an effort to include all known seven HBV genotypes (HBV A-G) in this global study.

The serum samples from the 562 subjects with documented chronic HBV infection were collected in Korea (n = 269) and Vietnam (n = 293), respectively. Written informed consent was obtained from each subject and the study protocol was approved by the Ethics Committees of the participating hospitals (Severance Hospital, Seoul, Korea; Medic Medical Center and Ho Chi Minh City University Medical Center, Ho Chi Minh City, Vietnam). Random samples from South African HBV-positive blood donors (n = 217) were obtained from the South African National Blood Services (Johannesburg, South Africa) following approval by the local Ethics Committee. For randomly selected subject samples originating from Africa, Asia, Europe, North America and South America, approvals by the respective institutional review boards and ethics Committees were available at the outset of the study. These samples (n = 569) were obtained from commercial vendors (SlieaGen, Austin, TX, USA; Discovery Life Sciences, Los Osos, CA, USA; Boca Biolistics, Coconut Creek, FL, USA). An additional set of de-identified samples from HBV infected subjects of Central European, African and Middle Eastern origin (n = 205), respectively, were provided by the Bioscientia Institute for Medical Diagnostics (Ingelheim, Germany). Ethics committee approval for the latter de-identified samples was not required.

### Ultra-deep sequencing of the HBsAg major hydrophilic region (MHR)

Ultra-deep sequencing was performed at two distinct sites, the Bioscientia laboratory, Ingelheim and the Limbach laboratory, Heidelberg, Germany. To systematically assess the diversity of the HBsAg MHR on the nucleotide level, PCR primers were designed that allowed amplification and ultra-deep sequencing of the complete MHR, defined as the segment spanning codons 99 to 170. This target region encompassed the first and the second loop of the immunodominant “a” determinant region. HBV DNA was extracted from 200 μl serum or plasma samples with a minimum viral load of 100 IU/mL using the MagNA Pure 96 instrument and the MagNA Pure 96 DNA and Viral NA Small Volume kit (Roche). The elution volume was 50 μl.

HBV DNA was amplified using a set of target-specific primers with fixed M13-tags (universal tails) and multiplex identifier (MID)-labeled primers in a two-step PCR universal tail approach (S1 Fig). In brief, in a first PCR round the region encompassing s-gene codons 83 to 227 was amplified using HBV-specific primers (HBV-fw: 5’TGGATGTGTCTGCGGCGTTTTATCAT3’, HBV rev: 5’ATDCKTTGACANACTTTCCAATCAA3’) with fixed M13-tags (M13-fw: 5’CCAGGGTTTTCCCAGTCA3’, M13-rev: 5’TCACACAGGAAACAGCTATGACC3’) resulting in a 731 bp PCR product that also covered the HBV POL gene (S2 Fig). In a second PCR round, the universal tags were targeted and extended using MID-labeled adaptor A and adaptor B primers. To allow assignment of each sequence to the pertinent subject, 94 unique MIDs were used as published previously (Technical Bulletin No. 005–2009, 454 Life Sciences, Roche). Bi-directional ultra-deep sequencing was performed for both strands on a GS Junior instrument using GS Junior+ chemistry according to the manufacturer’s instructions (454 Life Sciences, Roche). In each run an identical positive control sample bearing sets of known mutations was used.

### Validation of ultra-deep sequencing

To validate our 454 GS-FLX technique-based ultra-deep sequencing assay, we assessed the concordance between deep sequencing and conventional Sanger sequencing. For this, PCR amplification products of the HBsAg target region (731 bp) generated from a cohort of selected subject samples (n = 44) with known HBV HBsAg mutations (n = 333) were sequenced utilizing both techniques. To compensate for the significantly lower sensitivity of the Sanger method a common cut-off of ≥20% was used. All 333 mutations that were detected by Sanger sequencing were also detected by ultra-deep sequencing demonstrating 100% concordance between both sequencing methods (S1 Table).

### Analysis of HBV genotypes, sub-genotypes, MHR variants and mutations

SFF-files of each run were uploaded to a web interface (Austrian Institute of Technology, AIT, Vienna, Austria; Platomics GmbH, Vienna, Austria) together with annotation lists defining the MID sample correlation. The evaluation of HBV genotypes and sub-genotypes [32] was carried out by phylogenetic analysis [33].

In detail, we conducted variant calling as follows: The raw sequencing output was processed by an integrated variant calling pipeline. In the first step, the files were demultiplexed to assign the reads to the corresponding samples. Sequencing reads < 250bp were removed and all reads were pre-filtered and pre-processed using the 454 Long Amplicon data processing software (Roche). Each sequencing read was then checked for sufficient length (250bp) and primers and adapters were removed. In the next step, reads were clustered and assigned to the correct genotype and subgenotype, respectively. Genotypes with at least 100 reads (both forward and reverse strands) and a phred quality score greater than 20 on both strands were subjected to variant calling.

In each sample, an MHR variant was defined as a nucleotide sequence change in the S gene region (encoding amino acids 99 to 170) with an allele frequency >5% (in both sequencing directions) and at least 3 variant reads present on the forward as well as on the reverse strand. The percentage of a nucleotide variant was calculated as the number of variant reads in relation to the number of reads at that position. Based on these data a DNA consensus sequence was generated using IUPAC ambiguity codes for heterozygous SNPs. Finally, this consensus sequence was translated into a peptide sequence and compared to the genotype-specific reference sequences for analysis of amino acid exchanges.

An HBsAg MHR mutation was defined as a sequence variation that gives rise to an amino acid change or a premature chain termination relative to the genotype or subgenotype specific reference sequence. The genotype-specific sequences used as reference are listed in S2 Table.

### Statistical analyses

Statistical analyses were performed at the Austrian Institute of Technology (AIT, Vienna, Austria) utilizing a multi-step approach that included pre-processing, formatting, calculation and evaluation of all data. For this, Python scripts (version 2.7.6) and the scientific computing module NumPy were used. Graphical visualizations were created using the matplotlib package.

## Results

### Continental distribution of HBV genotypes

**Fig 1:**
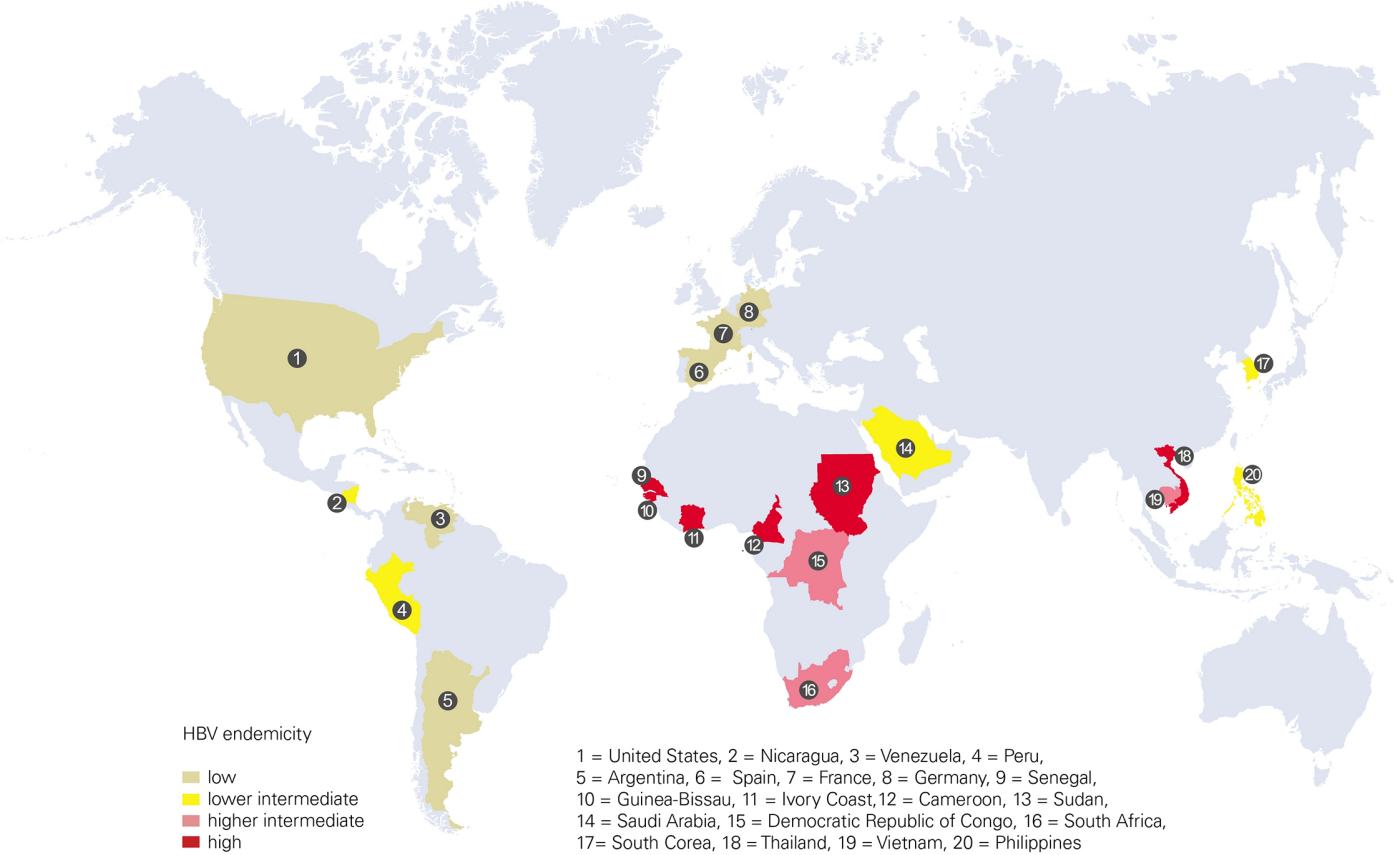
Global distribution of the patient cohorts included in this study. Numbers denote nationalities. HBV endemicites are color coded according to Schweitzer et al [2].

We performed ultra-deep sequencing of the HBV s-gene major hydrophilic region (MHR) in a randomly selected multi-ethnic global cohort of 1553 subjects with HBV infection. The subject samples were recruited from 20 distinct nationalities (S3 Table) localized in the continents of Africa (n = 435), Africa/Asia (n = 79), America (North America, n = 234; South America, n = 35), Asia (n = 653), and Europe (n = 72), respectively. The mean age of the subjects with documented chronic HBV infection (n = 562) was 39.7±13.1 (SD) years and, that of unselected HBsAg positive subjects (n = 991) was 39.9±12.5 years. For a total of 395 de-identified subject samples age information was unavailable. A specific effort was made to enroll populations that represented the complete spectrum of global HBV endemicity (low, intermediate, high) [2]. The two regions with highest HBV prevalence worldwide, sub-Saharan Africa and East Asia, in which 5–10% of the adult population are chronically infected [1], were represented by subject cohorts from Senegal, Guinea-Bissau, Ivory Coast, Cameroon, Sudan and Thailand, respectively (Fig 1).

A minimum bidirectional sequence coverage of ≥100 reads was obtained for each strand of the MHR encoding segment of the S gene in a total of 1391 subjects (male, n = 629; female, n = 430; unknown gender, n = 332). In this subcohort of the 1553 sequenced subjects, we detected seven HBV genotypes (A-G) (Table 1). The relative frequencies of the genotypes A-G in the combined global cohort were as follows: genotype A 22.9% (n = 318), genotype B 23.4% (n = 325), genotype C 28.8% (n = 401), genotype D 12.7% (n = 176), genotype E 11.6% (n = 162), genotype F 0.93% (n = 13) and genotype G 0.14% (n = 2), respectively. In only a small fraction of subjects (n = 6, 0.43%) co-infection with more than one HBV genotype was noted. Geographic mapping showed the distinct genotype frequency patterns that have previously been reported for various regions [32]. In the African cohort, the HBV genotypes A and E were by far the predominant ones. In contrast, in the Asian and North American cohorts the genotypes B and C were most prevalent. The European subject pool was dominated by the HBV genotypes A and D, and in the South American cohorts highest prevalence was noted for genotypes A and F. Moreover, the African/Asian cohort (Saudi Arabia) was dominated by a single *viz*. genotype D.

**Table 1:** Continental distribution of HBV genotypes. The predominant genotypes are highlighted for each (sub)continent.

Region	Number of patients	HBV genotypes*	A	B	C	D	E	F	G
Africa	396	400	197	13	3	45	142	-	-
Africa/Asia (Saudi Arabia)	80	80	2	1	1	68	8	-	-
America (North)	200	200	18	89	90	-	2	-	1
America (South)	35	35	20	1	-	-	1	13	-
Asia	565	566	51	211	303	-	-	-	1
Europe	114	115	30	10	4	62	9	-	-
Unknown	1	1	-	-	-	1	-	-	-
Total	1391	1397	318	325	401	176	162	13	2

* Note that in few patients more than one HBV genotype was detected.

### Sequence diversity in the HBsAg major hydrophilic region (MHR)

**Fig 2:**
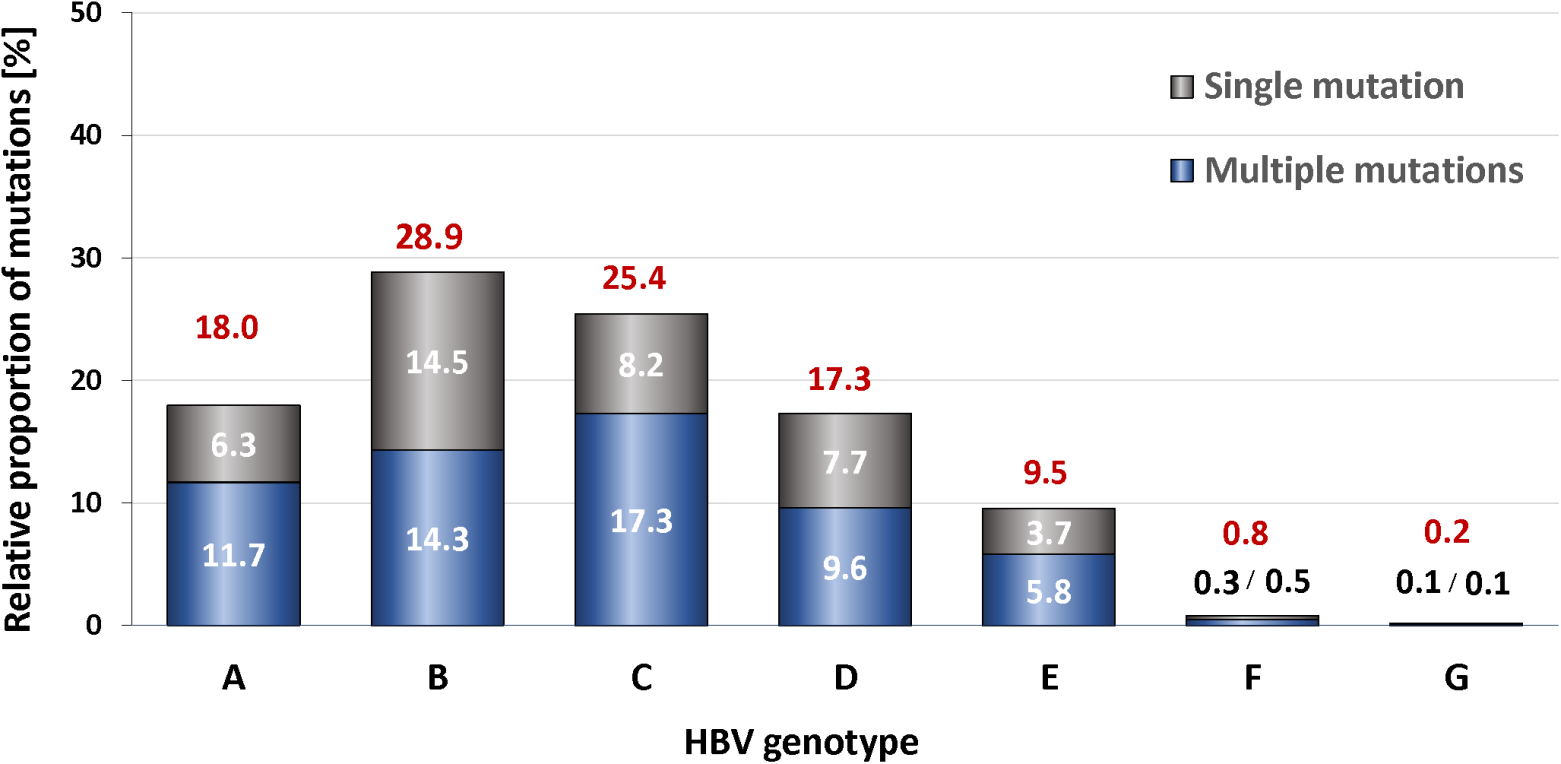
**Quantitative distribution of mutations of the HBsAg major hydrophilic region (MHR) among different HBV genotypes (A-G).** The numbers above the bars indicate the relative proportion of mutations in the total pool of mutations that were identified in this study (n = 345, 100%). Individual ratios of single mutations (gray) vs. multiple mutations (blue) are shown for each HBV genotype.

Sequence variation in the MHR (aa 99–170) is of considerable clinical and diagnostic relevance. In our global cohort (n = 1391), a total of 1013 (72.8%) subjects displayed mutations in the HBsAg MHR (S4 Table). A mutation was defined as a genetic variation that induces an amino acid change or a premature chain termination in the HBsAg MHR in at least one HBV genotype. No significant differences in the frequencies of the HBsAg MHR mutations were observed between various age groups, which ranged from 68–78%, or between males and females (S5 Table). The highest frequency of MHR mutations was seen in genotypes D (100%), G (100%), and B (90.5%), respectively, which were followed by genotypes C (64.6%), F (61.5%), E (59.9%), and A (57.5%) (S6 Table). Altogether, we identified 345 mutations in the combined four-continent subject cohort within at least one single genotype (S7 Table). Within the pool of 345 mutations the relative proportion of mutations was highest in the HBV genotypes B (28.9%) and C (25.4%), followed by genotypes A (18.0%), D (17.3%) and E (9.5%), respectively (Fig 2). Genotypes F (0.8%), and G (0.2%), exhibited the lowest percentage of MHR mutations. The latter percentages may not be statistically representative because of the small number of subjects carrying the genotypes F (n = 13) and G (n = 2).

Remarkably, more than half (59.3%) of all HBV subjects carried more than one MHR mutation. We noted that for all HBV genotypes the portion of subjects carrying multiple mutations was larger than that carrying only a single mutation, with the exception of genotype B which encompassed slightly more subjects with a single MHR mutation.

### Sequence diversity in the MHR “a” determinant region

**Fig 3:**
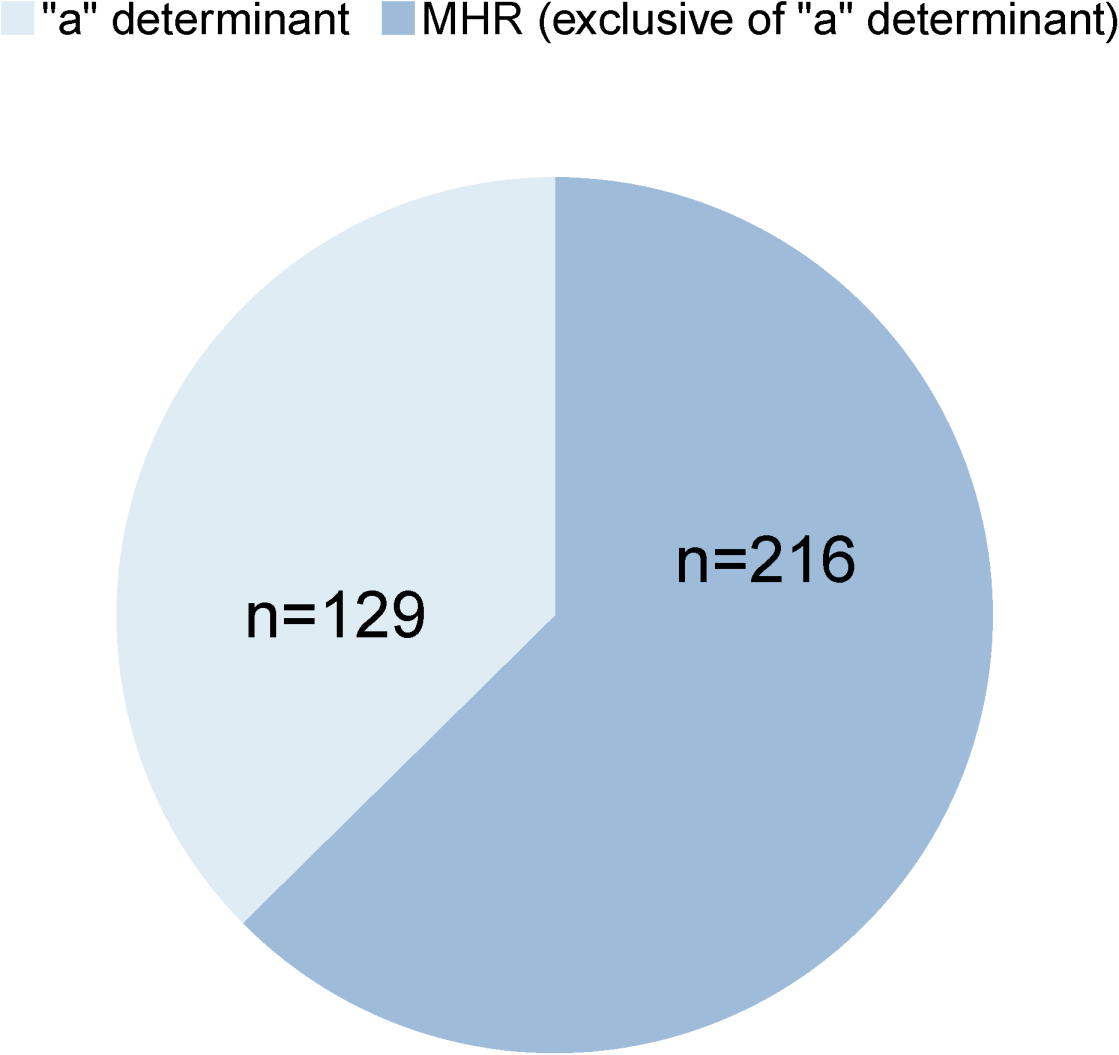
Quantitative distribution of the 345 mutations identified within the HBsAg MHR (aa 99–170). Shown are the fractions of mutations that localize to the immunodominant “a” determinant region (aa 124–147, n = 129) and those that lie in its flanking segments (n = 216).

**Fig 4:**
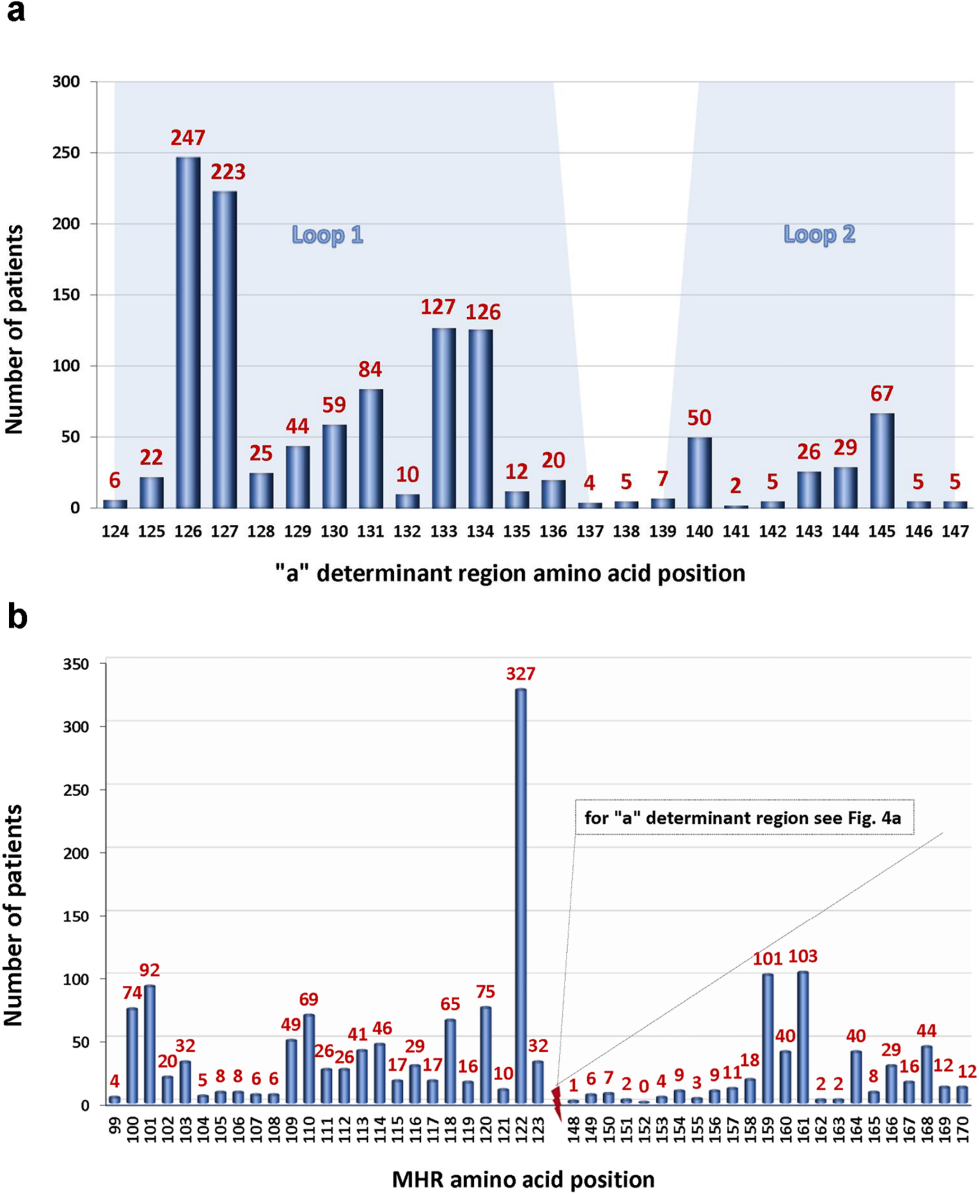
**a. Frequency and location of mutations in the HBsAg MHR “a” determinant region in HBV infected patients (n = 1391) from four continents (Africa, America, Asia, Europe).** The number of patients bearing a mutation is shown for each of the amino acids that constitute the "a" determinant (aa 124–147). Note highest variation at aa 126, aa 127, aa 133, and aa 134, respectively ("a" determinant loop 1). The blue shaded areas represent “a” determinant region loop 1 (aa124-137) and loop 2 (aa139-147). **b. MHR mutations outside of the “a” determinant region (aa99-123 and aa148-190).** Shown are the results for the same patients as in (a). Note the dominance of MHR mutations at aa122.

Genetic variations in the “a” determinant region of the MHR are of particular importance because it represents the immunodominant domain of the HBsAg. Ultra-deep sequencing revealed that the majority (62.6%, n = 216) of the identified 345 MHR mutations localize to the flanking segments of the “a” determinant region and only a subfraction of 129 (37.4%) mutations is located within the “a” determinant (Fig 3, S8 Table). In this domain, the highest number of mutations was observed in loop1 (aa124-137) at aa126, aa127, aa133, and aa134, respectively. “Hot spots” in loop 2 (aa139-147) of the “a” determinant region were identified at aa140 and aa145 (Fig 4A). The majority (62.6%, n = 216) of the identified MHR mutations were localized outside the “a” determinant domain in the regions aa99-123 and aa148-170, respectively (S7 Table). Highest prevalence of these amino acid substitutions was found at aa100, aa101, aa122, aa159, and aa161 (Fig 4B). Of note, the well characterized mutation hot spot at aa122 showed the previously documented dominance over all other sites of variation.

### Genotype distribution of “a” determinant mutations

**Fig 5:**
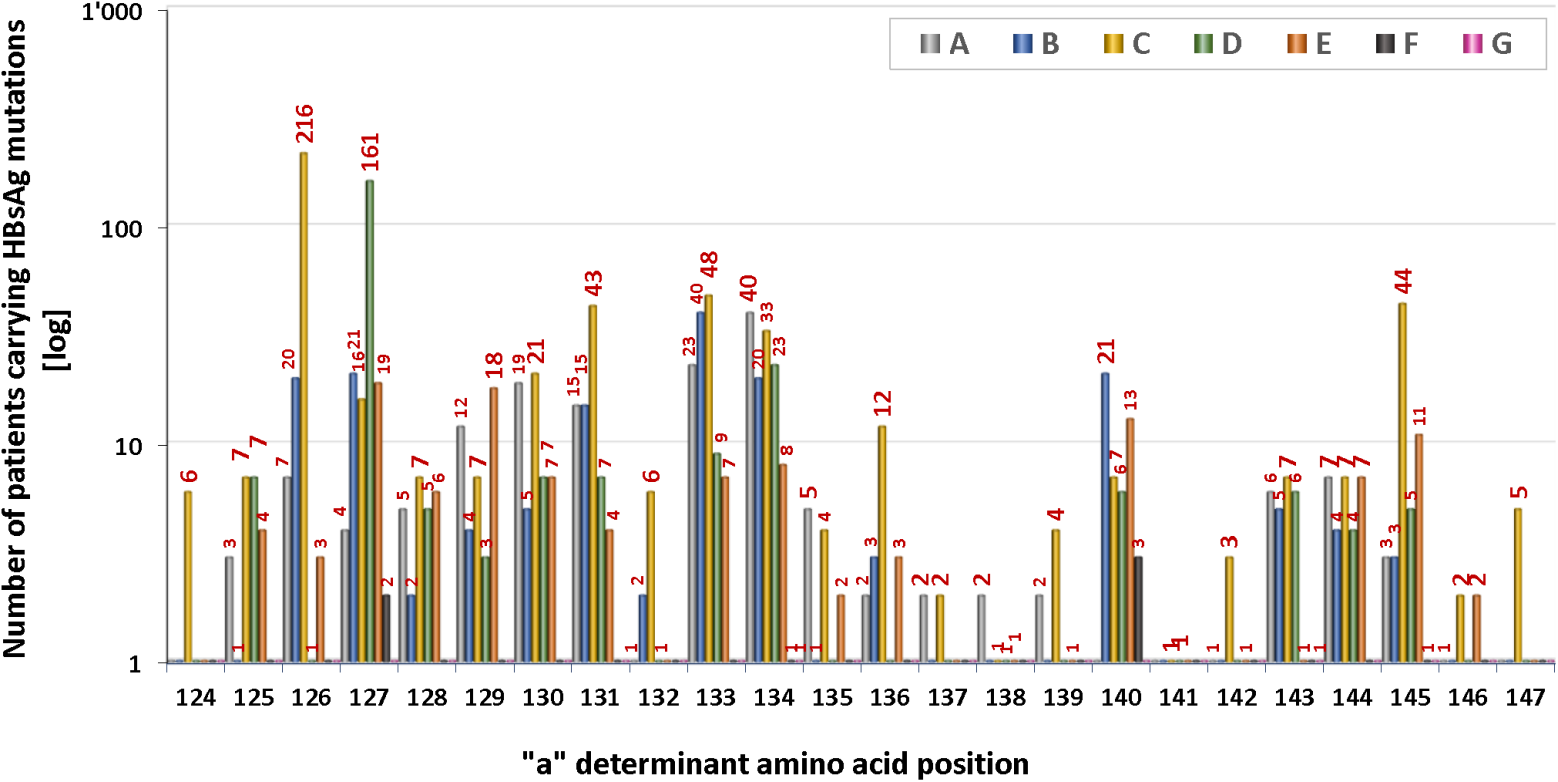
Distribution of HBV genotypes within the HBsAg MHR “a” determinant region (aa 124–147). HBV genotypes A-G are color coded. Genotype frequencies are shown for individual amino acid sites. Numbers above the columns represent total patient numbers.

We then determined the distribution of HBV genotypes for each individual amino acid site within the MHR “a” determinant region. We found a diverse pattern of HBV genotype variation across the entire “a” determinant region (Fig 5). At more than two thirds of the amino acid residues in this region (n = 17, 71%) mutations of at least four different HBV genotypes were found. The maximum number of mutation bearing genotypes per amino acid site was six (genotypes A-F), which was observed at aa127 and aa134. Only two sites, the “a” determinant region flanking aa124 and aa147, showed variation in only a single genotype (HBV genotype C). Moreover, we noted that the maximum mutation frequency of each of the HBV genotypes A-E localized to a distinct amino acid site within the “a” determinant region. These genotype-specific sites of maximum mutation frequency included aa134 (genotype A), aa 133 (genotype B), aa126 (genotype C), aa127 (genotype D), and aa127 (genotype E), respectively.

Mutations of the MHR “a” determinant that are associated with immune escape and diagnostic failure are of particular clinical importance. As expected, we identified all of the previously reported “a” determinant mutations (n = 51) that have been associated with clinical and diagnostic complications in our four-continent cohort (S8 Table). The well-established M133T dimorphism, which was found in 65 subjects, and the I126S mutation (64 subjects) showed by far the highest frequency in our global cohort. The classical G145R immune escape mutant, which we identified in 34 subjects, ranked among the top 5 of the pool MHR mutations that have previously been shown to be of clinical relevance.

### Continental distribution of “a” determinant mutations

**Fig 6:**
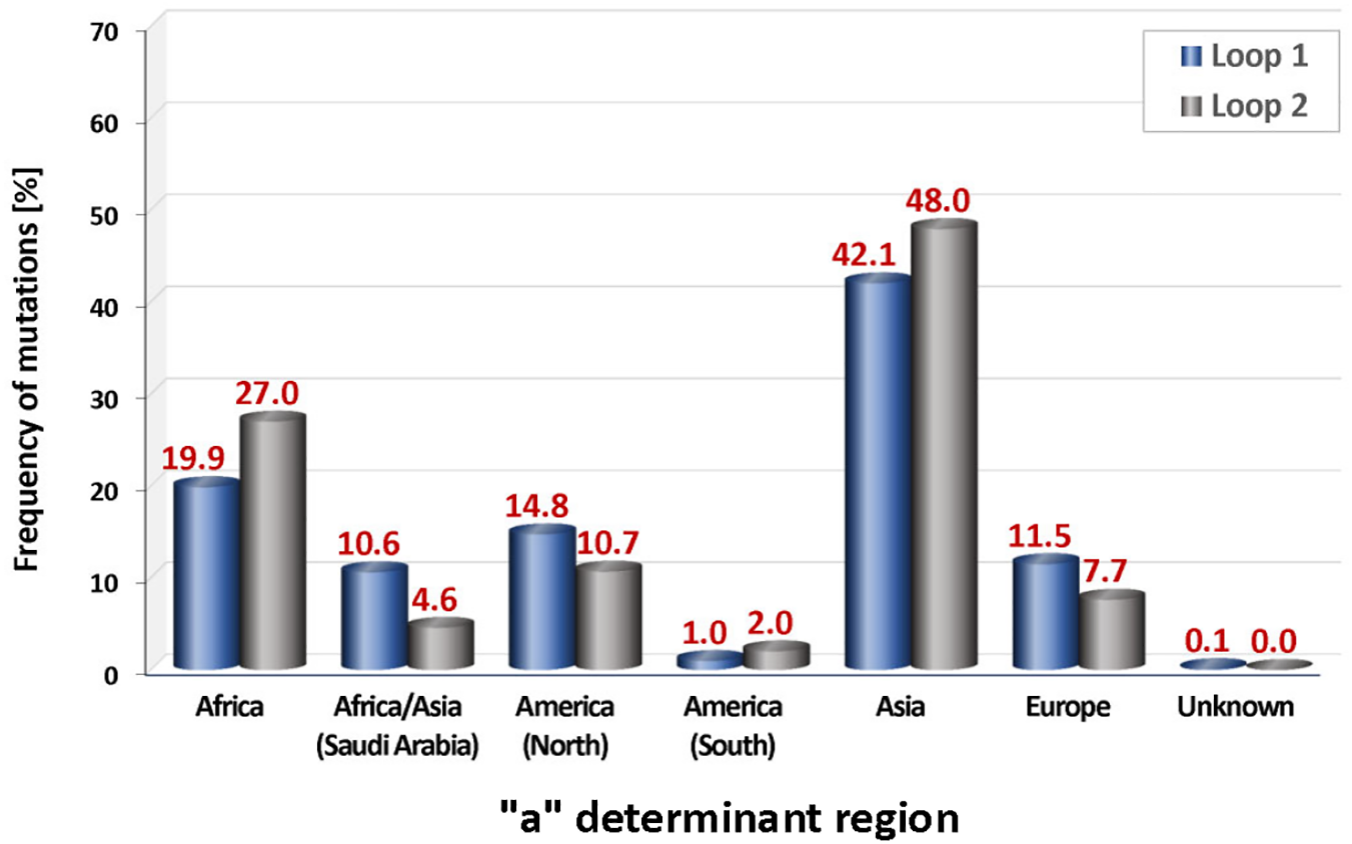
Relative frequencies of mutations in the “a” determinant region (aa 124–147) in four continental regions. Frequencies are shown separately for loop 1 (aa124-137, blue) and loop 2 (aa139-147, gray).

Geographic comparison of the relative frequencies of mutations of the “a” determinant region revealed marked differences between the various continental regions. By far the highest percentage of “a” determinant region mutations was observed in our Asian cohort (Fig 6). Subdomain analysis demonstrated that 42% of the observed “a” determinant region mutations in Asian HBV subjects localized to loop 1 and 48% to loop 2. The African HBV cohort showed second highest mutation frequencies in the “a” determinant region (loop 1: 20%, loop 2: 27%), which was followed by the North American (loop 1: 15%, loop 2: 11%), the European (loop 1: 12%, loop 2: 8%) and the African/Asian (loop 1: 11%, loop 2: 5%) cohorts. The lowest frequency of “a” determinant region mutations was found in subjects from South America (loop 1: 1%, loop 2: 2%). In the cohorts from Africa, Asia and South America more mutations were identified in loop 2 than in loop 1, whereas the reverse scenario was the case in HBV subjects from North America, Europe and Africa/Asia (Saudi Arabia).

### Identification of novel HBsAg MHR mutations

**Fig 7:**
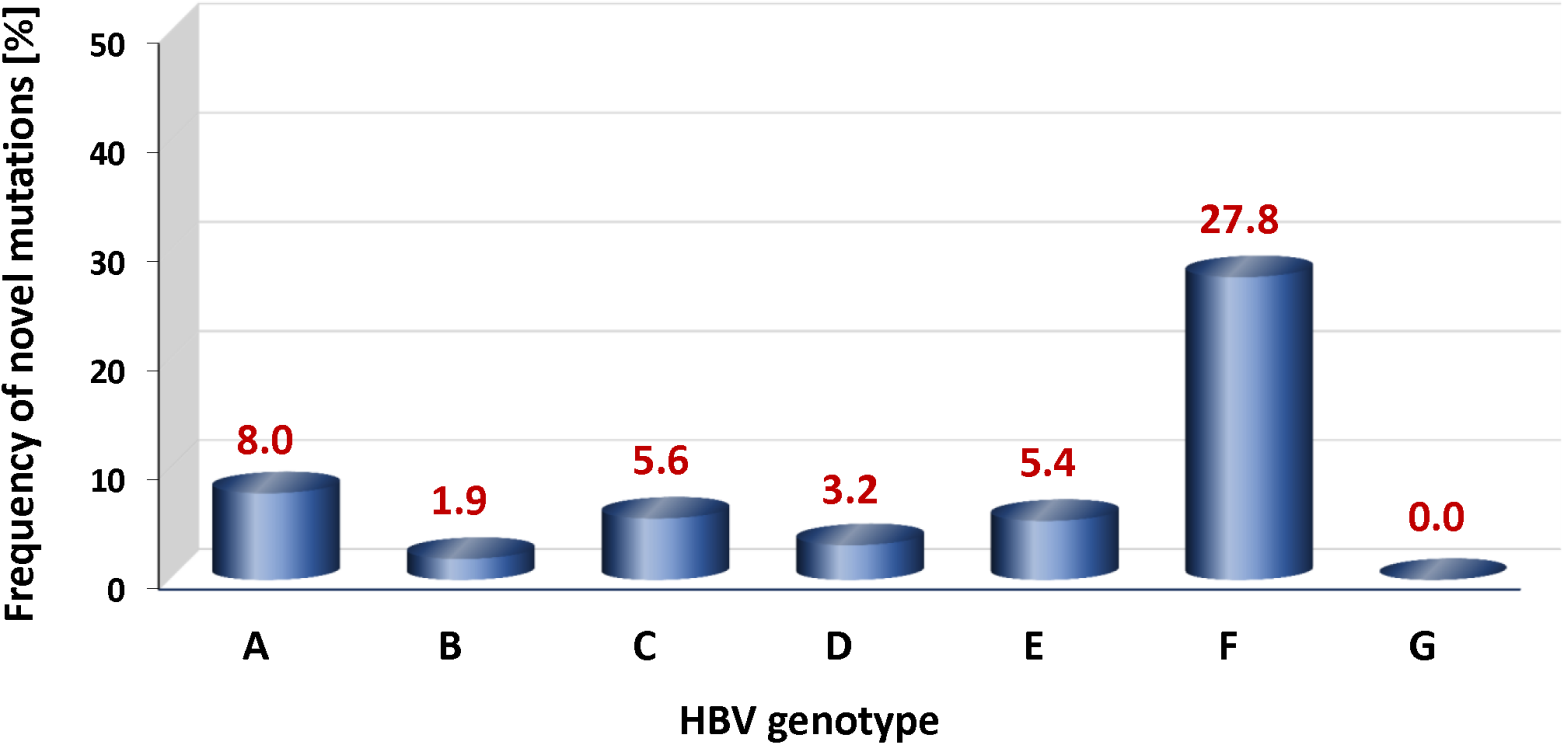
Frequencies of 62 novel HBsAg MHR mutations (aa 99–170) in various HBV genotypes. Frequencies are defined as the percentage of patients carrying a novel mutation in a given HBV genotype.

We next determined the fraction of as yet unknown mutations within the pool of HBsAg MHR mutations which we identified in this global survey (n = 345). A mutation was defined as “novel” when it met all of the following criteria: genetic variation that (i) induces a single amino acid change or premature chain termination, (ii) occurs in any HBV genotype and (iii) has not previously been reported. To identify such mutations, we conducted a detailed and systematic inventory of the published literature (PubMed), available public databases and the search engine Google^TM^ (S9 Table). Using this approach, we found that 62 of the identified mutations have thus far not been reported (Table 2). The 62 novel mutations were detected in a minor subgroup (n = 143) of the pool of ultra-deep sequenced subjects (n = 1391) and are scattered over 41 of the 72 codons that encode the HBsAg MHR. The vast majority of the 62 novel mutations (n = 54, 87%) induce amino acid exchanges and only 8 mutations introduce a premature stop codon into the HBV S gene. Notably, a 23% sub-fraction (n = 14) of the novel mutations localizes to the “a” determinant region, of which 11 induce amino acid substitutions. In 12 cases the previously unknown MHR mutations were present in at least two different subjects and also in distinct HBV genotypes (Y100*, Q101L, G102A, V106A, P108S, L109H, I110S, L110P, S117C, K122Q, G145V, G145*) (Table 2). Surprisingly, the by far highest frequency of novel mutations was observed in the HBV genotype F (14%). This was followed by genotypes A (5%), C and E (each 4%), respectively, whereas no novel mutations were detected in the HBV genotype G (Fig 7). The disproportionately high rate of novel MHR mutations in HBV F most likely reflects the fact that only limited sequence information is currently available on this genotype, which is most prevalent in South and Central American countries [8, 34].

**Table 2:** Synopsis of novel HBV MHR mutations (n = 62) identified in four continental populations and their prevalence in various HBV genotypes. Mutations in the “a” determinant region are in italics, those in its flanking regions are highlighted **bold**.

aa pos	Novel mutation	Number of patients carrying HBsAg MHR mutations at aa position	Number of patients carrying the HBsAg MHR mutation	Number of patients carrying the HBsAg MHR mutation in the indicated HBV genotype						
				A	B	C	D	E	F	G
				n = 25	n = 10	n = 41	n = 8	n = 10	n = 5	n = 0
**99**	**D99A**	**3**	**2**	**-**	**-**	**2**	**-**	**-**	**-**	**-**
	**D99G**		**1**	**-**	**1**	**-**	**-**	**-**	**-**	**-**
**100**	Y100*	**2**	**2**	**-**	**-**	**-**	**1**	**-**	**1**	**-**
**101**	**Q101L**	**6**	**5**	**1**	**-**	**3**	**-**	**1**	**-**	**-**
	**Q101N**		**1**	**-**	**-**	**1**	**-**	**-**	**-**	**-**
**102**	**G102A**	**6**	**4**	**2**	**-**	**2**	**-**	**-**	**-**	**-**
	**G102N**		**1**	**1**	**-**	**-**	**-**	**-**	**-**	**-**
	**G102V**		**1**	**1**	**-**	**-**	**-**	**-**	**-**	**-**
**104**	**L104V**	**1**	**1**	**1**	**-**	**-**	**-**	**-**	**-**	**-**
**105**	**P105S**	**2**	**2**	**-**	**-**	**-**	**-**	**2**	**-**	**-**
**106**	**V106A**	**6**	**5**	**3**	**2**	**-**	**-**	**-**	**-**	**-**
	**V106I**		**1**	**-**	**-**	**-**	**-**	**1**	**-**	**-**
**107**	C107*	**1**	**1**	**-**	**-**	**1**	**-**	**-**	**-**	**-**
**108**	**P108L**	**4**	**1**	**-**	**-**	**1**	**-**	**-**	**-**	**-**
	**P108S**		**2**	**1**	**1**	**-**	**-**	**-**	**-**	**-**
	**P108T**		**1**	**1**	**-**	**-**	**-**	**-**	**-**	**-**
**109**	**L109H**	**2**	**2**	**1**	**1**	**-**	**-**	**-**	**-**	**-**
**110**	**I110N**	**9**	**2**	**-**	**-**	**-**	**2**	**-**	**-**	**-**
	**I110S**		**4**	**1**	**1**	**-**	**-**	**2**	**-**	**-**
	**L110P**		**3**	**-**	**-**	**3**	**-**	**-**	**-**	**-**
**111**	**P111A**	**3**	**1**	**-**	**-**	**1**	**-**	**-**	**-**	**-**
	**P111N**		**1**	**-**	**-**	**1**	**-**	**-**	**-**	**-**
	**P111R**		**1**	**-**	**1**	**-**	**-**	**-**	**-**	**-**
**112**	**G112N**	**2**	**1**	**-**	**-**	**-**	**1**	**-**	**-**	**-**
	**G112Q**		**1**	**-**	**-**	**1**	**-**	**-**	**-**	**-**
**113**	**T113K**	**1**	**1**	**-**	**-**	**1**	**-**	**-**	**-**	**-**
**114**	**S114K**	**4**	**1**	**-**	**-**	**-**	**-**	**1**	**-**	**-**
	**S114L**		**1**	**-**	**-**	**1**	**-**	**-**	**-**	**-**
	**S114N**		**1**	**-**	**-**	**-**	**-**	**1**	**-**	**-**
	**T114I**		**1**	**1**	**-**	**-**	**-**	**-**	**-**	**-**
**115**	**T115K**	**1**	**1**	**-**	**-**	**1**	**-**	**-**	**-**	**-**
**116**	**T116V**	**1**	**1**	**-**	**-**	**-**	**-**	**1**	**-**	**-**
**117**	**S117C**	**2**	**2**	**1**	**-**	**1**	**-**	**-**	**-**	**-**
**118**	**T118Q**	**2**	**2**	**2**	**-**	**-**	**-**	**-**	**-**	**-**
**119**	**G119V**	**1**	**1**	**1**	**-**	**-**	**-**	**-**	**-**	**-**
**120**	**P120I**	**1**	**1**	**-**	**-**	**1**	**-**	**-**	**-**	**-**
**121**	**C121N**	**4**	**1**	**-**	**-**	**1**	**-**	**-**	**-**	**-**
	**C121R**		**2**	**-**	**-**	**2**	**-**	**-**	**-**	**-**
	C121*		**1**	**-**	**-**	**-**	**-**	**-**	**1**	**-**
**122**	**K122G**	**8**	**1**	**-**	**-**	**1**	**-**	**-**	**-**	**-**
	**K122Q**		**6**	**1**	**-**	**5**	**-**	**-**	**-**	**-**
	**K122S**		**1**	**-**	**-**	**1**	**-**	**-**	**-**	**-**
**125**	*T125N*	**1**	**1**	**-**	**-**	**-**	**1**	**-**	**-**	**-**
**127**	*L127F*	**2**	**2**	**-**	**-**	**-**	**-**	**-**	**2**	**-**
**130**	*G130C*	**1**	**1**	**-**	**1**	**-**	**-**	**-**	**-**	**-**
**131**	*N131H*	**1**	**1**	**1**	**-**	**-**	**-**	**-**	**-**	**-**
**133**	*M133R*	**1**	**1**	**-**	**-**	**-**	**1**	**-**	**-**	**-**
**134**	*F134Q*	**1**	**1**	**-**	**1**	**-**	**-**	**-**	**-**	**-**
**136**	*S136A*	**2**	**1**	**-**	**1**	**-**	**-**	**-**	**-**	**-**
	S136*		**1**	**1**	**-**	**-**	**-**	**-**	**-**	**-**
**137**	C137*	**2**	**2**	**2**	**-**	**-**	**-**	**-**	**-**	**-**
**139**	*C139F*	**1**	**1**	**-**	**-**	**1**	**-**	**-**	**-**	**-**
**142**	*P142H*	**1**	**1**	**1**	**-**	**-**	**-**	**-**	**-**	**-**
**145**	*G145V*	**7**	**5**	**-**	**-**	**3**	**2**	**-**	**-**	**-**
	G145*		**2**	**-**	**-**	**1**	**-**	**-**	**1**	**-**
**147**	*C147S*	**1**	**1**	**-**	**-**	**1**	**-**	**-**	**-**	**-**
**150**	**I150F**	**1**	**1**	**1**	**-**	**-**	**-**	**-**	**-**	**-**
**154**	S154*	**1**	**1**	**-**	**-**	**1**	**-**	**-**	**-**	**-**
**162**	**L162P**	**1**	**1**	**1**	**-**	**-**	**-**	**-**	**-**	**-**
**167**	S167*	**1**	**1**	**1**	**-**	**-**	**-**	**-**	**-**	**-**
**169**	**R169C**	**1**	**1**	**-**	**-**	**-**	**-**	**1**	**-**	**-**
**170**	**F170Y**	**1**	**1**	**-**	**-**	**1**	**-**	**-**	**-**	**-**

* Stop codon

**Fig 8:**
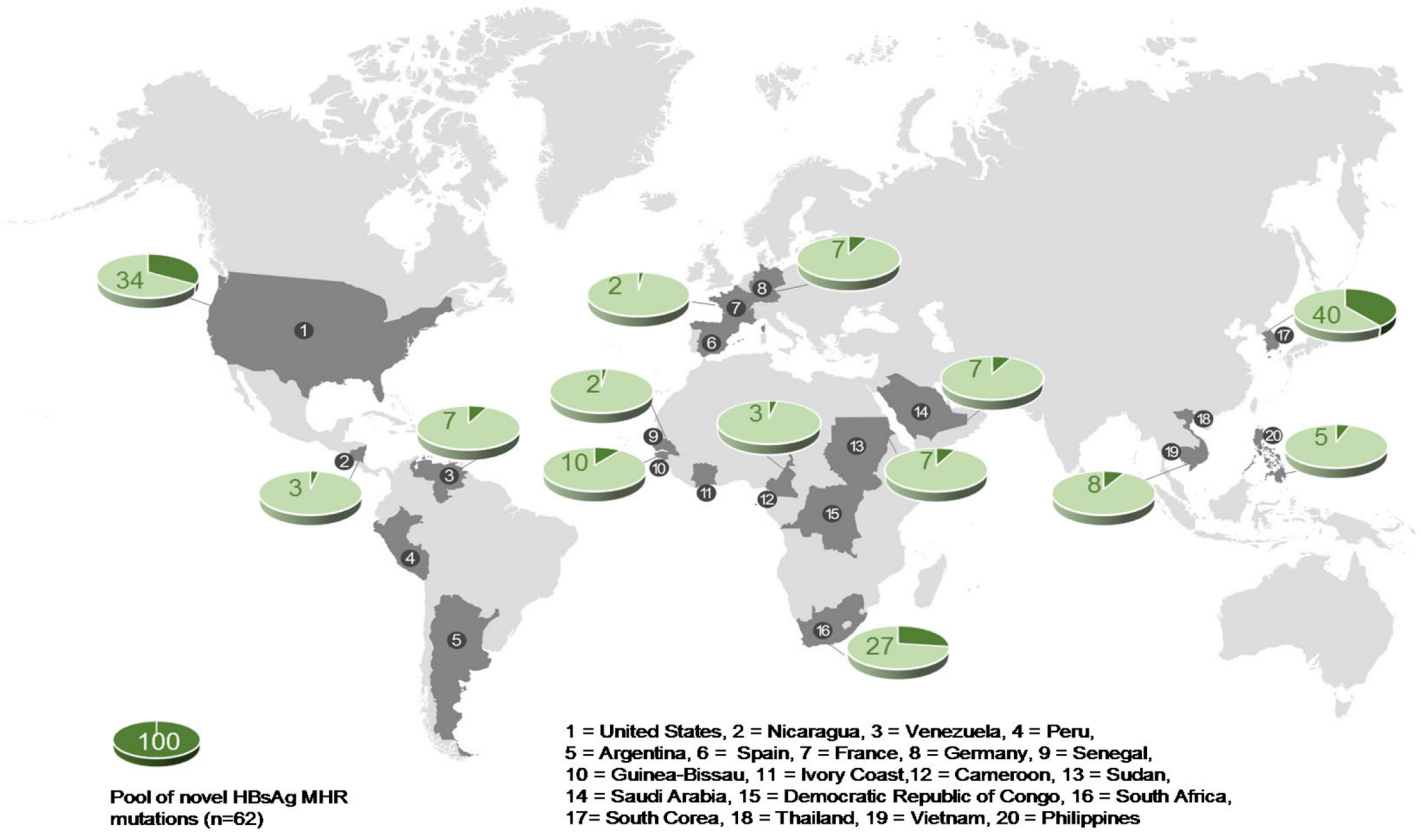
Global distribution of the novel HBsAg MHR mutations. The relative contribution (%, dark green sectors) to the total pool of 62 novel mutations is shown for all countries in which previously unknown mutants were identified (n = 14). Note that individual percentages do not add up to 100% because various countries share mutations. For more details see S10 and S11 Tables.

The 62 novel mutations were identified in 14 out of the 20 countries that were included in this study. Analysis of the geographic distribution revealed that they were scattered across all four continents investigated (Fig 8). Almost one fifth (n = 11) of all novel mutations were present in more than one continent (S10 Table). Among these, a group of four HBsAg MHR mutations were found in subjects who originated from three different continents suggesting high prevalence worldwide. These included the mutations Q101L, I110S, K122Q, and G145V, respectively.

Importantly, three populations, South Korea (n = 25, 40.3%), USA (n = 21, 33.9%), and South Africa (n = 17, 27.4%), respectively, contributed the most to the pool of novel MHR mutations (S11 Table). Remarkably, these three cohorts shared only minor fractions of the novel mutations (South Korea vs. USA: n = 4, South Korea vs. South Africa: n = 2, South Africa vs. USA: n = 3, S10 Table) indicating regional clustering of certain surface protein mutations.

**Fig 9:**
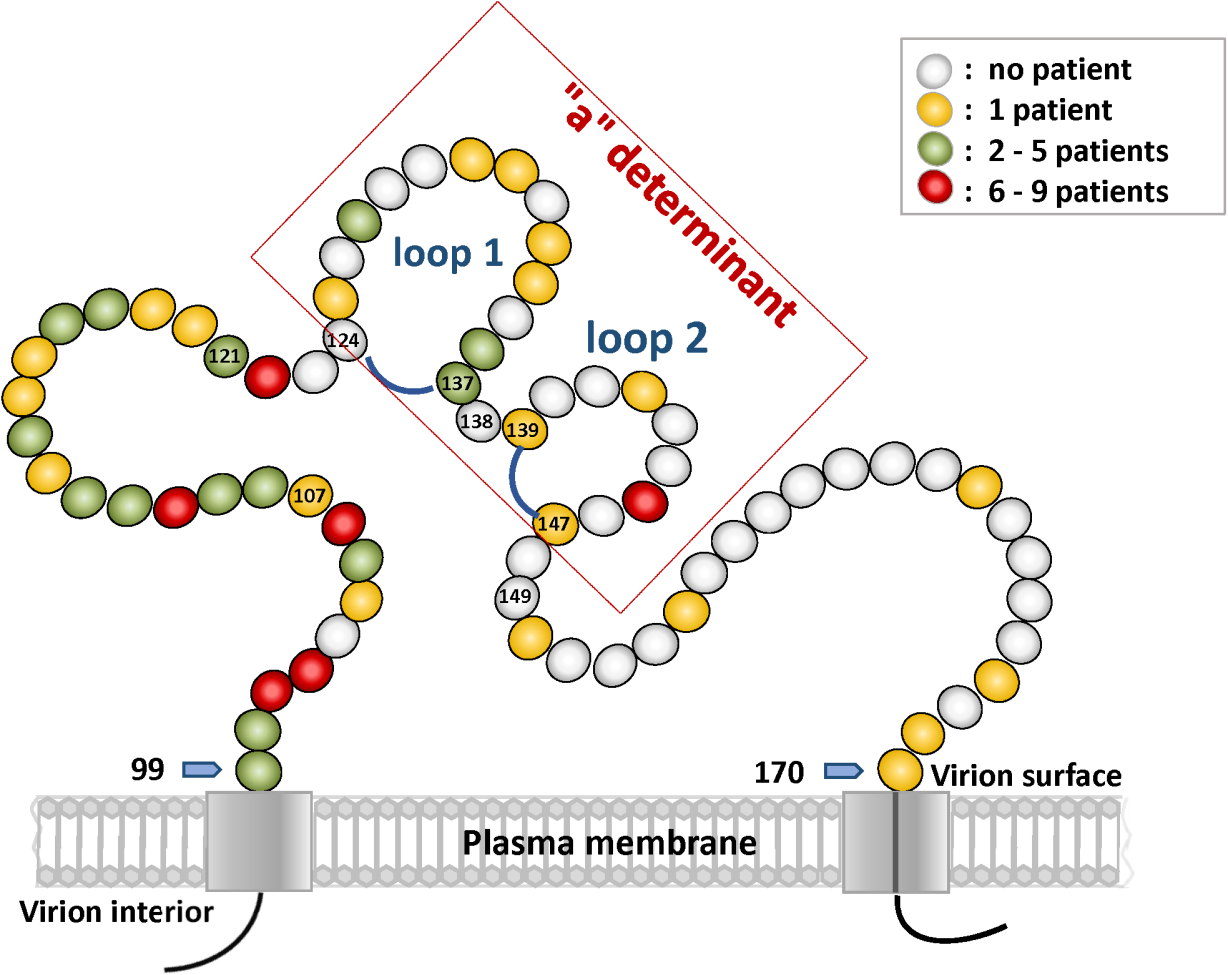
Localization of the 62 novel amino acid exchanges within the HBsAg MHR (aa 99–170). Novel mutations are color coded to show their relative frequency (yellow, green, red). The “a” determinant region, composed of loop 1 (aa124-137) and loop 2 (aa139-147), is boxed. The start and end of the MHR are highlighted by blue arrows. Numbered balls designate cytosine amino acid residues.

Finally, we found that the frequencies of previously unknown mutations within the MHR differed markedly between individual amino acid positions. They ranged from occurrence in a single subject to presence in up to 9 subjects (Fig 9). Within the “a” determinant region amino acid position 145 showed the highest occurrence among the newly identified MHR mutations. Outside of this domain, five additional “hot spots” for novel mutations were found at aa101, aa102, aa106, aa110, and aa122, respectively, which are all localized upstream of the “a” determinant.

## Discussion

This is the first genotyping study using massive parallel sequencing that explores the diversity of the HBV surface antigen at the global level. It relies on a GS Junior + based next generation sequencing technique that enabled the robust and highly sensitive assessment of variations in a large portion (>700 bp) of the HBV S gene in one single read. Using this approach, we identified in subjects from four continents an HBV genotype pool that encodes 345 distinct HBsAg MHR mutations, of which 62 were previously unknown. Next to the remarkably high genetic diversity of the HBsAg worldwide, we found that the global distribution of MHR mutations exhibits marked geographical differences with a particularly high degree of previously unrecognized mutants in South Korea, the USA and South Africa.

A current survey on 54 genotyping studies published during the past two decades reveals that the total number of HBsAg positive subjects in which genotyping of the HBsAg MHR has as yet been performed amounts to app. 7100 with a mean cohort size of 134 subjects (S12 Table). It also demonstrates that the vast majority of the past genotyping studies relied on conventional direct sequencing and focussed on only single ethnic populations. To date, only a few HBsAg genotyping reports have been published that are based on next generation sequencing and the cohorts investigated in these studies are of very limited size [35–37]. Our study population encompassing 1391 subjects constitutes the largest (multi-ethnic) cohort of HBsAg positive individuals in which genotyping of the HBV S gene has been performed and in which the technique of ultra-deep sequencing was utilized. It adds another 20% to the worldwide pool of genotyped HBsAg positive subjects.

The first major finding of this study is the strikingly high frequency of MHR variation in the global cohort. We find that on average 73% of all HBV positive subjects tested carry HBsAg MHR mutants with only minor variation across all age groups. In contrast, the mean MHR mutation frequency observed in comparable cohorts in the published literature is 26% (ranging from 2% to 95%) (S12 Table). This indicates that the global variation frequency in the HBsAg MHR is threefold higher than previously reported. Consistent with previous studies, our results also demonstrate that the frequency of MHR variation is highest in Asian populations.

The vast majority of studies that aimed at assessing the prevalence of MHR mutants routinely relied on conventional Sanger sequencing. In this study, we used the much more sensitive technique of ultra-deep sequencing [31]. Therefore, it is likely that the strikingly high MHR variation frequency we found in our mixed global cohort merely reflects the use of a more sensitive sequencing technique. This view is strongly supported by a most recent ultra-deep sequencing study that reports the same prevalence of MHR mutations (73%), which we also observed, in a small cohort of 11 Indonesian subjects with chronic hepatitis [37]. Theoretically, it is also possible that the high MHR mutation rate found in our study is the effect of sampling bias. However, the considerable size and the marked ethno-geographic heterogeneity of our cohort makes this possibility very unlikely.

The second important finding of our study is the demonstration that almost 60% of the subjects in our global cohort carried more than one HBV MHR mutant in their circulation. This high degree of intra-individual variation is remarkable in unselected populations and contrasts with previous large-scale studies in unselected HBV subjects from the USA (n = 946) and France (n = 940) using conventional Sanger sequencing which showed that the fraction of multiple MHR mutations was only <2% [13, 30]. In keeping with this, a recent study in 256 Italian subjects reports a frequency of multiple MHR mutations of only 1% [38]. Our results suggest that, from a global perspective, the presence of multiple HBV surface protein mutations in unselected HBsAg positive individuals may be the rule rather than the exception. It also indicates that the intra-individual biologic diversity of the HBsAg has as yet been largely underestimated. More studies in multi-ethnic cohorts using massive parallel sequencing are required to systematically assess the true complexity of intra-individual HBsAg variation and HBV quasispecies in subjects with HBV infection worldwide.

We found marked geographical differences in the genetic diversity of the HBsAg between the various continental regions. By far the highest percentage of “a” determinant region mutations was observed in the Asian population followed by the African and the North American cohorts, respectively. In keeping with this, the majority of the novel HBsAg MHR mutations were identified in only three different populations, South Korea, USA, and South Africa, each representing one of these continents (Fig 8). This may, in part, be due to the fact that these three populations rank among the four largest ethnic sub-cohorts investigated in this study (>190 subjects). However, the observation that our largest sub-cohort (Vietnam) contributed strikingly little to the pool of novel mutations does not support the view that the rate of unknown MHR mutations detected in a population is merely an effect of sample size. Consistent with this, one of the smallest subject cohorts (Venezuela) exhibited by far the highest rate of novel MHR mutations. This strongly suggests that regional factors act as the critical determinants of HBsAg diversity.

The high degree of MHR diversity we found in the US American cohort is surprising in light of a recent large genotyping study, using direct sequencing, in which variation in the MHR was found in only 11% of all HBsAg positive subjects [13]. Because both in the latter work and this study unselected subjects from across the United States were analyzed, the high number of novel mutations detected in our US American cohort is rather the reflection of the high sensitivity of ultra-deep sequencing than an effect caused by sampling bias. The high rate of novel MHR mutations in the South African and South Korean cohorts is most likely attributable to the fact that, to this date, only limited HBV genotyping data are available for both ethnic regions [14, 27, 39–41].

Another important finding of our global HBsAg sequencing approach is the identification of a large number of novel MHR mutations. Altogether, we identified 345 distinct amino acid changes within the major hydrophilic region. This pool of mutations comprised all 51 known “a” determinant mutations that have been associated with diagnostic failure and immune escape. Literature research and internet data mining indicated that 62 of the identified 345 amino acid substitutions represent previously unrecognized MHR mutations. These thus extend the current pool of MHR mutations by 22%. Notably, 87% of these novel mutations induce amino acid substitutions which may potentially give rise to protein structure alterations. The densest accumulation of novel MHR mutations (68%) was noted in the peptide sequence upstream of the “a” determinant. In this region (aa99-123), novel mutations were identified at each single amino acid position with the exception of only two residues (aa103, aa123) (Fig 9). In contrast, markedly fewer new mutations were detected in the “a” determinant domain (22%), and yet less in the downstream flanking region of the “a” determinant (10%). Importantly, this asymmetric topologic distribution within the MHR was also observed for the entire pool of the 345 identified HBsAg mutations. Together, these findings clearly identify the “a” determinant upstream flanking region as the structurally most diverse domain of the HBsAg major hydrophilic region.

Amino acid changes in this region have previously been associated with immune escape or diagnostic failure as evidenced for substitutions at aa116, aa120, aa122 and aa123, respectively [42–44]. Notably, the K122R mutation was the most commonly observed variation in the present study. Given this and the fact that most work in the past has focussed on the “a” determinant, it will be challenging to determine the biological significance of mutations in the “a” determinant upstream flanking region and their impact on the sensitivity of HBV screening methods or the response to vaccination. In this context, the highly variable amino acid position 122 that has previously been implicated in the modulation of antigenicity [45] and for which we identified three novel mutations (K122/G/Q/S) appears to be of particular interest.

Because the “a” determinant constitutes the principal immunologic target of host defence and diagnostic testing, it will be important to determine which of the 14 novel genetic variants that induce residue changes in this region, or a combination thereof, escape diagnostic testing or immune surveillance. Among the novel “a” determinant mutations those localized at aa134 (first loop) and aa145 (located in the second loop at the same position as the quintessential G145R escape mutation) showed highest prevalence. They represent most promising candidates for further studies addressing the clinical implications of the newly identified “a” determinant mutations.

In this work, we specifically focused on the global assessment of genetic variation within the S gene. Because the HBV S gene is completely overlapped by the polymerase (POL) gene, genetic variants in the S gene potentially induce changes in the POL gene and *vice versa*. Several mutations of the POL gene have been described to mediate resistance to antiviral drugs [11]. Detection of these mutations even in minority strains has a strong impact on therapy management. In fact, drug-induced POL region mutations can indirectly induce diagnostic escape variations in the S gene [46]. Work is in progress to address the important question whether and to which degree the newly identified S gene variants impact the POL gene.

In summary, this first global next generation sequencing-based HBV genotyping study identified a remarkably high frequency of HBsAg MHR mutations in subjects with HBV infection worldwide. Despite its limitation to 20 distinct countries and their associated ethnicities, it revealed the existence of a large number of as yet unknown HBsAg mutants. Given the worldwide prevalence of HBV, this predicts that future genotyping studies relying on ultra-deep sequencing will uncover significant numbers of additional mutations in the HBV surface protein. Systematic assessment of the worldwide biological diversity of the HBV will provide a basis for better understanding the clinical complexity of this pandemic infection.

## Supporting information

S1 Fig Schematic representation of the two-step PCR strategy used to generate the universal tail amplicon library. (a) First round PCR targets the HBV-specific sequences and adds the universal tails (Univ-A, Univ-B). (b) Second round PCR targets the universal tails and adds the 454 adaptors (A, B), key and multiplex identifier (MID) sequences, respectively.
(DOC)

S2 Fig Amplified region of the HBV S gene. The HBV pre-S1, pre-S2 and S genes are represented by arrows. MHR, HBsAg major hydrophilic region. HBV-fw, forward primer; HBV-rev, reverse primer.
(DOC)

S1 Table Validation of the HBsAg MHR ultra-deep sequencing assay. Shown are the results from a comparison between ultra-deep sequencing and conventional Sanger sequencing in which the same PCR amplification products of the HBsAg 731 bp target region were used as templates. A total of 333 previously known mutations present in 44 selected HBsAg positive serum samples were determined independently. Note that the concordance between ultra-deep sequencing and the Sanger method was 100% (bottom).
(DOC)

S2 Table List of genotype-specific reference sequences used for bioinformatic analyses [32]. (DOC)

S3 Table Countries and continents from which HBV patients were recruited to this study. HBV endemicities are color coded according to Schweitzer et al [2].
(DOC)

S4 Table Percentage of patients carrying HBsAg MHR mutations stratified by age. (DOC)

S5 Table Gender distribution of MHR mutations. (DOC)

S6 Table Number and proportion of patients carrying HBsAg MHR mutations shown for each genotype. *Please note: The number differs from the total number of patients (n = 1391) because more than one HBV genotype was detected in several patients.
(DOC)

S7 Table Synopsis of all mutations identified in the HBsAg MHR (aa99-170) that result in an amino acid substitution or premature chain termination within at least one genotype (n = 345). Mutations are sorted in descending order by frequency and are categorized by genotype (A-G). Mutations that are assigned to the MHR “a” determinant region are localized at aa124-147.
(DOC)

S8 Table Prevalence of 51 MHR “a” determinant region amino acid dimorphisms, which have previously been associated with clinical and diagnostic complications in four continental populations [13, 19, 25, 47–54]. Patient numbers are categorized by HBV genotypes (A-G).
(DOCX)

S9 Table Identification of previously unknown HBsAg MHR mutations. All 345 MHR mutations detected in this study were subjected to a systematic search in the published literature (PubMed; a subanalysis is summarized in S10 Table), available public databases and the search engine Google^TM^. Retrieved publication records or pertinent information are shown for individual mutations if available.
(DOC)

S10 Table Origin of individual patients bearing newly identified 62 HBsAg MHR mutations. Mutations that are present in three different continents are highlighted (blue). *Stop codon.
(DOC)

S11 Table Geographic distribution of 62 novel HBsAg MHR mutations (in descending order according to novel variants per cohort). The three cohorts contributing most to the pool of novel variants are highlighted (blue). Note that individual percentages do not add up to 100% because various countries share variants.
(DOC)

S12 Table Synopsis of HBsAg MHR genotyping studies published 1995–2016 (n = 54, MHR variant frequencies in descending order). (DOCX)
