# Supplementary material for: Ultra-deep sequencing reveals high prevalence and broad structural diversity of hepatitis B surface antigen mutations in a global population
Source: PLoS One. 2017 May 4;12(5):e0172101. doi: 10.1371/journal.pone.0172101 (PMC5417417; doi:10.1371/journal.pone.0172101)

**Supplemental Figure 1**

Schematic representation of the two-step PCR strategy that was used to generate the universal tail amplicon library. (a) First round PCR targets the HBV-specific sequences and adds the universal tails (Univ-A, Univ-B). (b) Second round PCR targets the universal tails and adds the 454 adaptors (A, B), key and multiplex identifier (MID) sequences, respectively.


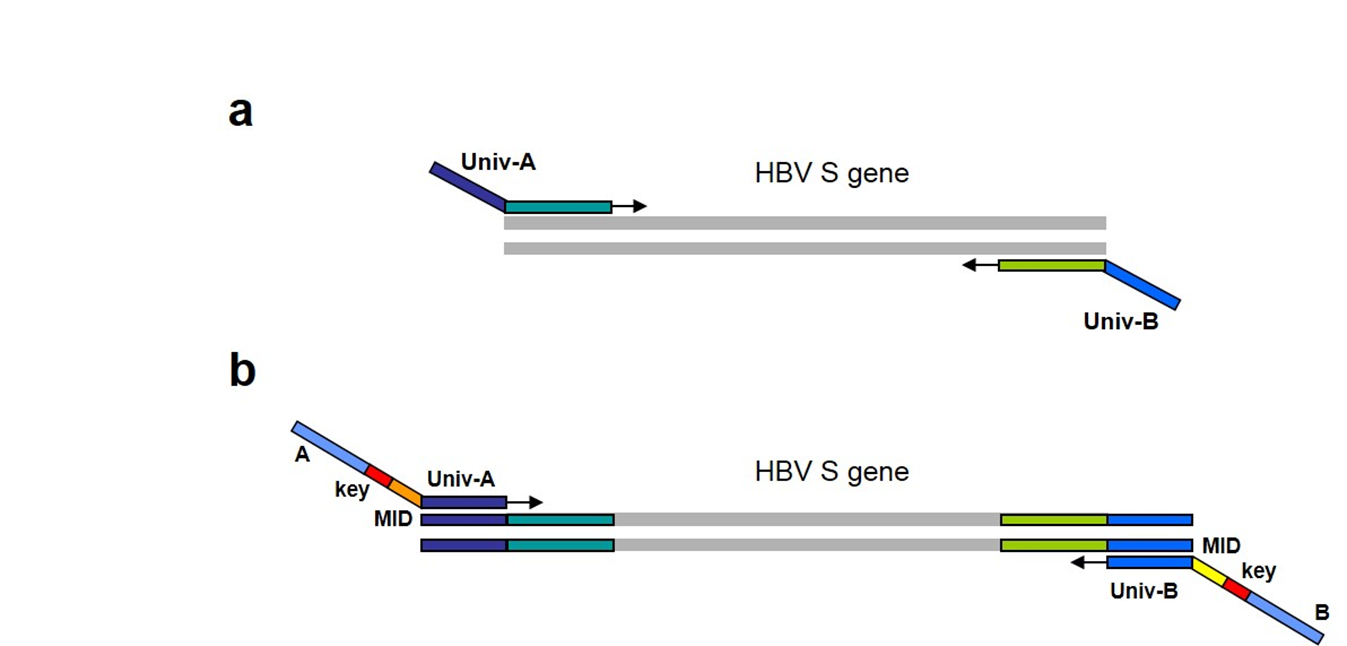

Supplement: S1 Fig — (a) First round PCR targets the HBV-specific sequences and adds the universal tails (Univ-A, Univ-B). (b) Second round PCR targets the universal tails and adds the 454 adaptors (A, B), key and multiplex identifier (MID) sequences, respectively. (DOC) [file pone.0172101.s001.doc]
