# Supplementary material for: Ultra-deep sequencing reveals high prevalence and broad structural diversity of hepatitis B surface antigen mutations in a global population
Source: PLoS One. 2017 May 4;12(5):e0172101. doi: 10.1371/journal.pone.0172101 (PMC5417417; doi:10.1371/journal.pone.0172101)

**Supplemental Figure 2**

Amplified region of the HBV S gene. The HBV pre-S1, pre-S2 and S genes are represented by arrows. MHR, HBsAg major hydrophilic region. HBV-fw, forward primer; HBV-rev, reverse primer.


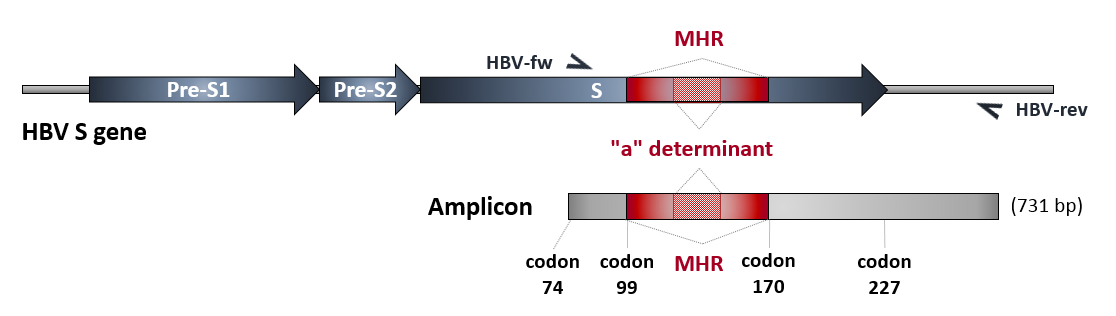

Supplement: S2 Fig — The HBV pre-S1, pre-S2 and S genes are represented by arrows. MHR, HBsAg major hydrophilic region. HBV-fw, forward primer; HBV-rev, reverse primer. (DOC) [file pone.0172101.s002.doc]
