# Supplementary material for: Ultra-deep sequencing reveals high prevalence and broad structural diversity of hepatitis B surface antigen mutations in a global population
Source: PLoS One. 2017 May 4;12(5):e0172101. doi: 10.1371/journal.pone.0172101 (PMC5417417; doi:10.1371/journal.pone.0172101)
Supplement: S1 Table — Shown are the results from a comparison between ultra-deep sequencing and conventional Sanger sequencing in which the same PCR amplification products of the HBsAg 731 bp target region were used as templates. A total of 333 previously known mutations present in 44 selected HBsAg positive serum samples were determined independently. Note that the concordance between ultra-deep sequencing and the Sanger method was 100% (bottom). (DOC) [file pone.0172101.s003.doc]

**Supplemental Table 1**

Validation of the HBsAg MHR ultra-deep sequencing assay. Shown are the results from a comparison between ultra-deep sequencing and conventional Sanger sequencing in which the same PCR amplification products of the HBsAg 731 bp target region were used as templates. A total of 333 previously known mutations present in 44 selected HBsAg positive serum samples were determined independently. Note that the concordance between ultra-deep sequencing and the Sanger method was 100% (bottom).

|  | **Ultra-deep sequencing** | | **Sanger sequencing** | **Evaluation UDS vs. Sanger** |
| --- | --- | --- | --- | --- |
| **Sample ID** | **Coverage** | **Mutation detected** | **Mutation detected** | **passed/ failed** |
| **MID01** | 425 | **S204N** | **S204NS** | **passed** |
| **MID01** | 425 | **P211Q** | **P211HPQ** | **passed** |
| **MID01** | 425 | **L220I** | **L220IL** | **passed** |
| **MID01** | 425 | **P211H** | **P211HPQ** | **passed** |
| **MID01** | 425 | **S207R** | **S207RS** | **passed** |
| **MID01** | 425 | **Y200F** | **Y200FY** | **passed** |
| **MID01** | 425 | **C256G** | **C256CG** | **passed** |
| **MID01** | 425 | **L157M** | **L157M** | **passed** |
| **MID01** | 425 | **N248H** | **N248H** | **passed** |
| **MID01** | 425 | **Y135S** | **Y135S** | **passed** |
| **MID01** | 425 | **T127P** | **T127P** | **passed** |
| **MID03** | 1640 | **S207N** | **S207N** | **passed** |
| **MID03** | 1640 | **N248H** | **N248H** | **passed** |
| **MID03** | 1640 | **L91I** | **L91I** | **passed** |
| **MID03** | 1640 | **T127P** | **T127P** | **passed** |
| **MID05** | 434 | **N248H** | **N248H** | **passed** |
| **MID05** | 434 | **C256S** | **C256S** | **passed** |
| **MID05** | 434 | **Y135S** | **Y135S** | **passed** |
| **MID05** | 434 | **Q215S** | **Q215S** | **passed** |
| **MID05** | 434 | **T127P** | **T127P** | **passed** |
| **MID05** | 434 | **S207R** | **S207R** | **passed** |
| **MID06** | 1059 | **V190A** | **V190AV** | **passed** |
| **MID06** | 1059 | **Y206S** | **Y206S** | **passed** |
| **MID06** | 1059 | **K212R** | **K212R** | **passed** |
| **MID06** | 1059 | **S204G** | **S204G** | **passed** |
| **MID06** | 1059 | **V112A** | **V112A** | **passed** |
| **MID06** | 1059 | **G202A** | **G202A** | **passed** |
| **MID06** | 1059 | **S207N** | **S207N** | **passed** |
| **MID06** | 1059 | **S210K** | **S210K** | **passed** |
| **MID06** | 1059 | **S219A** | **S219A** | **passed** |
| **MID06** | 1059 | **N248H** | **N248H** | **passed** |
| **MID06** | 1059 | **I266R** | **I266R** | **passed** |
| **MID06** | 1059 | **C256S** | **C256S** | **passed** |
| **MID06** | 1059 | **L209V** | **L209V** | **passed** |
| **MID06** | 1059 | **L217R** | **L217R** | **passed** |
| **MID06** | 1059 | **Y135S** | **Y135S** | **passed** |
| **MID06** | 1059 | **T127P** | **T127P** | **passed** |
| **MID06** | 1059 | **N118D** | **N118D** | **passed** |
| **MID07** | 425 | **Y200F** | **Y200FL** | **passed** |
| **MID07** | 425 | **A211T** | **A211AT** | **passed** |
| **MID07** | 425 | **I187L** | **I187IL** | **passed** |
| **MID07** | 425 | **K270T** | **K270KT** | **passed** |
| **MID07** | 425 | **I163V** | **I163IV** | **passed** |
| **MID07** | 425 | **S204N** | **S204DN** | **passed** |
| **MID07** | 425 | **V207M** | **V207MV** | **passed** |
| **MID07** | 425 | **M198I** | **M198IM** | **passed** |
| **MID07** | 425 | **Q149K** | **Q149KQ** | **passed** |
| **MID07** | 425 | **I266R** | **I266R** | **passed** |
| **MID07** | 425 | **Y200L** | **Y200FL** | **passed** |
| **MID07** | 425 | **L209V** | **L209LV** | **passed** |
| **MID07** | 425 | **S204D** | **S204DN** | **passed** |
| **MID07** | 425 | **K212R** | **K212KR** | **passed** |
| **MID07** | 425 | **N238H** | **N238HN** | **passed** |
| **MID07** | 425 | **Q215S** | **Q215PS** | **passed** |
| **MID07** | 425 | **S210R** | **S210R** | **passed** |
| **MID07** | 425 | **S219A** | **S219A** | **passed** |
| **MID07** | 425 | **Y206C** | **Y206C** | **passed** |
| **MID07** | 425 | **S207R** | **S207R** | **passed** |
| **MID07** | 425 | **C256S** | **C256S** | **passed** |
| **MID07** | 425 | **N248H** | **N248H** | **passed** |
| **MID07** | 425 | **Y135S** | **Y135S** | **passed** |
| **MID07** | 425 | **T127P** | **T127P** | **passed** |
| **MID08** | 682 | **Y135H** | **Y135H** | **passed** |
| **MID08** | 682 | **I266R** | **I266R** | **passed** |
| **MID08** | 682 | **Y257F** | **Y257F** | **passed** |
| **MID08** | 682 | **C256G** | **C256G** | **passed** |
| **MID08** | 682 | **K160R** | **K160R** | **passed** |
| **MID08** | 682 | **N248H** | **N248H** | **passed** |
| **MID08** | 682 | **H126R** | **H126R** | **passed** |
| **MID08** | 682 | **N131D** | **N131D** | **passed** |
| **MID08** | 682 | **V224A** | **V224A** | **passed** |
| **MID08** | 682 | **T118V** | **T118V** | **passed** |
| **MID08** | 682 | **A128V** | **A128V** | **passed** |
| **MID09** | 568 | **N248H** | **N248H** | **passed** |
| **MID09** | 568 | **Y135S** | **Y135S** | **passed** |
| **MID09** | 568 | **T127P** | **T127P** | **passed** |
| **MID12** | 440 | **F158L** | **F158FL** | **passed** |
| **MID12** | 440 | **S204N** | **S204NS** | **passed** |
| **MID12** | 440 | **C256G** | **C256CG** | **passed** |
| **MID12** | 440 | **N248H** | **N248H** | **passed** |
| **MID12** | 440 | **S207N** | **S207N** | **passed** |
| **MID12** | 440 | **H124N** | **H124N** | **passed** |
| **MID12** | 440 | **Q130P** | **Q130P** | **passed** |
| **MID12** | 440 | **Y135S** | **Y135S** | **passed** |
| **MID12** | 440 | **T127P** | **T127P** | **passed** |
| **MID13** | 840 | **C256G** | **C256CG** | **passed** |
| **MID13** | 840 | **I266R** | **I266R** | **passed** |
| **MID13** | 840 | **N248H** | **N248H** | **passed** |
| **MID13** | 840 | **Y135S** | **Y135S** | **passed** |
| **MID13** | 840 | **T127P** | **T127P** | **passed** |
| **MID14** | 957 | **F122L** | **F122FL** | **passed** |
| **MID14** | 957 | **L199V** | **L199LV** | **passed** |
| **MID14** | 957 | **Q215S** | **Q215S** | **passed** |
| **MID14** | 957 | **N238H** | **N238H** | **passed** |
| **MID14** | 957 | **N248H** | **N248H** | **passed** |
| **MID14** | 957 | **Y135S** | **Y135S** | **passed** |
| **MID16** | 899 | **Y206F** | **Y206FL** | **passed** |
| **MID16** | 899 | **V173M** | **V173MV** | **passed** |
| **MID16** | 899 | **L115V** | **L115LV** | **passed** |
| **MID16** | 899 | **S204R** | **S204KR** | **passed** |
| **MID16** | 899 | **S204K** | **S204KR** | **passed** |
| **MID16** | 899 | **V214A** | **V214AV** | **passed** |
| **MID16** | 899 | **Y206L** | **Y206FL** | **passed** |
| **MID16** | 899 | **Y200S** | **Y200SY** | **passed** |
| **MID16** | 899 | **S207N** | **S207NS** | **passed** |
| **MID16** | 899 | **P237T** | **P237T** | **passed** |
| **MID16** | 899 | **S213T** | **S213T** | **passed** |
| **MID16** | 899 | **N248H** | **N248H** | **passed** |
| **MID16** | 899 | **I266R** | **I266R** | **passed** |
| **MID16** | 899 | **Y135S** | **Y135S** | **passed** |
| **MID16** | 899 | **T127P** | **T127P** | **passed** |
| **MID18** | 358 | **S193L** | **S193L** | **passed** |
| **MID18** | 358 | **H124Y** | **H124Y** | **passed** |
| **MID18** | 358 | **S213T** | **S213T** | **passed** |
| **MID18** | 358 | **S204R** | **S204R** | **passed** |
| **MID18** | 358 | **Y206C** | **Y206C** | **passed** |
| **MID18** | 358 | **N131K** | **N131K** | **passed** |
| **MID18** | 358 | **Q267H** | **Q267H** | **passed** |
| **MID18** | 358 | **I266R** | **I226R** | **passed** |
| **MID18** | 358 | **Q149K** | **Q149K** | **passed** |
| **MID18** | 358 | **P237T** | **P237T** | **passed** |
| **MID18** | 358 | **T123S** | **T123S** | **passed** |
| **MID18** | 358 | **R153W** | **R153W** | **passed** |
| **MID18** | 358 | **Y257W** | **Y257W** | **passed** |
| **MID18** | 358 | **T127P** | **T127P** | **passed** |
| **MID18** | 358 | **Y135S** | **Y135S** | **passed** |
| **MID18** | 358 | **N248H** | **N248H** | **passed** |
| **MID19** | 636 | **P237S** | **P237PS** | **passed** |
| **MID19** | 636 | **I208T** | **I208IT** | **passed** |
| **MID19** | 636 | **N248H** | **N248H** | **passed** |
| **MID19** | 636 | **Y135S** | **Y135S** | **passed** |
| **MID19** | 636 | **H124Y** | **H124Y** | **passed** |
| **MID19** | 636 | **T127P** | **T127P** | **passed** |
| **MID20** | 476 | **P217Q** | **P217PQ** | **passed** |
| **MID20** | 476 | **D263N** | **D263DN** | **passed** |
| **MID20** | 476 | **L228F** | **L228FL** | **passed** |
| **MID20** | 476 | **L216*** | **L216*L** | **passed** |
| **MID20** | 476 | **S174N** | **S174N** | **passed** |
| **MID20** | 476 | **A166V** | **A166V** | **passed** |
| **MID20** | 476 | **N248H** | **N248H** | **passed** |
| **MID20** | 476 | **K270R** | **K270R** | **passed** |
| **MID20** | 476 | **L115V** | **L115V** | **passed** |
| **MID20** | 476 | **I266L** | **I266L** | **passed** |
| **MID20** | 476 | **Y135S** | **Y135S** | **passed** |
| **MID20** | 476 | **S143L** | **S143L** | **passed** |
| **MID20** | 476 | **T127P** | **T127P** | **passed** |
| **MID21** | 700 | **I266R** | **I266IR** | **passed** |
| **MID21** | 700 | **L213S** | **L213S** | **passed** |
| **MID21** | 700 | **N238H** | **N238H** | **passed** |
| **MID21** | 700 | **S207T** | **S207T** | **passed** |
| **MID21** | 700 | **Q215H** | **Q215H** | **passed** |
| **MID21** | 700 | **C256G** | **C256G** | **passed** |
| **MID21** | 700 | **N248H** | **N248H** | **passed** |
| **MID21** | 700 | **Y135S** | **Y135S** | **passed** |
| **MID21** | 700 | **T127P** | **T127P** | **passed** |
| **MID22** | 372 | **P217L** | **P217LP** | **passed** |
| **MID22** | 372 | **I208T** | **I208IT** | **passed** |
| **MID22** | 372 | **S106P** | **S106PS** | **passed** |
| **MID22** | 372 | **L229M** | **L229LMV** | **passed** |
| **MID22** | 372 | **Y200C** | **Y200CY** | **passed** |
| **MID22** | 372 | **E218D** | **E218D** | **passed** |
| **MID22** | 372 | **F220L** | **F220FL** | **passed** |
| **MID22** | 372 | **S204R** | **S204RS** | **passed** |
| **MID22** | 372 | **L213I** | **L213I** | **passed** |
| **MID22** | 372 | **S213T** | **S213ST** | **passed** |
| **MID22** | 372 | **N248H** | **N248H** | **passed** |
| **MID22** | 372 | **F221Y** | **F221Y** | **passed** |
| **MID22** | 372 | **R274Q** | **R274Q** | **passed** |
| **MID22** | 372 | **Y206F** | **Y206F** | **passed** |
| **MID22** | 372 | **I266R** | **I266R** | **passed** |
| **MID22** | 372 | **Y135S** | **Y135S** | **passed** |
| **MID22** | 372 | **T127P** | **T127P** | **passed** |
| **MID25** | 920 | **T148I** | **T148IT** | **passed** |
| **MID25** | 920 | **N248H** | **N248H** | **passed** |
| **MID25** | 920 | **D263N** | **D263N** | **passed** |
| **MID25** | 920 | **Y135S** | **Y135S** | **passed** |
| **MID25** | 920 | **T127P** | **T127P** | **passed** |
| **MID27** | 644 | **V184A** | **V184AV** | **passed** |
| **MID27** | 644 | **V96G** | **V96GV** | **passed** |
| **MID27** | 644 | **C256S** | **C256CS** | **passed** |
| **MID27** | 644 | **F122V** | **F122V** | **passed** |
| **MID27** | 644 | **N248H** | **N248H** | **passed** |
| **MID27** | 644 | **Y135S** | **Y135S** | **passed** |
| **MID27** | 644 | **T127P** | **T127P** | **passed** |
| **MID28** | 398 | **G112N** | **G112N** | **passed** |
| **MID28** | 398 | **I121C** | **I121C** | **passed** |
| **MID28** | 398 | **R120K** | **R120K** | **passed** |
| **MID28** | 398 | **S113A** | **S113A** | **passed** |
| **MID28** | 398 | **Y135S** | **Y135S** | **passed** |
| **MID28** | 398 | **T127L** | **T127L** | **passed** |
| **MID28** | 398 | **Q215S** | **Q215S** | **passed** |
| **MID28** | 398 | **N248H** | **N248H** | **passed** |
| **MID28** | 398 | **G127D** | **G127D** | **passed** |
| **MID28** | 398 | **G119T** | **G119T** | **passed** |
| **MID28** | 398 | **S207R** | **S207R** | **passed** |
| **MID28** | 398 | **K241Q** | **K241Q** | **passed** |
| **MID28** | 398 | **C256S** | **C256S** | **passed** |
| **MID28** | 398 | **V177A** | **V177A** | **passed** |
| **MID28** | 398 | **T126I** | **T126I** | **passed** |
| **MID28** | 398 | **Q129P** | **Q129P** | **passed** |
| **MID28** | 398 | **I150T** | **I150T** | **passed** |
| **MID28** | 398 | **L175S** | **L175S** | **passed** |
| **MID28** | 398 | **F122D** | **F122D** | **passed** |
| **MID28** | 398 | **N123R** | **N123R** | **passed** |
| **MID28** | 398 | **H126Q** | **H126Q** | **passed** |
| **MID28** | 398 | **L145M** | **L145M** | **passed** |
| **MID28** | 398 | **S114T** | **S114T** | **passed** |
| **MID28** | 398 | **T118R** | **T118R** | **passed** |
| **MID28** | 398 | **Y206F** | **Y206F** | **passed** |
| **MID28** | 398 | **T115G** | **T115G** | **passed** |
| **MID28** | 398 | **M133T** | **M133T** | **passed** |
| **MID28** | 398 | **P151L** | **P151L** | **passed** |
| **MID28** | 398 | **V184A** | **V184A** | **passed** |
| **MID29** | 723 | **T128I** | **T128IT** | **passed** |
| **MID29** | 723 | **P120S** | **P120PS** | **passed** |
| **MID29** | 723 | **P217L** | **P217LP** | **passed** |
| **MID29** | 723 | **L209W** | **L209LW** | **passed** |
| **MID29** | 723 | **F221Y** | **F221FY** | **passed** |
| **MID29** | 723 | **L213I** | **L213IL** | **passed** |
| **MID29** | 723 | **F212S** | **F212SY** | **passed** |
| **MID29** | 723 | **S207N** | **S207NS** | **passed** |
| **MID29** | 723 | **F212Y** | **F212SY** | **passed** |
| **MID29** | 723 | **I266R** | **I266R** | **passed** |
| **MID29** | 723 | **H124Y** | **H124Y** | **passed** |
| **MID29** | 723 | **N248H** | **N248H** | **passed** |
| **MID29** | 723 | **T127P** | **T127P** | **passed** |
| **MID29** | 723 | **Y135S** | **Y135S** | **passed** |
| **MID32** | 1144 | **N118D** | **N118DN** | **passed** |
| **MID32** | 1144 | **Q149K** | **Q149K** | **passed** |
| **MID32** | 1144 | **N248H** | **N248H** | **passed** |
| **MID32** | 1144 | **Y135S** | **Y135S** | **passed** |
| **MID32** | 1144 | **T127P** | **T127P** | **passed** |
| **MID33** | 2273 | **F122V** | **F122IV** | **passed** |
| **MID33** | 2273 | **S219A** | **S219AS** | **passed** |
| **MID33** | 2273 | **S210R** | **S210RS** | **passed** |
| **MID33** | 2273 | **L217R** | **L217LR** | **passed** |
| **MID33** | 2273 | **L209V** | **L209LV** | **passed** |
| **MID33** | 2273 | **F122I** | **F122IV** | **passed** |
| **MID33** | 2273 | **N248H** | **N248H** | **passed** |
| **MID33** | 2273 | **Y135S** | **Y135S** | **passed** |
| **MID33** | 2273 | **T127P** | **T127P** | **passed** |
| **MID34** | 605 | **I162V** | **I162IV** | **passed** |
| **MID34** | 605 | **L164M** | **L164LM** | **passed** |
| **MID34** | 605 | **L229F** | **L229F** | **passed** |
| **MID34** | 605 | **C221F** | **C221F** | **passed** |
| **MID34** | 605 | **V194A** | **V194AV** | **passed** |
| **MID34** | 605 | **Q267H** | **Q267H** | **passed** |
| **MID34** | 605 | **Y257W** | **Y257W** | **passed** |
| **MID34** | 605 | **I163V** | **I163V** | **passed** |
| **MID34** | 605 | **Q149K** | **Q149K** | **passed** |
| **MID34** | 605 | **P237T** | **P237T** | **passed** |
| **MID34** | 605 | **N248H** | **N248H** | **passed** |
| **MID34** | 605 | **T189I** | **T189I** | **passed** |
| **MID34** | 605 | **R153W** | **R153W** | **passed** |
| **MID34** | 605 | **Y135S** | **Y135S** | **passed** |
| **MID34** | 605 | **T127P** | **T127P** | **passed** |
| **MID37** | 993 | **E271A** | **E271A** | **passed** |
| **MID37** | 993 | **Y135S** | **Y135S** | **passed** |
| **MID37** | 993 | **N248H** | **N248H** | **passed** |
| **MID37** | 993 | **T127P** | **T127P** | **passed** |
| **MID39** | 896 | **V184A** | **V184AV** | **passed** |
| **MID39** | 896 | **E271D** | **E271DE** | **passed** |
| **MID39** | 896 | **K212T** | **K212KT** | **passed** |
| **MID39** | 896 | **S204R** | **S204RS** | **passed** |
| **MID39** | 896 | **I208T** | **I208IT** | **passed** |
| **MID39** | 896 | **Y257H** | **Y257HY** | **passed** |
| **MID39** | 896 | **K270N** | **K270KN** | **passed** |
| **MID39** | 896 | **Y225F** | **Y225FY** | **passed** |
| **MID39** | 896 | **N139D** | **N139DN** | **passed** |
| **MID39** | 896 | **L145M** | **L145LM** | **passed** |
| **MID39** | 896 | **N238H** | **N238HN** | **passed** |
| **MID39** | 896 | **C256S** | **C256CS** | **passed** |
| **MID39** | 896 | **Y134F** | **Y134F** | **passed** |
| **MID39** | 896 | **Q101R** | **Q101R** | **passed** |
| **MID39** | 896 | **Y135S** | **Y135S** | **passed** |
| **MID39** | 896 | **N248H** | **N248H** | **passed** |
| **MID39** | 896 | **I266R** | **I266R** | **passed** |
| **MID39** | 896 | **T127P** | **T127P** | **passed** |
| **MID40** | 436 | **F221V** | **F221FILV** | **passed** |
| **MID40** | 436 | **S207T** | **S207NT** | **passed** |
| **MID40** | 436 | **Q215H** | **Q215HQ** | **passed** |
| **MID40** | 436 | **R114H** | **R114HR** | **passed** |
| **MID40** | 436 | **V106I** | **V106IV** | **passed** |
| **MID40** | 436 | **Y206L** | **Y206FHLY** | **passed** |
| **MID40** | 436 | **F221I** | **F221FILV** | **passed** |
| **MID40** | 436 | **F212L** | **F212FL** | **passed** |
| **MID40** | 436 | **S210R** | **S210KNRS** | **passed** |
| **MID40** | 436 | **V214A** | **V214AV** | **passed** |
| **MID40** | 436 | **S204N** | **S204NS** | **passed** |
| **MID40** | 436 | **F221L** | **F221FILV** | **passed** |
| **MID40** | 436 | **C256G** | **C256CG** | **passed** |
| **MID40** | 436 | **S210K** | **S210KNRS** | **passed** |
| **MID40** | 436 | **L229V** | **L229LV** | **passed** |
| **MID40** | 436 | **F220L** | **F220FL** | **passed** |
| **MID40** | 436 | **S117P** | **S117PS** | **passed** |
| **MID40** | 436 | **N238H** | **N238HN** | **passed** |
| **MID40** | 436 | **Q267T** | **Q267T** | **passed** |
| **MID40** | 436 | **S219A** | **S219AS** | **passed** |
| **MID40** | 436 | **T127I** | **T127I** | **passed** |
| **MID40** | 436 | **S207N** | **S207NT** | **passed** |
| **MID40** | 436 | **I233V** | **I233V** | **passed** |
| **MID40** | 436 | **E271D** | **E271D** | **passed** |
| **MID41** | 1271 | **T128N** | **T128N** | **passed** |
| **MID41** | 1271 | **P120T** | **P120PT** | **passed** |
| **MID41** | 1271 | **S174N** | **S174NS** | **passed** |
| **MID41** | 1271 | **S207T** | **S207NT** | **passed** |
| **MID41** | 1271 | **Q215H** | **Q215HQ** | **passed** |
| **MID41** | 1271 | **S207N** | **S207NT** | **passed** |
| **MID41** | 1271 | **S204K** | **S204KR** | **passed** |
| **MID41** | 1271 | **S204R** | **S204KR** | **passed** |
| **MID41** | 1271 | **L199V** | **L199V** | **passed** |
| **MID41** | 1271 | **N248H** | **N248H** | **passed** |
| **MID41** | 1271 | **S213T** | **S213T** | **passed** |
| **MID41** | 1271 | **Y135S** | **Y135S** | **passed** |
| **MID41** | 1271 | **T127P** | **T127P** | **passed** |
| **MID42** | 475 | **Q215H** | **Q215HQ** | **passed** |
| **MID42** | 475 | **S207T** | **S207NT** | **passed** |
| **MID42** | 475 | **V214A** | **V214AV** | **passed** |
| **MID42** | 475 | **Y206H** | **Y206HY** | **passed** |
| **MID42** | 475 | **N238H** | **N238H** | **passed** |
| **MID42** | 475 | **S207N** | **S207NT** | **passed** |
| **MID42** | 475 | **F122V** | **F122V** | **passed** |
| **MID42** | 475 | **I266R** | **I266R** | **passed** |
| **MID42** | 475 | **C256S** | **C256S** | **passed** |
| **MID42** | 475 | **N248H** | **N248H** | **passed** |
| **MID42** | 475 | **Y135S** | **Y135S** | **passed** |
| **MID42** | 475 | **T127P** | **T127P** | **passed** |
| **MID44** | 1809 | **M133T** | **S210RS** | **passed** |
| **MID44** | 1809 | **I269F** | **T127P** | **passed** |
| **MID44** | 1809 | **S219A** | **S219AS** | **passed** |
| **MID44** | 1809 | **N248H** | **N248H** | **passed** |
| **MID44** | 1809 | **Y135S** | **Y135S** | **passed** |
| **MID44** | 1809 | **T127P** | **S193L** | **passed** |
|  |  |  |  |  |
|  |  |  | **UDS vs.** |  |
|  |  |  | **Sanger sequencing** |  |
|  |  |  | **Concordance:** | **100%** |
|  |  |  |  | (333/333) |
