# Supplementary material for: Ultra-deep sequencing reveals high prevalence and broad structural diversity of hepatitis B surface antigen mutations in a global population
Source: PLoS One. 2017 May 4;12(5):e0172101. doi: 10.1371/journal.pone.0172101 (PMC5417417; doi:10.1371/journal.pone.0172101)
Supplement: S2 Table — (DOC) [file pone.0172101.s004.doc]

**Supplemental Table 2**

List of genotype-specific reference sequences used for bioinformatic analyses (ref. No. 32 in the main text)

| **Subtype** | **GeneBank Accession Number** |
| --- | --- |
| **A1** | U87734 |
| **A2** | AY168427 |
| **B1** | AB073855 |
| **B2** | AY217359 |
| **B3** | AB033554 |
| **B4** | AB100695 |
| **C1** | AB014368 |
| **C2** | AF209393 |
| **C3** | AF208876 |
| **C4** | AB048704 |
| **D1** | AY161157 |
| **D2** | Z35716 |
| **D3** | AF061523 |
| **D4** | AB048701 |
| **E** | AB091262 |
| **F1** | AY264390 |
| **F2** | AY264396 |
| **G** | M74499 |
| **H** | AF369536 |
