# Supplementary material for: Ultra-deep sequencing reveals high prevalence and broad structural diversity of hepatitis B surface antigen mutations in a global population
Source: PLoS One. 2017 May 4;12(5):e0172101. doi: 10.1371/journal.pone.0172101 (PMC5417417; doi:10.1371/journal.pone.0172101)
Supplement: S3 Table — HBV endemicities are color coded according to Schweitzer et al [2]. (DOC) [file pone.0172101.s005.doc]

**Supplemental Table 3**

Countries and continents from which HBV patients were recruited to this study. HBV endemicities are color coded according to Schweitzer et al [2].

| **Country** | **Continent** | **Number of patients** |
| --- | --- | --- |
| Argentina | America (South) | 5 |
| Cameroon | Africa | 17 |
| Dem. Republic of Congo | Africa | 2 |
| France | Europe | 45 |
| Germany | Europe | 68 |
| Guinea-Bissau | Africa | 106 |
| Ivory Coast | Africa | 18 |
| Nicaragua | America (South) | 21 |
| Peru | America (South) | 3 |
| Philippines | Asia | 66 |
| Saudi Arabia | Africa/Asia (Saudi Arabia) | 80 |
| Senegal | Africa | 5 |
| South Africa | Africa | 197 |
| South Korea | Asia | 233 |
| Spain | Europe | 1 |
| Sudan | Africa | 51 |
| Thailand | Asia | 8 |
| United States | America (North) | 200 |
| Venezuela | America (South) | 6 |
| Vietnam | Asia | 258 |
| Unknown | Unknown | 1 |
| Total | | 1391 |

**HBV endemicity** low

lower intermediate

higher intermediate

high
