# Supplementary figures and images for: Ultra-deep sequencing reveals high prevalence and broad structural diversity of hepatitis B surface antigen mutations in a global population
Source: PLoS One. 2017 May 4;12(5):e0172101. doi: 10.1371/journal.pone.0172101 (PMC5417417; doi:10.1371/journal.pone.0172101)

**Supplemental Table 4**

Percentage of patients carrying HBsAg MHR mutations stratified by age.


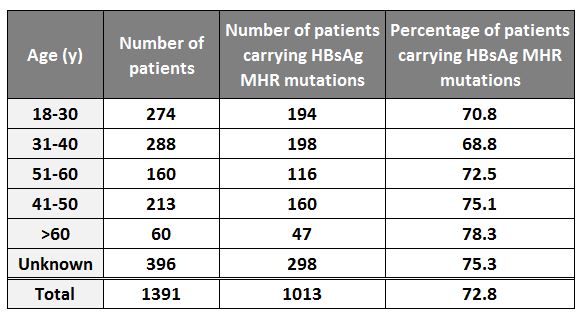

Supplement: S4 Table — (DOC) [file pone.0172101.s006.doc]

**Supplemental Table 5**

Gender distribution of MHR mutations.


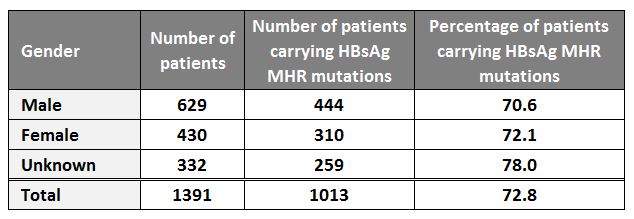

Supplement: S5 Table — (DOC) [file pone.0172101.s007.doc]
