# Supplementary material for: Ultra-deep sequencing reveals high prevalence and broad structural diversity of hepatitis B surface antigen mutations in a global population
Source: PLoS One. 2017 May 4;12(5):e0172101. doi: 10.1371/journal.pone.0172101 (PMC5417417; doi:10.1371/journal.pone.0172101)
Supplement: S6 Table — *Please note: The number differs from the total number of patients (n = 1391) because more than one HBV genotype was detected in several patients. (DOC) [file pone.0172101.s008.doc]

**Supplemental Table 6**

Number and proportion of patients carrying HBsAg MHR mutations shown for each genotype.

| **Genotype** | **Total number of patients** | **Number of patients carrying HBsAg MHR mutations** | **Percentage of patients carrying HBsAg MHR mutations** |
| --- | --- | --- | --- |
| **A** | **318** | **183** | **57.5%** |
| **B** | **325** | **294** | **90.5%** |
| **C** | **401** | **259** | **64.6%** |
| **D** | **176** | **176** | **100.0%** |
| **E** | **162** | **97** | **59.9%** |
| **F** | **13** | **8** | **61.5%** |
| **G** | **2** | **2** | **100.0%** |
| **Total** | **1397*** | **1019** | **72.9%** |

*Please note: The number differs from the total number of patients (n=1391) because more than one HBV genotype was detected in several patients.
