# Supplementary material for: Ultra-deep sequencing reveals high prevalence and broad structural diversity of hepatitis B surface antigen mutations in a global population
Source: PLoS One. 2017 May 4;12(5):e0172101. doi: 10.1371/journal.pone.0172101 (PMC5417417; doi:10.1371/journal.pone.0172101)
Supplement: S7 Table — Mutations are sorted in descending order by frequency and are categorized by genotype (A-G). Mutations that are assigned to the MHR “a” determinant region are localized at aa124-147. (DOC) [file pone.0172101.s009.doc]

**Supplemental Table 7**

Synopsis of all mutations identified in the HBsAg MHR (aa99-170) that result in an amino acid substitution or premature chain termination within at least one genotype (n=345). Mutations are sorted in descending order by frequency and are categorized by genotype (A-G). Mutations that are assigned to the MHR “a” determinant region are localized at aa124-147.

| **HBsAg mutation** | **Number of patients carrying HBsAg MHR mutations at aa position** | **HBV genotype** | | | | | | |
| --- | --- | --- | --- | --- | --- | --- | --- | --- |
|  |  | **A** | **B** | **C** | **D** | **E** | **F** | **G** |
|  |  | **n = 512** | **n = 621** | **n = 960** | **n = 407** | **n = 241** | **n = 36** | **n = 3** |
| **K122R** | **304** | **45** | **245** | **13** | **-** | **-** | **1** | **-** |
| **T127P** | **155** | **-** | **-** | **-** | **155** | **-** | **-** | **-** |
| **I126T** | **128** | **-** | **-** | **128** | **-** | **-** | **-** | **-** |
| **Y161F** | **86** | **38** | **48** | **-** | **-** | **-** | **-** | **-** |
| **A159V** | **66** | **23** | **18** | **25** | **-** | **-** | **-** | **-** |
| **M133T** | **65** | **12** | **11** | **36** | **3** | **3** | **-** | **-** |
| **I126S** | **63** | **-** | **-** | **63** | **-** | **-** | **-** | **-** |
| **T131N** | **54** | **-** | **12** | **33** | **6** | **3** | **-** | **-** |
| **Y100C** | **49** | **20** | **2** | **19** | **3** | **5** | **-** | **-** |
| **T118A** | **44** | **4** | **2** | **2** | **32** | **4** | **-** | **-** |
| **I110L** | **40** | **16** | **17** | **-** | **2** | **5** | **-** | **-** |
| **V168A** | **38** | **11** | **6** | **20** | **-** | **-** | **-** | **1** |
| **P120T** | **37** | **10** | **7** | **9** | **8** | **3** | **-** | **-** |
| **F134L** | **34** | **22** | **6** | **4** | **-** | **2** | **-** | **-** |
| **G145R** | **34** | **2** | **2** | **22** | **1** | **7** | **-** | **-** |
| **Q101R** | **32** | **2** | **3** | **18** | **6** | **3** | **-** | **-** |
| **R160K** | **28** | **-** | **-** | **28** | **-** | **-** | **-** | **-** |
| **M103I** | **28** | **13** | **-** | **13** | **1** | **1** | **-** | **-** |
| **Q101H** | **28** | **5** | **-** | **12** | **3** | **8** | **-** | **-** |
| **M133I** | **27** | **9** | **6** | **6** | **5** | **1** | **-** | **-** |
| **M133L** | **26** | **1** | **21** | **2** | **-** | **2** | **-** | **-** |
| **A128V** | **25** | **5** | **2** | **7** | **5** | **6** | **-** | **-** |
| **T140I** | **24** | **-** | **18** | **2** | **4** | **-** | **-** | **-** |
| **Q101K** | **24** | **3** | **1** | **18** | **2** | **-** | **-** | **-** |
| **P127T** | **23** | **1** | **14** | **8** | **-** | **-** | **-** | **-** |
| **G159A** | **23** | **-** | **-** | **-** | **15** | **8** | **-** | **-** |
| **P120S** | **23** | **-** | **16** | **1** | **6** | **-** | **-** | **-** |
| **E164G** | **22** | **8** | **7** | **1** | **2** | **4** | **-** | **-** |
| **T116N** | **22** | **5** | **1** | **10** | **1** | **3** | **2** | **-** |
| **T125M** | **21** | **3** | **1** | **7** | **6** | **4** | **-** | **-** |
| **L109P** | **19** | **2** | **6** | **3** | **2** | **6** | **-** | **-** |
| **D144E** | **19** | **3** | **2** | **6** | **4** | **4** | **-** | **-** |
| **F134I** | **19** | **6** | **3** | **9** | **-** | **1** | **-** | **-** |
| **A166V** | **19** | **1** | **6** | **4** | **4** | **2** | **2** | **-** |
| **G130N** | **18** | **9** | **1** | **5** | **1** | **2** | **-** | **-** |
| **Q129H** | **16** | **5** | **1** | **1** | **2** | **7** | **-** | **-** |
| **S167L** | **15** | **3** | **2** | **5** | **-** | **3** | **2** | **-** |
| **G130R** | **14** | **-** | **1** | **8** | **4** | **1** | **-** | **-** |
| **Y100S** | **14** | **5** | **1** | **7** | **-** | **1** | **-** | **-** |
| **F134V** | **14** | **2** | **2** | **8** | **-** | **1** | **1** | **-** |
| **G145E** | **13** | **-** | **-** | **9** | **2** | **2** | **-** | **-** |
| **I126N** | **13** | **-** | **-** | **13** | **-** | **-** | **-** | **-** |
| **T113S** | **13** | **-** | **-** | **13** | **-** | **-** | **-** | **-** |
| **F158S** | **12** | **4** | **7** | **1** | **-** | **-** | **-** | **-** |
| **I126V** | **12** | **-** | **-** | **12** | **-** | **-** | **-** | **-** |
| **T126A** | **12** | **1** | **11** | **-** | **-** | **-** | **-** | **-** |
| **G112R** | **12** | **4** | **-** | **1** | **3** | **4** | **-** | **-** |
| **G130S** | **12** | **10** | **-** | **2** | **-** | **-** | **-** | **-** |
| **Q129R** | **12** | **4** | **2** | **1** | **-** | **5** | **-** | **-** |
| **S114T** | **12** | **-** | **5** | **6** | **-** | **1** | **-** | **-** |
| **F134S** | **12** | **2** | **4** | **4** | **-** | **2** | **-** | **-** |
| **Y134F** | **12** | **-** | **-** | **-** | **11** | **-** | **-** | **1** |
| **G145A** | **12** | **1** | **1** | **8** | **-** | **2** | **-** | **-** |
| **T123A** | **11** | **-** | **3** | **5** | **1** | **2** | **-** | **-** |
| **T123N** | **11** | **-** | **-** | **10** | **-** | **1** | **-** | **-** |
| **L110I** | **11** | **-** | **-** | **9** | **-** | **-** | **2** | **-** |
| **T115N** | **11** | **-** | **-** | **3** | **1** | **6** | **1** | **-** |
| **L109Q** | **11** | **6** | **1** | **2** | **-** | **2** | **-** | **-** |
| **N131T** | **11** | **10** | **1** | **-** | **-** | **-** | **-** | **-** |
| **F170S** | **11** | **-** | **1** | **8** | **-** | **2** | **-** | **-** |
| **L127P** | **10** | **-** | **-** | **-** | **-** | **10** | **-** | **-** |
| **S140L** | **10** | **-** | **-** | **-** | **-** | **10** | **-** | **-** |
| **A166G** | **10** | **2** | **-** | **7** | **1** | **-** | **-** | **-** |
| **T126S** | **9** | **-** | **8** | **-** | **1** | **-** | **-** | **-** |
| **T126I** | **9** | **6** | **-** | **-** | **-** | **3** | **-** | **-** |
| **G130E** | **9** | **-** | **1** | **5** | **-** | **3** | **-** | **-** |
| **Q129L** | **9** | **2** | **-** | **1** | **1** | **5** | **-** | **-** |
| **R169H** | **9** | **4** | **-** | **1** | **3** | **-** | **1** | **-** |
| **S114A** | **9** | **-** | **-** | **6** | **-** | **3** | **-** | **-** |
| **Y100F** | **9** | **1** | **3** | **4** | **1** | **-** | **-** | **-** |
| **E164D** | **9** | **1** | **-** | **3** | **5** | **-** | **-** | **-** |
| **Y161S** | **9** | **2** | **7** | **-** | **-** | **-** | **-** | **-** |
| **G102S** | **9** | **4** | **-** | **4** | **-** | **-** | **1** | **-** |
| **T143M** | **9** | **5** | **4** | **-** | **-** | **-** | **-** | **-** |
| **P127S** | **8** | **1** | **4** | **3** | **-** | **-** | **-** | **-** |
| **L109I** | **8** | **3** | **3** | **1** | **-** | **1** | **-** | **-** |
| **G112E** | **8** | **1** | **1** | **4** | **-** | **2** | **-** | **-** |
| **D144A** | **8** | **3** | **2** | **1** | **-** | **2** | **-** | **-** |
| **T131P** | **8** | **-** | **1** | **6** | **-** | **1** | **-** | **-** |
| **G119E** | **8** | **1** | **1** | **5** | **-** | **-** | **1** | **-** |
| **K160R** | **7** | **3** | **1** | **1** | **2** | **-** | **-** | **-** |
| **S143L** | **7** | **-** | **-** | **1** | **5** | **1** | **-** | **-** |
| **S113A** | **7** | **2** | **1** | **-** | **1** | **3** | **-** | **-** |
| **T140S** | **7** | **-** | **2** | **5** | **-** | **-** | **-** | **-** |
| **S136F** | **7** | **-** | **1** | **5** | **-** | **1** | **-** | **-** |
| **S136Y** | **7** | **-** | **-** | **7** | **-** | **-** | **-** | **-** |
| **P120L** | **7** | **1** | **-** | **6** | **-** | **-** | **-** | **-** |
| **G119R** | **7** | **1** | **-** | **4** | **-** | **1** | **1** | **-** |
| **E164V** | **6** | **1** | **1** | **1** | **2** | **1** | **-** | **-** |
| **K122Q** | **6** | **1** | **-** | **5** | **-** | **-** | **-** | **-** |
| **T118K** | **6** | **-** | **-** | **6** | **-** | **-** | **-** | **-** |
| **I150T** | **6** | **3** | **-** | **2** | **-** | **1** | **-** | **-** |
| **S154P** | **6** | **-** | **-** | **5** | **1** | **-** | **-** | **-** |
| **F158L** | **6** | **-** | **-** | **-** | **3** | **3** | **-** | **-** |
| **A168V** | **6** | **-** | **-** | **-** | **1** | **5** | **-** | **-** |
| **P135L** | **6** | **2** | **1** | **3** | **-** | **-** | **-** | **-** |
| **S143T** | **6** | **-** | **-** | **5** | **-** | **-** | **-** | **1** |
| **A157G** | **6** | **2** | **3** | **-** | **1** | **-** | **-** | **-** |
| **F134Y** | **6** | **4** | **1** | **1** | **-** | **-** | **-** | **-** |
| **T131I** | **6** | **-** | **2** | **4** | **-** | **-** | **-** | **-** |
| **S113T** | **6** | **1** | **2** | **-** | **1** | **2** | **-** | **-** |
| **A159G** | **6** | **5** | **-** | **1** | **-** | **-** | **-** | **-** |
| **C149Y** | **6** | **3** | **-** | **2** | **1** | **-** | **-** | **-** |
| **T114S** | **6** | **5** | **1** | **-** | **-** | **-** | **-** | **-** |
| **W156L** | **6** | **-** | **2** | **2** | **-** | **-** | **2** | **-** |
| **S140T** | **6** | **-** | **-** | **-** | **-** | **3** | **3** | **-** |
| **P111L** | **6** | **1** | **-** | **4** | **1** | **-** | **-** | **-** |
| **W165L** | **5** | **-** | **1** | **1** | **-** | **-** | **3** | **-** |
| **P105R** | **5** | **2** | **2** | **1** | **-** | **-** | **-** | **-** |
| **M133S** | **5** | **-** | **1** | **4** | **-** | **-** | **-** | **-** |
| **P127H** | **5** | **2** | **1** | **2** | **-** | **-** | **-** | **-** |
| **C139Y** | **5** | **2** | **-** | **2** | **-** | **1** | **-** | **-** |
| **C107Y** | **5** | **-** | **-** | **3** | **2** | **-** | **-** | **-** |
| **S132F** | **5** | **-** | **1** | **4** | **-** | **-** | **-** | **-** |
| **V106A** | **5** | **3** | **2** | **-** | **-** | **-** | **-** | **-** |
| **C138Y** | **5** | **2** | **-** | **1** | **1** | **1** | **-** | **-** |
| **Y134N** | **5** | **-** | **-** | **-** | **5** | **-** | **-** | **-** |
| **F161Y** | **5** | **-** | **-** | **4** | **1** | **-** | **-** | **-** |
| **G102D** | **5** | **2** | **-** | **3** | **-** | **-** | **-** | **-** |
| **T123I** | **5** | **-** | **-** | **3** | **2** | **-** | **-** | **-** |
| **G145V** | **5** | **-** | **-** | **3** | **2** | **-** | **-** | **-** |
| **Q101L** | **5** | **1** | **-** | **3** | **-** | **1** | **-** | **-** |
| **I110S** | **4** | **1** | **1** | **-** | **-** | **2** | **-** | **-** |
| **I110M** | **4** | **3** | **1** | **-** | **-** | **-** | **-** | **-** |
| **T118V** | **4** | **-** | **-** | **-** | **3** | **1** | **-** | **-** |
| **T123S** | **4** | **-** | **-** | **-** | **3** | **1** | **-** | **-** |
| **K160N** | **4** | **1** | **-** | **-** | **1** | **1** | **1** | **-** |
| **L109V** | **4** | **2** | **1** | **-** | **-** | **-** | **1** | **-** |
| **L109M** | **4** | **1** | **2** | **-** | **1** | **-** | **-** | **-** |
| **T116A** | **4** | **1** | **-** | **3** | **-** | **-** | **-** | **-** |
| **Q129N** | **4** | **-** | **1** | **3** | **-** | **-** | **-** | **-** |
| **S136L** | **4** | **1** | **1** | **-** | **-** | **2** | **-** | **-** |
| **G159E** | **4** | **-** | **-** | **-** | **3** | **1** | **-** | **-** |
| **Y134H** | **4** | **-** | **-** | **-** | **4** | **-** | **-** | **-** |
| **P135H** | **4** | **3** | **-** | **-** | **-** | **1** | **-** | **-** |
| **S117N** | **4** | **-** | **-** | **3** | **1** | **-** | **-** | **-** |
| **S117R** | **4** | **1** | **-** | **3** | **-** | **-** | **-** | **-** |
| **T114K** | **4** | **4** | **-** | **-** | **-** | **-** | **-** | **-** |
| **L104W** | **4** | **1** | **-** | **1** | **2** | **-** | **-** | **-** |
| **C147Y** | **4** | **-** | **-** | **4** | **-** | **-** | **-** | **-** |
| **G102A** | **4** | **2** | **-** | **2** | **-** | **-** | **-** | **-** |
| **P120A** | **4** | **-** | **-** | **2** | **-** | **2** | **-** | **-** |
| **P111Q** | **4** | **1** | **-** | **2** | **-** | **1** | **-** | **-** |
| **T127S** | **3** | **-** | **-** | **-** | **3** | **-** | **-** | **-** |
| **I110V** | **3** | **1** | **1** | **-** | **-** | **1** | **-** | **-** |
| **K122N** | **3** | **-** | **-** | **3** | **-** | **-** | **-** | **-** |
| **K122E** | **3** | **1** | **-** | **2** | **-** | **-** | **-** | **-** |
| **C121S** | **3** | **1** | **-** | **2** | **-** | **-** | **-** | **-** |
| **L127V** | **3** | **-** | **-** | **-** | **-** | **3** | **-** | **-** |
| **S114P** | **3** | **-** | **-** | **1** | **1** | **1** | **-** | **-** |
| **L127I** | **3** | **-** | **-** | **-** | **-** | **3** | **-** | **-** |
| **L110P** | **3** | **-** | **-** | **3** | **-** | **-** | **-** | **-** |
| **R122K** | **3** | **-** | **-** | **-** | **3** | **-** | **-** | **-** |
| **T115I** | **3** | **-** | **-** | **3** | **-** | **-** | **-** | **-** |
| **G130A** | **3** | **-** | **1** | **1** | **1** | **-** | **-** | **-** |
| **Q129P** | **3** | **1** | **-** | **1** | **-** | **1** | **-** | **-** |
| **N146T** | **3** | **-** | **-** | **2** | **-** | **1** | **-** | **-** |
| **A157V** | **3** | **-** | **-** | **2** | **-** | **-** | **1** | **-** |
| **S132Y** | **3** | **1** | **-** | **1** | **1** | **-** | **-** | **-** |
| **P153L** | **3** | **-** | **-** | **1** | **-** | **-** | **2** | **-** |
| **F134K** | **3** | **-** | **2** | **-** | **-** | **1** | **-** | **-** |
| **F134H** | **3** | **-** | **-** | **3** | **-** | **-** | **-** | **-** |
| **F134C** | **3** | **1** | **-** | **2** | **-** | **-** | **-** | **-** |
| **C124S** | **3** | **-** | **-** | **3** | **-** | **-** | **-** | **-** |
| **S113P** | **3** | **-** | **-** | **-** | **1** | **2** | **-** | **-** |
| **E164A** | **3** | **1** | **-** | **-** | **-** | **2** | **-** | **-** |
| **T114P** | **3** | **3** | **-** | **-** | **-** | **-** | **-** | **-** |
| **T127L** | **2** | **-** | **-** | **-** | **2** | **-** | **-** | **-** |
| **C121R** | **2** | **-** | **-** | **2** | **-** | **-** | **-** | **-** |
| **T118M** | **2** | **-** | **-** | **1** | **1** | **-** | **-** | **-** |
| **P108H** | **2** | **-** | **-** | **-** | **2** | **-** | **-** | **-** |
| **P108S** | **2** | **1** | **1** | **-** | **-** | **-** | **-** | **-** |
| **T118S** | **2** | **1** | **-** | **1** | **-** | **-** | **-** | **-** |
| **T118R** | **2** | **-** | **-** | **-** | **2** | **-** | **-** | **-** |
| **T118Q** | **2** | **-** | **-** | **2** | **-** | **-** | **-** | **-** |
| **W165S** | **2** | **-** | **-** | **1** | **-** | **1** | **-** | **-** |
| **P105S** | **2** | **-** | **-** | **-** | **-** | **2** | **-** | **-** |
| **M133Q** | **2** | **-** | **1** | **-** | **-** | **1** | **-** | **-** |
| **S154L** | **2** | **2** | **-** | **-** | **-** | **-** | **-** | **-** |
| **I110N** | **2** | **-** | **-** | **-** | **2** | **-** | **-** | **-** |
| **P127L** | **2** | **-** | **-** | **2** | **-** | **-** | **-** | **-** |
| **P127A** | **2** | **-** | **1** | **1** | **-** | **-** | **-** | **-** |
| **T115P** | **2** | **2** | **-** | **-** | **-** | **-** | **-** | **-** |
| **L109H** | **2** | **1** | **1** | **-** | **-** | **-** | **-** | **-** |
| **P111_G112delinsP** | **2** | **-** | **-** | **-** | **2** | **-** | **-** | **-** |
| **G130K** | **2** | **-** | **-** | **-** | **1** | **1** | **-** | **-** |
| **S117C** | **2** | **1** | **-** | **1** | **-** | **-** | **-** | **-** |
| **D144G** | **2** | **1** | **-** | **-** | **-** | **1** | **-** | **-** |
| **M103V** | **2** | **1** | **-** | **1** | **-** | **-** | **-** | **-** |
| **R169P** | **2** | **1** | **-** | **1** | **-** | **-** | **-** | **-** |
| **L127F** | **2** | **-** | **-** | **-** | **-** | **-** | **2** | **-** |
| **S113_T114insT** | **2** | **2** | **-** | **-** | **-** | **-** | **-** | **-** |
| **Y100*** | **2** | **-** | **-** | **-** | **1** | **-** | **1** | **-** |
| **S132C** | **2** | **-** | **1** | **1** | **-** | **-** | **-** | **-** |
| **F134A** | **2** | **2** | **-** | **-** | **-** | **-** | **-** | **-** |
| **F134R** | **2** | **1** | **-** | **1** | **-** | **-** | **-** | **-** |
| **F134T** | **2** | **-** | **1** | **-** | **-** | **1** | **-** | **-** |
| **S113N** | **2** | **-** | **-** | **-** | **2** | **-** | **-** | **-** |
| **T113N** | **2** | **-** | **-** | **2** | **-** | **-** | **-** | **-** |
| **V106G** | **2** | **-** | **-** | **2** | **-** | **-** | **-** | **-** |
| **P142L** | **2** | **-** | **-** | **2** | **-** | **-** | **-** | **-** |
| **P142S** | **2** | **-** | **-** | **1** | **-** | **1** | **-** | **-** |
| **P151L** | **2** | **1** | **-** | **-** | **1** | **-** | **-** | **-** |
| **T140A** | **2** | **-** | **-** | **-** | **2** | **-** | **-** | **-** |
| **S117K** | **2** | **2** | **-** | **-** | **-** | **-** | **-** | **-** |
| **T143S** | **2** | **1** | **1** | **-** | **-** | **-** | **-** | **-** |
| **S117T** | **2** | **-** | **-** | **2** | **-** | **-** | **-** | **-** |
| **C137*** | **2** | **2** | **-** | **-** | **-** | **-** | **-** | **-** |
| **D99A** | **2** | **-** | **-** | **2** | **-** | **-** | **-** | **-** |
| **S155P** | **2** | **-** | **-** | **1** | **-** | **1** | **-** | **-** |
| **W156*** | **2** | **1** | **-** | **-** | **-** | **-** | **1** | **-** |
| **T114A** | **2** | **2** | **-** | **-** | **-** | **-** | **-** | **-** |
| **P120Q** | **2** | **-** | **-** | **2** | **-** | **-** | **-** | **-** |
| **G145*** | **2** | **-** | **-** | **1** | **-** | **-** | **1** | **-** |
| **K122_T123delinsN** | **2** | **-** | **-** | **2** | **-** | **-** | **-** | **-** |
| **C121*** | **1** | **-** | **-** | **-** | **-** | **-** | **1** | **-** |
| **T127A** | **1** | **-** | **-** | **-** | **1** | **-** | **-** | **-** |
| **G119_P120delinsG** | **1** | **-** | **1** | **-** | **-** | **-** | **-** | **-** |
| **R160N** | **1** | **-** | **-** | **1** | **-** | **-** | **-** | **-** |
| **K122S** | **1** | **-** | **-** | **1** | **-** | **-** | **-** | **-** |
| **K122I** | **1** | **-** | **1** | **-** | **-** | **-** | **-** | **-** |
| **K122M** | **1** | **-** | **-** | **1** | **-** | **-** | **-** | **-** |
| **C121Y** | **1** | **-** | **-** | **-** | **-** | **1** | **-** | **-** |
| **C121I** | **1** | **-** | **-** | **1** | **-** | **-** | **-** | **-** |
| **C121N** | **1** | **-** | **-** | **1** | **-** | **-** | **-** | **-** |
| **P153Q** | **1** | **1** | **-** | **-** | **-** | **-** | **-** | **-** |
| **P108T** | **1** | **1** | **-** | **-** | **-** | **-** | **-** | **-** |
| **T118P** | **1** | **1** | **-** | **-** | **-** | **-** | **-** | **-** |
| **S114N** | **1** | **-** | **-** | **-** | **-** | **1** | **-** | **-** |
| **P111_T118delinsP** | **1** | **1** | **-** | **-** | **-** | **-** | **-** | **-** |
| **W165*** | **1** | **-** | **-** | **-** | **1** | **-** | **-** | **-** |
| **L127H** | **1** | **-** | **-** | **-** | **-** | **1** | **-** | **-** |
| **P105H** | **1** | **1** | **-** | **-** | **-** | **-** | **-** | **-** |
| **K141R** | **1** | **-** | **-** | **-** | **1** | **-** | **-** | **-** |
| **L162P** | **1** | **1** | **-** | **-** | **-** | **-** | **-** | **-** |
| **L162Q** | **1** | **-** | **-** | **1** | **-** | **-** | **-** | **-** |
| **I150F** | **1** | **1** | **-** | **-** | **-** | **-** | **-** | **-** |
| **M133V** | **1** | **1** | **-** | **-** | **-** | **-** | **-** | **-** |
| **S154*** | **1** | **-** | **-** | **1** | **-** | **-** | **-** | **-** |
| **M133R** | **1** | **-** | **-** | **-** | **1** | **-** | **-** | **-** |
| **K141I** | **1** | **-** | **-** | **1** | **-** | **-** | **-** | **-** |
| **P108L** | **1** | **-** | **-** | **1** | **-** | **-** | **-** | **-** |
| **I110F** | **1** | **1** | **-** | **-** | **-** | **-** | **-** | **-** |
| **K122G** | **1** | **-** | **-** | **1** | **-** | **-** | **-** | **-** |
| **P127I** | **1** | **-** | **1** | **-** | **-** | **-** | **-** | **-** |
| **T115K** | **1** | **-** | **-** | **1** | **-** | **-** | **-** | **-** |
| **C121_K122insRT** | **1** | **1** | **-** | **-** | **-** | **-** | **-** | **-** |
| **C107*** | **1** | **-** | **-** | **1** | **-** | **-** | **-** | **-** |
| **F170Y** | **1** | **-** | **-** | **1** | **-** | **-** | **-** | **-** |
| **L109R** | **1** | **-** | **-** | **-** | **-** | **1** | **-** | **-** |
| **C139S** | **1** | **-** | **-** | **1** | **-** | **-** | **-** | **-** |
| **C139F** | **1** | **-** | **-** | **1** | **-** | **-** | **-** | **-** |
| **G112_S114delinsG** | **1** | **-** | **-** | **1** | **-** | **-** | **-** | **-** |
| **T126N** | **1** | **-** | **1** | **-** | **-** | **-** | **-** | **-** |
| **T116S** | **1** | **-** | **-** | **1** | **-** | **-** | **-** | **-** |
| **T116V** | **1** | **-** | **-** | **-** | **-** | **1** | **-** | **-** |
| **T116I** | **1** | **-** | **-** | **-** | **-** | **-** | **1** | **-** |
| **G112K** | **1** | **1** | **-** | **-** | **-** | **-** | **-** | **-** |
| **G112N** | **1** | **-** | **-** | **-** | **1** | **-** | **-** | **-** |
| **G112Q** | **1** | **-** | **-** | **1** | **-** | **-** | **-** | **-** |
| **G130C** | **1** | **-** | **1** | **-** | **-** | **-** | **-** | **-** |
| **N131H** | **1** | **1** | **-** | **-** | **-** | **-** | **-** | **-** |
| **N131K** | **1** | **1** | **-** | **-** | **-** | **-** | **-** | **-** |
| **T148I** | **1** | **1** | **-** | **-** | **-** | **-** | **-** | **-** |
| **N131S** | **1** | **1** | **-** | **-** | **-** | **-** | **-** | **-** |
| **G112_C121delinsG** | **1** | **-** | **-** | **1** | **-** | **-** | **-** | **-** |
| **N146D** | **1** | **-** | **-** | **-** | **-** | **1** | **-** | **-** |
| **N146S** | **1** | **1** | **-** | **-** | **-** | **-** | **-** | **-** |
| **M103K** | **1** | **-** | **1** | **-** | **-** | **-** | **-** | **-** |
| **S143M** | **1** | **-** | **-** | **-** | **1** | **-** | **-** | **-** |
| **N131I** | **1** | **1** | **-** | **-** | **-** | **-** | **-** | **-** |
| **M103T** | **1** | **-** | **-** | **1** | **-** | **-** | **-** | **-** |
| **S113_T115delinsS** | **1** | **1** | **-** | **-** | **-** | **-** | **-** | **-** |
| **T118L** | **1** | **-** | **-** | **1** | **-** | **-** | **-** | **-** |
| **R169C** | **1** | **-** | **-** | **-** | **-** | **1** | **-** | **-** |
| **K122delinsRTR** | **1** | **1** | **-** | **-** | **-** | **-** | **-** | **-** |
| **A157T** | **1** | **-** | **-** | **-** | **-** | **-** | **1** | **-** |
| **A157D** | **1** | **1** | **-** | **-** | **-** | **-** | **-** | **-** |
| **S114K** | **1** | **-** | **-** | **-** | **-** | **1** | **-** | **-** |
| **S114L** | **1** | **-** | **-** | **1** | **-** | **-** | **-** | **-** |
| **L127S** | **1** | **-** | **-** | **-** | **-** | **1** | **-** | **-** |
| **L127A** | **1** | **-** | **-** | **-** | **-** | **1** | **-** | **-** |
| **F134N** | **1** | **-** | **-** | **1** | **-** | **-** | **-** | **-** |
| **F134Q** | **1** | **-** | **1** | **-** | **-** | **-** | **-** | **-** |
| **L104V** | **1** | **1** | **-** | **-** | **-** | **-** | **-** | **-** |
| **T125N** | **1** | **-** | **-** | **-** | **1** | **-** | **-** | **-** |
| **I110_P111delinsT** | **1** | **-** | **-** | **-** | **1** | **-** | **-** | **-** |
| **C124N** | **1** | **-** | **-** | **1** | **-** | **-** | **-** | **-** |
| **C124F** | **1** | **-** | **-** | **1** | **-** | **-** | **-** | **-** |
| **T131S** | **1** | **-** | **-** | **-** | **1** | **-** | **-** | **-** |
| **C124Y** | **1** | **-** | **-** | **1** | **-** | **-** | **-** | **-** |
| **T113P** | **1** | **-** | **-** | **1** | **-** | **-** | **-** | **-** |
| **T113A** | **1** | **-** | **-** | **1** | **-** | **-** | **-** | **-** |
| **T113K** | **1** | **-** | **-** | **1** | **-** | **-** | **-** | **-** |
| **V106I** | **1** | **-** | **-** | **-** | **-** | **1** | **-** | **-** |
| **P142H** | **1** | **1** | **-** | **-** | **-** | **-** | **-** | **-** |
| **S136*** | **1** | **1** | **-** | **-** | **-** | **-** | **-** | **-** |
| **S113_S114insSTTSAG** | **1** | **-** | **1** | **-** | **-** | **-** | **-** | **-** |
| **T140L** | **1** | **-** | **1** | **-** | **-** | **-** | **-** | **-** |
| **S136A** | **1** | **-** | **1** | **-** | **-** | **-** | **-** | **-** |
| **G159R** | **1** | **-** | **-** | **-** | **1** | **-** | **-** | **-** |
| **G159V** | **1** | **-** | **-** | **-** | **1** | **-** | **-** | **-** |
| **Y134L** | **1** | **-** | **-** | **-** | **1** | **-** | **-** | **-** |
| **Y161H** | **1** | **1** | **-** | **-** | **-** | **-** | **-** | **-** |
| **Y161C** | **1** | **-** | **1** | **-** | **-** | **-** | **-** | **-** |
| **W163R** | **1** | **-** | **-** | **-** | **1** | **-** | **-** | **-** |
| **Y134W** | **1** | **-** | **-** | **-** | **1** | **-** | **-** | **-** |
| **Y134S** | **1** | **-** | **-** | **-** | **1** | **-** | **-** | **-** |
| **S143A** | **1** | **-** | **-** | **1** | **-** | **-** | **-** | **-** |
| **W163*** | **1** | **1** | **-** | **-** | **-** | **-** | **-** | **-** |
| **P120H** | **1** | **-** | **-** | **1** | **-** | **-** | **-** | **-** |
| **S117G** | **1** | **1** | **-** | **-** | **-** | **-** | **-** | **-** |
| **P135A** | **1** | **-** | **-** | **-** | **-** | **1** | **-** | **-** |
| **K122_C124delinsR** | **1** | **-** | **1** | **-** | **-** | **-** | **-** | **-** |
| **T118_C121delinsS** | **1** | **-** | **-** | **1** | **-** | **-** | **-** | **-** |
| **P135R** | **1** | **-** | **-** | **1** | **-** | **-** | **-** | **-** |
| **T113_T115del** | **1** | **-** | **-** | **1** | **-** | **-** | **-** | **-** |
| **F161L** | **1** | **-** | **-** | **1** | **-** | **-** | **-** | **-** |
| **C137S** | **1** | **-** | **-** | **1** | **-** | **-** | **-** | **-** |
| **C137Y** | **1** | **-** | **-** | **1** | **-** | **-** | **-** | **-** |
| **C147S** | **1** | **-** | **-** | **1** | **-** | **-** | **-** | **-** |
| **S117_C121delinsS** | **1** | **-** | **-** | **1** | **-** | **-** | **-** | **-** |
| **S114_T115insTTST** | **1** | **-** | **-** | **1** | **-** | **-** | **-** | **-** |
| **G102V** | **1** | **1** | **-** | **-** | **-** | **-** | **-** | **-** |
| **D99G** | **1** | **-** | **1** | **-** | **-** | **-** | **-** | **-** |
| **D99N** | **1** | **1** | **-** | **-** | **-** | **-** | **-** | **-** |
| **G102N** | **1** | **1** | **-** | **-** | **-** | **-** | **-** | **-** |
| **Q101_P111delinsQ** | **1** | **-** | **-** | **-** | **1** | **-** | **-** | **-** |
| **G112_T114delinsG** | **1** | **1** | **-** | **-** | **-** | **-** | **-** | **-** |
| **S167*** | **1** | **1** | **-** | **-** | **-** | **-** | **-** | **-** |
| **S117_C121delinsN** | **1** | **-** | **-** | **1** | **-** | **-** | **-** | **-** |
| **S155Y** | **1** | **-** | **-** | **1** | **-** | **-** | **-** | **-** |
| **T123V** | **1** | **-** | **1** | **-** | **-** | **-** | **-** | **-** |
| **W156R** | **1** | **-** | **-** | **1** | **-** | **-** | **-** | **-** |
| **G119V** | **1** | **1** | **-** | **-** | **-** | **-** | **-** | **-** |
| **P120I** | **1** | **-** | **-** | **1** | **-** | **-** | **-** | **-** |
| **P111A** | **1** | **-** | **-** | **1** | **-** | **-** | **-** | **-** |
| **Q101P** | **1** | **-** | **-** | **1** | **-** | **-** | **-** | **-** |
| **T114I** | **1** | **1** | **-** | **-** | **-** | **-** | **-** | **-** |
| **Q101N** | **1** | **-** | **-** | **1** | **-** | **-** | **-** | **-** |
| **T114R** | **1** | **1** | **-** | **-** | **-** | **-** | **-** | **-** |
| **G145K** | **1** | **-** | **-** | **1** | **-** | **-** | **-** | **-** |
| **S114_S117delinsC** | **1** | **-** | **-** | **1** | **-** | **-** | **-** | **-** |
| **P111R** | **1** | **-** | **1** | **-** | **-** | **-** | **-** | **-** |
| **P111N** | **1** | **-** | **-** | **1** | **-** | **-** | **-** | **-** |

*Stop codon
