# Supplementary material for: Ultra-deep sequencing reveals high prevalence and broad structural diversity of hepatitis B surface antigen mutations in a global population
Source: PLoS One. 2017 May 4;12(5):e0172101. doi: 10.1371/journal.pone.0172101 (PMC5417417; doi:10.1371/journal.pone.0172101)

**Supplemental Table 8**

Prevalence of 51 MHR “a” determinant region amino acid dimorphisms, which have previously been associated with clinical and diagnostic complications, in four continental populations. Patient numbers are categorized by HBV genotypes (A-G) [13, 19, 25, 47-54].


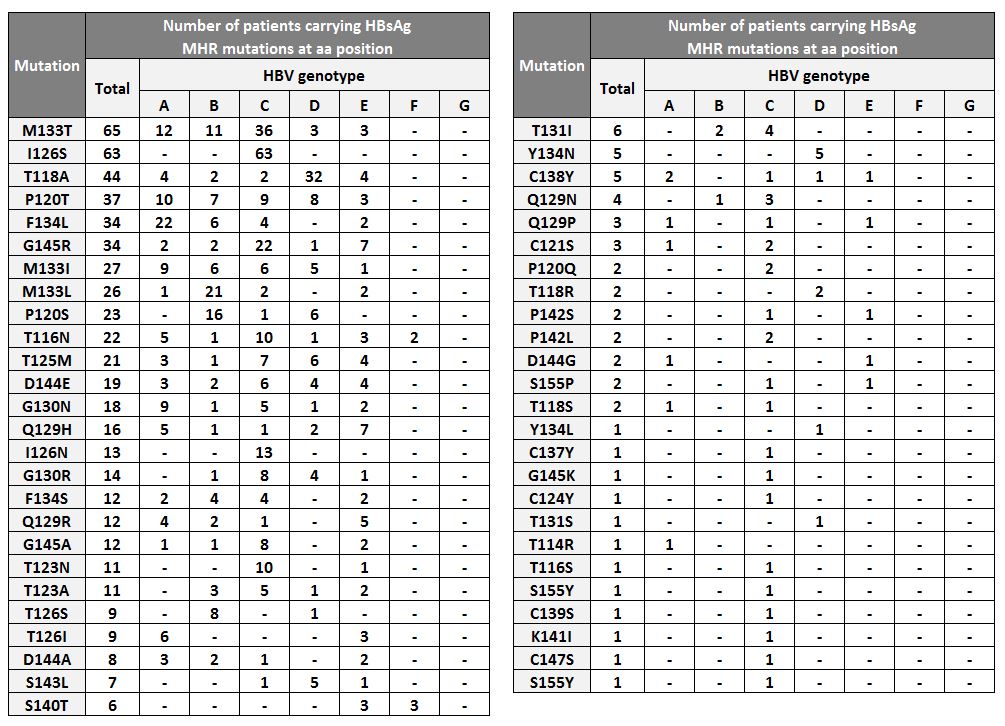

Supplement: S8 Table — Patient numbers are categorized by HBV genotypes (A-G). (DOCX) [file pone.0172101.s010.docx]
