# Supplementary material for: Ultra-deep sequencing reveals high prevalence and broad structural diversity of hepatitis B surface antigen mutations in a global population
Source: PLoS One. 2017 May 4;12(5):e0172101. doi: 10.1371/journal.pone.0172101 (PMC5417417; doi:10.1371/journal.pone.0172101)
Supplement: S9 Table — All 345 MHR mutations detected in this study were subjected to a systematic search in the published literature (PubMed; a subanalysis is summarized in S10 Table), available public databases and the search engine GoogleTM. Retrieved publication records or pertinent information are shown for individual mutations if available. (DOC) [file pone.0172101.s011.doc]

**Supplemental Table 9**

Identification of previously unknown HBsAg MHR mutations. All 345 MHR mutations detected in this study were subjected to a systematic

search in the published literature (PubMed; a subanalysis is summarized in **Supplemental Table 10**), available public databases and the

search engine Google^TM^. Retrieved publication records or pertinent information are shown for individual mutations if available.

| **HBsAg MHR mutation** | **Publication** |
| --- | --- |
|
|
| **D99A** |  |
| **D99G** |  |
| **D99N** | http://www.ncbi.nlm.nih.gov/pmc/articles/PMC4601727/ |
| **Y100C** | http://jvi.asm.org/content/87/14/7882.full.pdf+html |
| **Y100F** | http://www.ncbi.nlm.nih.gov/pmc/articles/PMC3898369/ |
| **Y100S** | http://www.ncbi.nlm.nih.gov/pmc/articles/PMC3898369/ |
| **Y100*** |  |
| **Q101H** | http://www.virologyj.com/content/pdf/1743-422X-9-82.pdf |
| **Q101L** | only polymerase mutation |
| **Q101K** | http://onlinelibrary.wiley.com/doi/10.1053/jhep.2001.28708/epdf |
| **Q101N** |  |
| **Q101P** | http://www.kimbumjoon.pe.kr/webboard/kbjpub/DATA/PDF-19.pdf |
| **Q101_P111delinsQ** | not included in count of novel mutations |
| **Q101R** | http://www.virologyj.com/content/pdf/1743-422X-9-82.pdf |
| **G102A** | only mention so far : A Function Essential to Viral Entry Underlies the Hepatitis B Virus “a” Determinant JOURNAL OF VIROLOGY, Sept. 2009, p. 9321–9328 (Artificial variants) synthetically produced variant  and  [http://wiredspace.wits.ac.za/bitstream/handle/10539/1876/MSc%20Dissertation%20pg%2013-125.pdf?sequence=4](http://wiredspace.wits.ac.za/bitstream/handle/10539/1876/MSc Dissertation pg 13-125.pdf?sequence=4)  source not indicated |
| **G102D** | Nucleic Acid Testing to Detect HBV Infection in Blood Donors N Engl J Med 2011;364:236-47 |
| **G102N** | No publication but mentioned: https://bib.irb.hr/prikazi-rad?&rad=675475 |
| **G102S** | http://onlinelibrary.wiley.com/doi/10.1111/j.1365-2893.2005.00656.x/abstract  No publication but mentioned: https://www.workspace.swe.siemens.com/content/10000003/academie/secmat/2012%20-%20JOURNEE%20HEPATOPATHIES%20CHRONIQUES/Pr%C3%A9sentation%20Dr%20Ly.pdf |
| **G102V** |  |
| **M103K** | http://www.researchgate.net/publication/7902071_Prevalence_of_naturally_occurring_surface_antigen_variants_of_hepatitis_B_virus_in_Korean_patients_infected_chronically |
| **M103I** | http://onlinelibrary.wiley.com/doi/10.1002/jmv.23717/epdf |
| **M103V** | http://journals.plos.org/plosone/article?id=10.1371/journal.pone.0090432 |
| **M103T** | http://www.researchgate.net/publication/7902071_Prevalence_of_naturally_occurring_surface_antigen_variants_of_hepatitis_B_virus_in_Korean_patients_infected_chronically |
| **L104V** |  |
| **L104W** | Genotype, Phylogenetic Analysis, and Transmission Pattern of Occult Hepatitis B Virus (HBV) Infection in Families of Asymptomatic HBsAg Carriers Journal of Medical Virology 78:53–59 (2006) |
| **P105H** | http://www.researchgate.net/publication/24232837_Molecular_characterization_of_occult_hepatitis_B_cases_in_Greek_blood_donors |
| **P105R** | Clinical implications of hepatitis B virus mutations: Recent acvances World J Gastroenterol 2014 June 28; 20(24): 7653-7664 |
| **P105S** |  |
| **V106A** | A Function Essential to Viral Entry Underlies the Hepatitis B Virus “a” Determinant JOURNAL OF VIROLOGY, Sept. 2009, p. 9321–9328, only synthetically generated |
| **V106G** | Chinese paper! Not readable. |
| **V106I** |  |
| **C107Y** | The Prevalence of Surface Antigen Variants of Hepatitis B Virus in Papua New Guinea, South Africa, and Sardinia HEPATOLOGY Vol. 26, No. 6, 1997 |
| **C107*** |  |
| **P108L** |  |
| **P108H** | http://jcm.asm.org/content/44/7/2321.full.pdf |
| **P108S** |  |
| **P108T** | no publication, mentioned in Russion thesis: Скрытая инфекция, вызванная вирусом гепатита В: эпидемиологическая, вирусологическая и клинико-морфологическая характеристика |
| **L109H** |  |
| **L109I** | http://www.intmedpress.com/serveFile.cfm?sUID=e98365b1-dc05-44ad-b43d-1f469c6fe32d |
| **L109M** | Hepatitis B virus infection in a cohort of HIV infected blood donors and AIDS patients in Sichuan, China Liu et al. Journal of Translational Medicine 2014, 12:164 |
| **L109P** | http://onlinelibrary.wiley.com/doi/10.1002/jmv.23717/epdf |
| **L109Q** | http://www.intmedpress.com/serveFile.cfm?sUID=e98365b1-dc05-44ad-b43d-1f469c6fe32d |
| **L109R** | http://www.ncbi.nlm.nih.gov/pubmed/17134939 |
| **L109V** | Lamivudine resistance and other mutations in the polymerase and surface antigen genes of hepatitis B virus associated with a fatal hepatic failure case  Journal of Gastroenterology and Hepatology23 (2008) 67–72 |
| **I110L** | http://www.scielo.br/pdf/bjid/v13n4/v13n4a05.pdf |
| **I110F** | http://applications.emro.who.int/imemrf/Oman_Med_J/Oman_Med_J_2014_29_2_92_96.pdf |
| **I110M** | Sensitivities of Four New Commercial Hepatitis B Virus Surface Antigen (HBsAg) Assays in Detection of HBsAg Mutant Forms JOURNAL OF CLINICAL MICROBIOLOGY, July 2006, p. 2321–2326 |
| **I110N** |  |
| **I110_P111delinsT** | not included in count of novel mutations |
| **I110S** |  |
| **I110V** | The Prevalence of Surface Antigen Variants of Hepatitis B Virus in Papua New Guinea, South Africa, and Sardinia HEPATOLOGY Vol. 26, No. 6, 1997 |
| **L110I** | http://medcraveonline.com/JHVRV/JHVRV-02-00046.pdf |
| **L110P** |  |
| **P111A** |  |
| **P111_G112delinsP** | not included in count of novel mutations |
| **P111L** | http://www.virologyj.com/content/7/1/204 |
| **P111N** |  |
| **P111Q** | A novel immunoassay for PreS1 and/or core-related antigens for detection of HBsAg variants  Journal of Virological Methods168 (2010) 108–113 |
| **P111R** |  |
| **P111_T118delinsP** | not included in count of novel mutations |
| **G112_C121delinsG** | not included in count of novel mutations |
| **G112E** | http://jvi.asm.org/content/87/14/7882.full |
| **G112K** | http://jgv.sgmjournals.org/content/journal/jgv/10.1099/0022-1317-81-5-1165?crawler=true&mimetype=application/pdf |
| **G112N** | only artificially generated: http://jvi.asm.org/content/84/24/12850/F8.expansion.html |
| **G112Q** |  |
| **G112R** | http://onlinelibrary.wiley.com/doi/10.1002/hep.23886/pdf |
| **G112_S114delinsG** | not included in count of novel mutations |
| **G112_T114delinsG** | not included in count of novel mutations |
| **S113A** | http://www.intmedpress.com/serveFile.cfm?sUID=e98365b1-dc05-44ad-b43d-1f469c6fe32d |
| **S113N** | see T113N |
| **S113P** | Hepatitis B genotypes/subgenotypes and MHR variants among Moroccan chronic carriers Journal of Infection (2011) 63, 66-75 |
| **S113T** | http://www.ncbi.nlm.nih.gov/pubmed/25040474 |
| **S113_S114insSTTSAG** | not included in count of novel mutations |
| **S113_T115delinsS** | not included in count of novel mutations |
| **T113P** | see S113P |
| **S113_T114insT** | not included in count of novel mutations |
| **T113A** | see S113A |
| **T113K** |  |
| **T113N** | http://www.kimbumjoon.pe.kr/webboard/kbjpub/DATA/PDF-19.pdf |
| **T113S** | http://www.kimbumjoon.pe.kr/webboard/kbjpub/DATA/PDF-19.pdf |
| **T113_T115del** | not included in count of novel mutations |
| **S114A** | http://onlinelibrary.wiley.com/doi/10.1002/hep.23886/pdf |
| **S114K** |  |
| **S114L** |  |
| **S114N** |  |
| **S114P** | Hepatitis B genotypes/subgenotypes and MHR variants among Moroccan chronic carriers Journal of Infection (2011) 63, 66-75 |
| **S114_S117delinsC** | not included in count of novel mutations |
| **S114_T115insTTST** | not included in count of novel mutations |
| **S114T** | http://www.kimbumjoon.pe.kr/webboard/kbjpub/DATA/PDF-19.pdf |
| **T114A** | http://medcraveonline.com/JHVRV/JHVRV-02-00046.pdf |
| **T114I** |  |
| **T114K** | Published only in Russian: Проблема диагностического ускользания вируса гепатита B |
| **T114P** | http://www.virologyj.com/content/7/1/204 |
| **T114S** | http://onlinelibrary.wiley.com/doi/10.1002/hep.510260640/pdf |
| **T114R** | http://www.producao.usp.br/bitstream/handle/BDPI/10665/art_PINHO_Characterization_of_a_Hepatitis_B_virus_strain_2010.pdf?sequence=1 |
| **T115I** | Anti-hepatitis B core antigen testing with detection and characterization of occult hepatitis B virus by an in-house nucleic acid testing among blood donors in Behrampur, Ganjam, Orissa in southeastern India: implications for transfusion Panigrahi et al. Virology Journal 2010, 7:204 |
| **T115K** |  |
| **T115N** | http://onlinelibrary.wiley.com/doi/10.1002/hep.23886/pdf |
| **T115P** | http://www.spandidos-publications.com/ijo/42/4/1459 |
| **T116A** | http://www.intmedpress.com/serveFile.cfm?sUID=e98365b1-dc05-44ad-b43d-1f469c6fe32d |
| **T116I** | http://en.cnki.com.cn/Article_en/CJFDTotal-RDYZ200711010.htm |
| **T116N** | http://ac.els-cdn.com/S0042682215002950/1-s2.0-S0042682215002950-main.pdf?_tid=eaae006a-35ee-11e5-aa7a-00000aab0f26&acdnat=1438173776_cdc07cd742e2cb28d5636d4d51e19f37 |
| **T116S** | http://onlinelibrary.wiley.com/doi/10.1002/hep.510260640/pdf |
| **T116V** |  |
| **S117C** |  |
| **S117G** | http://www.virologyj.com/content/7/1/204 |
| **S117K** | https://www.researchgate.net/publication/221797848_Variable_capacity_of_13_hepatitis_B_virus_surface_antigen_assays_for_the_detection_of_HBsAg_mutants_in_blood_samples |
| **S117N** | A novel immunoassay for PreS1 and/or core-related antigens for detection of HBsAg variants  Journal of Virological Methods168 (2010) 108–113 |
| **S117R** | Unique surface gene variants of hepatitis B virus isolated from patients in the Philippines J Med Virol. 2014 Feb;86(2):209-16 (only abstract) http://www.ncbi.nlm.nih.gov/pubmed/24009186 |
| **S117T** | http://www.ncbi.nlm.nih.gov/pmc/articles/PMC3342217/ |
| **S117_C121delinsN** | not included in count of novel mutations |
| **S117_C121delinsS** | not included in count of novel mutations |
| **T118A** | http://www.ncbi.nlm.nih.gov/pmc/articles/PMC427827/pdf/1075-03.pdf |
| **T118_C121delinsS** | not included in count of novel mutations |
| **T118K** | http://www.intmedpress.com/serveFile.cfm?sUID=e98365b1-dc05-44ad-b43d-1f469c6fe32d |
| **T118L** | http://www.researchgate.net/publication/221774991_Mutations_in_the_S_gene_region_of_hepatitis_B_virus_genotype_D_in_Golestan_Province-Iran |
| **T118M** | http://jid.oxfordjournals.org/content/198/11/1620.full |
| **T118P** | abstract only: http://www.sciencedirect.com/science/article/pii/S0168706906130086 |
| **T118Q** |  |
| **T118R** | http://www.virologyj.com/content/7/1/204 |
| **T118S** | http://onlinelibrary.wiley.com/doi/10.1002/hep.510260640/pdf |
| **T118V** | Tracking the naturally occurring mutations across the full-length genome of hepatitis B virus of genotype D in different phases of chronic e-antigen-negative infection Clin Microbiol Infect 2012; 18: E412–E418 |
| **G119E** | http://jvi.asm.org/content/87/14/7882.full |
| **G119R** | http://www.intmedpress.com/serveFile.cfm?sUID=e98365b1-dc05-44ad-b43d-1f469c6fe32d |
| **G119_P120delinsG** | not included in count of novel mutations |
| **G119V** | only artificially generated: http://www.ncbi.nlm.nih.gov/pmc/articles/PMC3004315/ |
| **P120A** | Impact of Hepatitis B Virus Surface Protein Mutations on the Diagnosis of Occult Hepatitis B Virus Infection HEPATOLOGY, Vol. 52, No. 5, 2010 |
| **P120L** | http://www.intmedpress.com/serveFile.cfm?sUID=e98365b1-dc05-44ad-b43d-1f469c6fe32d |
| **P120H** | http://www.medicalforum.ch/docs/smf/archiv/de/2010/2010-35/2010-35-136.pdf |
| **P120I** |  |
| **P120Q** | http://medcraveonline.com/JHVRV/JHVRV-02-00046.pdf |
| **P120S** | http://ac.els-cdn.com/S0168170207000615/1-s2.0-S0168170207000615-main.pdf?_tid=b146a65e-3519-11e5-8ba2-00000aab0f27&acdnat=1438082197_8b6dee2fd014c2e8d586888ab5c0eb3b |
| **P120T** | http://medcraveonline.com/JHVRV/JHVRV-02-00046.pdf |
| **C121I** | ???? Only abstract http://www.thelancet.com/journals/lancet/article/PIIS0140-6736%2809%2960739-X/fulltext?version=printerFriendly |
| **C121N** |  |
| **C121R** | only cursory mention in https://www.workspace.swe.siemens.com/content/10000003/academie/secmat/2012%20-%20JOURNEE%20HEPATOPATHIES%20CHRONIQUES/Pr%C3%A9sentation%20Dr%20Ly.pdf |
| **C121S** | Impact of Hepatitis B Virus Surface Protein Mutations on the Diagnosis of Occult Hepatitis B Virus Infection HEPATOLOGY, Vol. 52, No. 5, 2010 |
| **C121Y** | http://www.virologyj.com/content/pdf/1743-422X-7-104.pdf |
| **C121*** |  |
| **C121_K122insRT** | not included in count of novel mutations |
| **K122E** | Occult Hepatitis B Virus Infection: Detection and Significance Dig Dis 2010;28:116–125 |
| **K122G** | only artificially generated: http://www.ncbi.nlm.nih.gov/pmc/articles/PMC3318601/ |
| **K122I** | http://www.ncbi.nlm.nih.gov/pmc/articles/PMC3318601/ |
| **K122M** | http://jvi.asm.org/content/86/8/4658.full.pdf |
| **K122N** | Prevalence of Naturally Occurring Surface Gene Variants of Hepatitis B Virus in Nonimmunized Surface Antigen–Negative Chinese Carriers HEPATOLOGY Vol. 34, No. 5, 2001 |
| **K122Q** |  |
| **K122R** | http://medcraveonline.com/JHVRV/JHVRV-02-00046.pdf |
| **K122S** | only artificially generated:: http://www.ncbi.nlm.nih.gov/pmc/articles/PMC3318601/ |
| **K122_T123delinsN** | not included in count of novel mutations |
| **K122_C124delinsR** | not included in count of novel mutations |
| **K122delinsRTR** | not included in count of novel mutations |
| **R122K** | Impact of Hepatitis B Virus Surface Protein Mutations on the Diagnosis of Occult Hepatitis B Virus Infection HEPATOLOGY, Vol. 52, No. 5, 2010 |
| **T123A** | Detection of Highly Prevalent Hepatitis B Virus Coinfection among HIV-Seropositive Persons in Ghana JOURNAL OF CLINICAL MICROBIOLOGY, Sept. 2010, p. 3223–3230 |
| **T123I** | Development of a Highly Sensitive Bioluminescent Enzyme Immunoassay for Hepatitis B Virus Surface Antigen Capable of Detecting Divergent Mutants Clinical and Vaccine Immunology p. 1255–1265, August 2013 Volume 20 Number 8 |
| **T123N** | http://download.springer.com/static/pdf/867/art%253A10.1007%252Fs10096-015-2358-1.pdf?originUrl=http%3A%2F%2Flink.springer.com%2Farticle%2F10.1007%2Fs10096-015-2358-1&token2=exp=1438259491~acl=%2Fstatic%2Fpdf%2F867%2Fart%25253A10.1007%25252Fs10096-015-2358-1.pdf%3ForiginUrl%3Dhttp%253A%252F%252Flink.springer.com%252Farticle%252F10.1007%252Fs10096-015-2358-1*~hmac=8578a7068fd24ee04a141f27de74ead06afa1c5cbcc17b6150e34fcde18c0294 |
| **T123S** | Spontaneous HBsAg loss in Korean patients: relevance of viral genotypes, S gene mutations, and covalently closed circular DNA copy numbers Clinical and Molecular Hepatology 2014;20:251-260 |
| **T123V** | http://www.ncbi.nlm.nih.gov/pmc/articles/PMC3020474/ |
| **C124F** | http://www.researchgate.net/publication/9017094_Hepatitis_B_virus_reactivation_after_fludarabine-based_regimens_for_indolent_non-Hodgkin%27s_lymphomas_High_prevalence_of_acquired_viral_genomic_mutations |
| **C124N** | http://www.ncbi.nlm.nih.gov/pubmed/16789016 |
| **C124S** | http://www.researchgate.net/publication/13750023_New_hepatitis_B_virus_mutant_form_in_a_blood_donor_that_is_undetectable_in_several_hepatitis_B_surface_antigen_screening_assays |
| **C124Y** | http://medcraveonline.com/JHVRV/JHVRV-02-00046.pdf |
| **T125M** | http://www.scielo.br/pdf/bjid/v13n4/v13n4a05.pdf |
| **T125N** | complete text unavailable: https://www.researchgate.net/publication/284913042_Clinical_significance_of_hepatitis_B_surface_antigen_mutants  (World J Hepatol 2015 November 28; 7(27): 2729-2739) |
| **I126N** | http://www.kimbumjoon.pe.kr/webboard/kbjpub/DATA/PDF-19.pdf |
| **I126S** | http://medcraveonline.com/JHVRV/JHVRV-02-00046.pdf |
| **I126T** | http://medcraveonline.com/JHVRV/JHVRV-02-00046.pdf |
| **I126V** | Quantification of Pregenomic RNA and Covalently Closed Circular DNA in Hepatitis B Virus-Related Hepatocellular Carcinoma International Journal of Hepatology Volume 2013, Article ID 849290, 9 pages |
| **T126A** | http://www.intmedpress.com/serveFile.cfm?sUID=e98365b1-dc05-44ad-b43d-1f469c6fe32d |
| **T126I** | http://medcraveonline.com/JHVRV/JHVRV-02-00046.pdf |
| **T126N** | http://onlinelibrary.wiley.com/doi/10.1053/jhep.2001.28708/pdf |
| **T126S** | http://medcraveonline.com/JHVRV/JHVRV-02-00046.pdf |
| **L127A** | see T127A & P127A |
| **L127F** |  |
| **L127H** | see P127H |
| **L127I** | Detection of Highly Prevalent Hepatitis B Virus Coinfection among HIV-Seropositive Persons in Ghana JOURNAL OF CLINICAL MICROBIOLOGY, Sept. 2010, p. 3223–3230 |
| **L127P** | http://www.ncbi.nlm.nih.gov/pubmed/17503077 |
| **L127S** | http://journals.plos.org/plosone/article?id=10.1371/journal.pone.0039027 |
| **L127V** | Hepatitis B virus in Buenos Aires, Argentina: genotypes, virological characteristics and clinical outcomes Clinical Microbiology and Infection, Volume 17 Number 2, February 2011 |
| **P127A** | http://www.medscape.com/viewarticle/543575_3 |
| **P127I** | http://www.ncbi.nlm.nih.gov/pmc/articles/PMC2860763/ |
| **P127L** | http://www.ncbi.nlm.nih.gov/pmc/articles/PMC2860763/ |
| **P127S** | http://www.medscape.com/viewarticle/543575_3 |
| **P127T** | http://www.ncbi.nlm.nih.gov/pmc/articles/PMC3956465/pdf/pone.0091150.pdf |
| **T127A** | http://www.virologyj.com/content/7/1/204 |
| **P127H** | Clinical implications of hepatitis B virus mutations: Recent acvances World J Gastroenterol 2014 June 28; 20(24): 7653-7664 |
| **T127P** | http://www.ncbi.nlm.nih.gov/pmc/articles/PMC3956465/pdf/pone.0091150.pdf |
| **T127S** | Impact of Hepatitis B Virus Surface Protein Mutations on the Diagnosis of Occult Hepatitis B Virus Infection HEPATOLOGY, Vol. 52, No. 5, 2010 |
| **T127L** | http://www.ncbi.nlm.nih.gov/pmc/articles/PMC2860763/ |
| **A128V** | http://ac.els-cdn.com/S0168170207000615/1-s2.0-S0168170207000615-main.pdf?_tid=b146a65e-3519-11e5-8ba2-00000aab0f27&acdnat=1438082197_8b6dee2fd014c2e8d586888ab5c0eb3b |
| **Q129P** | Impact of Hepatitis B Virus Surface Protein Mutations on the Diagnosis of Occult Hepatitis B Virus Infection HEPATOLOGY, Vol. 52, No. 5, 2010 |
| **Q129N** | Prevalence of Naturally Occurring Surface Gene Variants of Hepatitis B Virus in Nonimmunized Surface Antigen–Negative Chinese Carriers HEPATOLOGY Vol. 34, No. 5, 2001 |
| **Q129L** | http://www.ncbi.nlm.nih.gov/pmc/articles/PMC3342217/ |
| **Q129H** | http://medcraveonline.com/JHVRV/JHVRV-02-00046.pdf |
| **Q129R** | http://www.intmedpress.com/serveFile.cfm?sUID=e98365b1-dc05-44ad-b43d-1f469c6fe32d |
| **G130A** | Variantes de escape del virus de la hepatitis B Rev Chilena Infectol 2015; 32 (2): 190-197 |
| **G130C** |  |
| **G130E** | http://www.intmedpress.com/serveFile.cfm?sUID=e98365b1-dc05-44ad-b43d-1f469c6fe32d |
| **G130K** | only abstract: http://www.jstor.org/stable/30085620?seq=1#page_scan_tab_contents |
| **G130N** | http://medcraveonline.com/JHVRV/JHVRV-02-00046.pdf |
| **G130R** | Prevalence of Naturally Occurring Surface Gene Variants of Hepatitis B Virus in Nonimmunized Surface Antigen–Negative Chinese Carriers HEPATOLOGY Vol. 34, No. 5, 2001 |
| **G130S** | Hepatitis B genotypes/subgenotypes and MHR variants among Moroccan chronic carriers Journal of Infection (2011) 63, 66-75 |
| **N131H** |  |
| **N131I** | see T131I |
| **N131K** | http://onlinelibrary.wiley.com/doi/10.1053/jhep.2002.32710/pdf |
| **N131S** | http://www.researchgate.net/publication/11477440_Reduced_Antigenicity_of_the_Hepatitis_B_Virus_HBsAg_Protein_Arising_as_a_Consequence_of_Sequence_Changes_in_the_Overlapping_Polymerase_Gene_That_Are_Selected_by_Lamivudine_Therapy |
| **N131T** | http://www.hindawi.com/journals/tswj/2013/571875/ |
| **T131I** | http://onlinelibrary.wiley.com/doi/10.1002/hep.510260640/pdf |
| **T131N** | http://medcraveonline.com/JHVRV/JHVRV-02-00046.pdf |
| **T131P** | http://www.biomedcentral.com/1756-0500/5/22 |
| **T131S** | see N131S |
| **S132C** | http://www.ncbi.nlm.nih.gov/pmc/articles/PMC4475248/ |
| **S132F** | Sensitivities of Four New Commercial Hepatitis B Virus Surface Antigen (HBsAg) Assays in Detection of HBsAg Mutant Forms JOURNAL OF CLINICAL MICROBIOLOGY, July 2006, p. 2321–2326 |
| **S132Y** | Long-Term Follow-up of Children With Postnatal Immunoprophylaxis Failure Who Were Infected With Hepatitis B Virus Surface Antigen Gene Mutant Journal of Infectious Diseases Advance Access published January 28, 2013 |
| **M133I** | http://www.ncbi.nlm.nih.gov/pmc/articles/PMC2998485/pdf/1743-422X-7-326.pdf |
| **M133L** | http://medcraveonline.com/JHVRV/JHVRV-02-00046.pdf |
| **M133Q** | http://www.ncbi.nlm.nih.gov/pmc/articles/PMC3004315/ |
| **M133R** |  |
| **M133S** | Hepatitis B Virus DNA Splicing in Lebanese Blood Donors and Genotype A to E Strains: Implications for Hepatitis B Virus DNA Quantification and Infectivity Journal of Clinical Microbiology p. 3159 –316, October 2012 Volume 50 Number 1 |
| **M133T** | http://medcraveonline.com/JHVRV/JHVRV-02-00046.pdf |
| **M133V** | only abstract: http://wprim.whocc.org.cn/local/detail.jsp?channelid=75002&searchword=WPRIMID%3D639356 |
| **F134A** | https://www.researchgate.net/publication/278742539_Performance_of_HBsAg_point-of-care_tests_for_detection_of_diagnostic_escape-variants_in_clinical_samples |
| **(F134C)** | not mentioned so far but Y134C: http://www.wjgnet.com/1007-9327/full/v20/i24/7653-T1.htm |
| **F134H** | ADVIA Centaur® Infectious Disease testing Dr Elise Roper, Siemens Healthcare Diagnostics Siemens Labnews Spring 2011 | Australia and New Zealand |
| **F134I** | http://onlinelibrary.wiley.com/doi/10.1002/jmv.23717/epdf |
| **(F134K)** | not mentioned so far only Chinese paper in Chinese characters (not readable): Clinical significance of combined detecting hepatitis B virus preS1 antigen and core antigen  see also Y134K: http://www.ncbi.nlm.nih.gov/pmc/articles/PMC4698480/ |
| **F134L** | http://www.intmedpress.com/servefile.cfm?suid=b50cd973-e009-41d8-aa50-3d266c8df543 |
| **F134N** | http://edoc.rki.de/oa/articles/rei3JGBxtwUic/PDF/20WFkMdpoCSLQ.pdf |
| **F134Q** |  |
| **F134R** | http://www.ncbi.nlm.nih.gov/pmc/articles/PMC3342217/ |
| **F134S** | http://www.kimbumjoon.pe.kr/webboard/kbjpub/DATA/PDF-19.pdf |
| **F134T** | Y134T http://www.ncbi.nlm.nih.gov/pmc/articles/PMC4510766/ |
| **F134V** | http://www.intmedpress.com/serveFile.cfm?sUID=e98365b1-dc05-44ad-b43d-1f469c6fe32d |
| **F134Y** | http://jgv.sgmjournals.org/content/journal/jgv/10.1099/0022-1317-81-5-1165?crawler=true&mimetype=application/pdf |
| **Y134F** | Hepatitis B Virus S Mutants in Liver Transplant Recipients Who Were Reinfected Despite Hepatitis B Immune Globulin Prophylaxis HEPATOLOGY January 1998 GHANY ET AL 213-222 |
| **Y134H** | Hepatitis B surface antigen genetic elements critical for immune escape correlate with hepatitis B virus reactivation upon immunosuppression Hepatology. 2015 Mar;61(3):823-33 (only abstract) http://www.ncbi.nlm.nih.gov/pubmed/25418031 |
| **Y134L** | http://www.intmedpress.com/serveFile.cfm?sUID=e98365b1-dc05-44ad-b43d-1f469c6fe32d |
| **Y134N** | Molecular and Functional Analysis of OccultHepatitis B Virus Isolates from Patients withHepatocellular Carcinoma HEPATOLOGY, Vol. 45, No. 2, 2007 |
| **Y134S** | http://www.intmedpress.com/serveFile.cfm?sUID=e98365b1-dc05-44ad-b43d-1f469c6fe32d |
| **Y134W** | http://jgv.sgmjournals.org/content/journal/jgv/10.1099/0022-1317-81-5-1165?crawler=true&mimetype=application/pdf |
| **P135A** | http://jms.fudan.edu.cn/EN/abstract/abstract857.shtml |
| **P135H** | Hepatitis B genotypes/subgenotypes and MHR variants among Moroccan chronic carriers Journal of Infection (2011) 63, 66-75 |
| **P135L** | http://edoc.rki.de/oa/articles/rei3JGBxtwUic/PDF/20WFkMdpoCSLQ.pdf |
| **P135R** | http://jgv.sgmjournals.org/content/journal/jgv/10.1099/0022-1317-81-5-1165?crawler=true&mimetype=application/pdf |
| **S136A** |  |
| **S136L** | Unique surface gene variants of hepatitis B virus isolated from patients in the Philippines J Med Virol. 2014 Feb;86(2):209-16 (only abstract) http://www.ncbi.nlm.nih.gov/pubmed/24009186 |
| **S136F** | https://hal.archives-ouvertes.fr/hal-00613776/document |
| **S136Y** | http://www.intmedpress.com/serveFile.cfm?sUID=e98365b1-dc05-44ad-b43d-1f469c6fe32d |
| **S136*** |  |
| **C137S** | http://www.ncbi.nlm.nih.gov/pmc/articles/PMC2169099/ |
| **C137Y** | http://www.researchgate.net/publication/24232837_Molecular_characterization_of_occult_hepatitis_B_cases_in_Greek_blood_donors |
| **C137*** |  |
| **C138Y** | Impaired Virion Secretion by Hepatitis B Virus Immune Escape Mutants and Its Rescue by Wild-Type Envelope Proteins or a SecondSite Mutation Journal of Virology p. 2352–2357, February 2013 Volume 87 Number 4 |
| **C139F** |  |
| **C139S** | http://medcraveonline.com/JHVRV/JHVRV-02-00046.pdf |
| **C139Y** | http://medcraveonline.com/JHVRV/JHVRV-02-00046.pdf |
| **S140L** | http://www.researchgate.net/publication/51422984_Envelope_protein_variability_among_HBV-Infected_asymptomatic_carriers_and_immunized_children_with_breakthrough_infections |
| **S140T** | http://medcraveonline.com/JHVRV/JHVRV-02-00046.pdf |
| **T140A** | http://www.wjgnet.com/1007-9327/full/v21/i6/1794-T2.htm |
| **T140I** | http://www.intmedpress.com/servefile.cfm?suid=b50cd973-e009-41d8-aa50-3d266c8df543 |
| **T140L** | see S140L |
| **T140S** | http://jgv.sgmjournals.org/content/journal/jgv/10.1099/0022-1317-82-2-367?crawler=true&mimetype=application/pdf |
| **K141I** | http://www.researchgate.net/publication/11477440_Reduced_Antigenicity_of_the_Hepatitis_B_Virus_HBsAg_Protein_Arising_as_a_Consequence_of_Sequence_Changes_in_the_Overlapping_Polymerase_Gene_That_Are_Selected_by_Lamivudine_Therapy |
| **K141R** | http://www.ncbi.nlm.nih.gov/pmc/articles/PMC2886939/ |
| **P142L** | http://www.medscape.com/viewarticle/560954_4 |
| **P142H** | only mentioned in Hungarian dissertation and Chinese note (Huashan Hospital)  http://phd.lib.uni-corvinus.hu/404/1/szomor_katalin.pdf  and  http://www.google.de/url?sa=t&rct=j&q=&esrc=s&source=web&cd=2&cad=rja&uact=8&ved=0ahUKEwjGsOKS5szMAhXC2ywKHf45Cl4QFggjMAE&url=http%3A%2F%2Ffile.yynet.cn%3A8080%2Faa41913d66e7de8f871e13a98a27de96%2Fnews_20097802036.doc&usg=AFQjCNG-TDAgrVp0WIcuTlpJBagGp-i9Ug |
| **P142S** | http://www.ncbi.nlm.nih.gov/pubmed/11264736 |
| **S143A** | T143A http://dx.doi.org/10.4236/wjcd.2013.38080 |
| **S143M** | http://jcm.asm.org/content/44/7/2321.full.pdf |
| **S143T** | http://www.ncbi.nlm.nih.gov/pmc/articles/PMC3466907/ |
| **S143L** | http://medcraveonline.com/JHVRV/JHVRV-02-00046.pdf |
| **T143M** | http://www.ncbi.nlm.nih.gov/pubmed/20231988 |
| **T143S** | http://jgv.sgmjournals.org/content/journal/jgv/10.1099/0022-1317-81-5-1165?crawler=true&mimetype=application/pdf |
| **D144A** | http://medcraveonline.com/JHVRV/JHVRV-02-00046.pdf |
| **D144E** | http://jvi.asm.org/content/87/14/7882.full.pdf+html |
| **D144G** | http://medcraveonline.com/JHVRV/JHVRV-02-00046.pdf |
| **G145A** | http://medcraveonline.com/JHVRV/JHVRV-02-00046.pdf |
| **G145E** | Long-term surveillance of haematopoietic stem cell recipients with resolved hepatitis B: high risk of viral reactivation even in a recipient with a vaccinated donor J Viral Hepat. 2007 Jul;14(7):478-83 (only abstract) |
| **G145K** | http://www.ncbi.nlm.nih.gov/pubmed/19571620 |
| **G145R** | <http://medcraveonline.com/JHVRV/JHVRV-02-00046.pdf> |
| **G145V** |  |
| **G145*** |  |
| **N146D** | http://www.ncbi.nlm.nih.gov/pmc/articles/PMC2893152/ |
| **N146S** | http://www.ncbi.nlm.nih.gov/pubmed/16789016 |
| **N146T** | Modification of the Hepatitis B Virus Envelope Protein Glycosylation Pattern Interferes with Secretion of Viral Particles, Infectivity, and Susceptibility to Neutralizing Antibodies Journal of Virology p. 9049 –9059 August 2014 Volume 88 Number 16 |
| **C147S** | not mentioned so far, only artificial variant:  http://jvi.asm.org/content/83/18/9321.full |
| **C147Y** | Impact of Hepatitis B Virus Surface Protein Mutations on the Diagnosis of Occult Hepatitis B Virus Infection HEPATOLOGY, Vol. 52, No. 5, 2010 |
| **T148I** | http://onlinelibrary.wiley.com/doi/10.1002/hep.510300511/pdf |
| **C149Y** | http://www.ncbi.nlm.nih.gov/pubmed/23180290 |
| **I150F** |  |
| **I150T** | <http://www.intmedpress.com/serveFile.cfm?sUID=e98365b1-dc05-44ad-b43d-1f469c6fe32d> |
| **P151L** | http://www.ncbi.nlm.nih.gov/pmc/articles/PMC3020474/ |
| **P153L** | Published Oct. 2015: http://jjmicrobiol.com/?page=article&article_id=23686 |
| **P153Q** | http://www.blackwellpublishing.com/eccmid18/abstract.asp?id=70385 |
| **S154L** | http://www.fcrm-congo.com/website/images/2015/jul/pdf1-2015_obi_plosone.pdf |
| **S154P** | http://jvi.asm.org/content/87/14/7882.full |
| **S154*** |  |
| **S155P** | http://onlinelibrary.wiley.com/doi/10.1002/hep.510260640/pdf |
| **S155Y** | http://onlinelibrary.wiley.com/doi/10.1002/hep.510260640/pdf |
| **W156L** | http://www.intmedpress.com/servefile.cfm?suid=b50cd973-e009-41d8-aa50-3d266c8df543 |
| **W156R** | http://www.researchgate.net/publication/229436112_Subgenotype_D5_BCP_and_MHR_mutations_in_hepatic_complications_among_hepatitis_B_virus_infected_patients_from_Orissa_India |
| **W156*** | http://jcm.asm.org/content/early/2015/07/16/JCM.00602-15.full.pdf |
| **A157D** | http://onlinelibrary.wiley.com/doi/10.1002/hep.510270144/pdf |
| **A157G** | http://onlinelibrary.wiley.com/doi/10.1002/hep.21529/full |
| **A157T** | http://www.researchgate.net/publication/5850269_Frequency_and_significance_of_hepatitis_B_virus_surface_gene_variant_circulating_among_antiHBc_only_individuals_in_Eastern_India |
| **A157V** | Prevalence of Naturally Occurring Surface Antigen Variants of Hepatitis B Virus in Korean Patients Infected Chronically Journal of Medical Virology 76:194–202 (2005) |
| **F158L** | https://www.google.de/url?sa=t&rct=j&q=&esrc=s&source=web&cd=1&cad=rja&uact=8&ved=0CCEQFjAAahUKEwigr42v-KPHAhWH7RQKHeiyDFc&url=http%3A%2F%2Fwww.scirp.org%2Fjournal%2FPaperDownload.aspx%3FpaperID%3D39695&ei=D3HLVeBrh9tT6OWyuAU&usg=AFQjCNE6w_GhKh8sAck_1yB---IvL73dCA&bvm=bv.99804247,d.d24 |
| **F158S** | A serological and molecular survey of hepatitis B in children 15 years after inception of the national hepatitis B vaccination program in eastern China. J Med Virol. 2009 Sep;81(9):1517-24 (only abstract) |
| **A159G** | http://dx.doi.org/10.4236/wjcd.2013.38080 |
| **A159V** | http://www.plosone.org/article/fetchObject.action?uri=info:doi/10.1371/journal.pone.0099028&representation=PDF |
| **G159A** | http://www.scielo.br/pdf/bjid/v13n4/v13n4a05.pdf |
| **G159E** | Lamivudine Resistance Mutations in European Patients With Hepatitis B and Patients Co-Infected With HIV and Hepatitis B Journal of Medical Virology 9999:1–4 (2011) |
| **G159R** | http://www.ncbi.nlm.nih.gov/pmc/articles/PMC4252145/ |
| **G159V** | http://www.ncbi.nlm.nih.gov/pubmed/25500663 |
| **K160N** | Biological significance of amino acid substitutions in hepatitis B surface antigen (HBsAg) for glycosylation, secretion, antigenicity and immunogenicity of HBsAg and hepatitis B virus replication Journal of General Virology (2010), 91, 483–492 |
| **K160R** | http://www.ncbi.nlm.nih.gov/pubmed/15834887 |
| **R160K** | http://www.ncbi.nlm.nih.gov/pmc/articles/PMC3834618/pdf/TSWJ2013-212704.pdf |
| **R160N** | http://www.researchgate.net/publication/6583177_Molecular_analysis_of_hepatitis_B_virus_a_determinant_in_asymptomatic_and_symptomatic_Mexican_carriers |
| **F161L** | http://www.ncbi.nlm.nih.gov/pmc/articles/PMC514758/ |
| **F161Y** | Hepatitis B surface antigen variants in voluntary blood donors in Nanjing, China Yong-lin et al. Virology Journal 2012, 9:82 |
| **Y161C** | http://www.ncbi.nlm.nih.gov/pubmed/25732900 |
| **Y161F** | http://www.scielo.br/pdf/bjid/v13n4/v13n4a05.pdf |
| **Y161H** | F161H https://art.torvergata.it/retrieve/handle/2108/19504/29667/Role%20of%20hepatitis%20B%20virus%20genetic%20barrier%20in%20drug-resistance%20and%20immune-escape%20development.pdf |
| **Y161S** | http://medcraveonline.com/JHVRV/JHVRV-02-00046.pdf |
| **L162P** | only found by Google search. In abstract no indication of mutation, full text not available http://www.sciencedirect.com/science/article/pii/S0168706906130086 |
| **L162Q** | http://jcm.asm.org/content/38/7/2793.full.pdf |
| **W163R** | http://onlinelibrary.wiley.com/doi/10.1002/jmv.20503/pdf |
| **W163*** | http://www.ncbi.nlm.nih.gov/pmc/articles/PMC4572547/ |
| **E164A** | Molecular and Functional Analysis of OccultHepatitis B Virus Isolates from Patients withHepatocellular Carcinoma HEPATOLOGY, Vol. 45, No. 2, 2007 |
| **E164D** | http://medcraveonline.com/JHVRV/JHVRV-02-00046.pdf |
| **E164G** | http://www.virologyj.com/content/pdf/1743-422X-9-82.pdf |
| **E164V** | http://www.intmedpress.com/serveFile.cfm?sUID=e98365b1-dc05-44ad-b43d-1f469c6fe32d |
| **W165L** | http://www.virologyj.com/content/pdf/1743-422x-9-82.pdf |
| **W165S** | http://www.researchgate.net/publication/24232837_Molecular_characterization_of_occult_hepatitis_B_cases_in_Greek_blood_donors |
| **W165*** | http://www.ncbi.nlm.nih.gov/pmc/articles/PMC4572547/ |
| **A166G** | http://www.ncbi.nlm.nih.gov/pmc/articles/PMC4019103/ |
| **A166V** | http://onlinelibrary.wiley.com/doi/10.1053/he.2000.6407/pdf |
| **S167L** | Specific Amino Acid Substitutions in the S Protein Prevent Its Excretion In Vitro and May Contribute to Occult Hepatitis B Virus Infection Journal of Virology p. 7882–7892 July 2013 Volume 87 Number 14 |
| **S167*** |  |
| **V168A** | http://www.ncbi.nlm.nih.gov/pmc/articles/PMC3460816/pdf/pone.0046345.pdf |
| **A168V** | http://onlinelibrary.wiley.com/doi/10.1002/jmv.20503/pdf |
| **R169C** |  |
| **R169H** | http://www.intmedpress.com/serveFile.cfm?sUID=93a79deb-b36b-4203-a642-5863e7f83561  and  (only artificial) http://www.ncbi.nlm.nih.gov/pmc/articles/PMC3004315/  and  dissertation: http://eprints.ucm.es/17360/1/T34071.pdf |
| **R169P** | http://jvi.asm.org/content/78/7/3262.full |
| **F170S** | http://www.researchgate.net/publication/250926918_Immunological_profiling_of_Hepatitis_B_virus_Surface_gene_in_Pakistan |
| **F170Y** |  |

*Stop codon
