# Supplementary material for: Ultra-deep sequencing reveals high prevalence and broad structural diversity of hepatitis B surface antigen mutations in a global population
Source: PLoS One. 2017 May 4;12(5):e0172101. doi: 10.1371/journal.pone.0172101 (PMC5417417; doi:10.1371/journal.pone.0172101)
Supplement: S10 Table — Mutations that are present in three different continents are highlighted (blue). *Stop codon. (DOC) [file pone.0172101.s012.doc]

**Supplemental Table 10**

Origin of individual patients bearing newly identified 62 HBsAg MHR mutations. Mutations that are present in three different continents are highlighted (blue).

| **Novel HBsAg MHR mutation** | **Number of patients carrying novel HBsAg MHR mutation** | **Country of origin of each individual patient** | | | | | | | | |
| --- | --- | --- | --- | --- | --- | --- | --- | --- | --- | --- |
| **D99A** | **2** | **South Korea** | **Vietnam** |  |  |  |  |  |  |  |
| **D99G** | **1** | **Vietnam** |  |  |  |  |  |  |  |  |
| **Y100*** | **2** | **Sudan** | **Venezuela** |  |  |  |  |  |  |  |
| **Q101L** | **5** | **Senegal** | **South Africa** | **South Korea** | **USA** | **USA** |  |  |  |  |
| **Q101N** | **1** | **USA** |  |  |  |  |  |  |  |  |
| **G102A** | **4** | **South Korea** | **South Africa** | **South Africa** | **USA** |  |  |  |  |  |
| **G102N** | **1** | **South Africa** |  |  |  |  |  |  |  |  |
| **G102V** | **1** | **South Africa** |  |  |  |  |  |  |  |  |
| **L104V** | **1** | **Germany** |  |  |  |  |  |  |  |  |
| **P105S** | **2** | **Guinea-Bissau** | **Guinea-Bissau** |  |  |  |  |  |  |  |
| **V106A** | **5** | **Nicaragua** | **Philippines** | **Philippines** | **USA** | **USA** |  |  |  |  |
| **V106I** | **1** | **Guinea-Bissau** |  |  |  |  |  |  |  |  |
| **C107*** | **1** | **USA** |  |  |  |  |  |  |  |  |
| **P108L** | **1** | **South Korea** |  |  |  |  |  |  |  |  |
| **P108S** | **2** | **South Africa** | **South Africa** |  |  |  |  |  |  |  |
| **P108T** | **1** | **Cameroon** |  |  |  |  |  |  |  |  |
| **L109H** | **2** | **South Africa** | **USA** |  |  |  |  |  |  |  |
| **I110N** | **2** | **Germany** | **Germany** |  |  |  |  |  |  |  |
| **I110S** | **4** | **South Africa** | **Saudi Arabia** | **Saudi Arabia** | **USA** |  |  |  |  |  |
| **L110P** | **3** | **South Korea** | **South Korea** | **South Korea** |  |  |  |  |  |  |
| **P111A** | **1** | **South Korea** |  |  |  |  |  |  |  |  |
| **P111N** | **1** | **Vietnam** |  |  |  |  |  |  |  |  |
| **P111R** | **1** | **USA** |  |  |  |  |  |  |  |  |
| **G112N** | **1** | **Saudi Arabia** |  |  |  |  |  |  |  |  |
| **G112Q** | **1** | **Vietnam** |  |  |  |  |  |  |  |  |
| **T113K** | **1** | **South Korea** |  |  |  |  |  |  |  |  |
| **S114K** | **1** | **Guinea-Bissau** |  |  |  |  |  |  |  |  |
| **S114L** | **1** | **USA** |  |  |  |  |  |  |  |  |
| **S114N** | **1** | **Guinea-Bissau** |  |  |  |  |  |  |  |  |
| **T114I** | **1** | **South Africa** |  |  |  |  |  |  |  |  |
| **T115K** | **1** | **South Korea** |  |  |  |  |  |  |  |  |
| **T116V** | **1** | **Sudan** |  |  |  |  |  |  |  |  |
| **S117C** | **2** | **Germany** | **South Korea** |  |  |  |  |  |  |  |
| **T118Q** | **2** | **South Korea** | **South Korea** |  |  |  |  |  |  |  |
| **G119V** | **1** | **Philippines** |  |  |  |  |  |  |  |  |
| **P120I** | **1** | **South Korea** |  |  |  |  |  |  |  |  |
| **C121N** | **1** | **USA** |  |  |  |  |  |  |  |  |
| **C121R** | **2** | **South Korea** | **USA** |  |  |  |  |  |  |  |
| **C121*** | **1** | **Venezuela** |  |  |  |  |  |  |  |  |
| **K122G** | **1** | **South Korea** |  |  |  |  |  |  |  |  |
| **K122Q** | **6** | **South Africa** | **South Korea** | **South Korea** | **South Korea** | **USA** | **USA** |  |  |  |
| **K122S** | **1** | **South Korea** |  |  |  |  |  |  |  |  |
| **T125N** | **1** | **France** |  |  |  |  |  |  |  |  |
| **L127F** | **2** | **Venezuela** | **Venezuela** |  |  |  |  |  |  |  |
| **G130C** | **1** | **USA** |  |  |  |  |  |  |  |  |
| **N131H** | **1** | **South Africa** |  |  |  |  |  |  |  |  |
| **M133R** | **1** | **Saudi Arabia** |  |  |  |  |  |  |  |  |
| **F134Q** | **1** | **Vietnam** |  |  |  |  |  |  |  |  |
| **S136A** | **1** | **USA** |  |  |  |  |  |  |  |  |
| **S136*** | **1** | **South Africa** |  |  |  |  |  |  |  |  |
| **C137*** | **2** | **South Africa** | **USA** |  |  |  |  |  |  |  |
| **C139F** | **1** | **South Korea** |  |  |  |  |  |  |  |  |
| **P142H** | **1** | **South Africa** |  |  |  |  |  |  |  |  |
| **G145V** | **5** | **South Korea** | **Sudan** | **Sudan** | **USA** | **USA** |  |  |  |  |
| **G145*** | **2** | **Nicaragua** | **South Korea** |  |  |  |  |  |  |  |
| **C147S** | **1** | **South Korea** |  |  |  |  |  |  |  |  |
| **I150F** | **1** | **Cameroon** |  |  |  |  |  |  |  |  |
| **S154*** | **1** | **USA** |  |  |  |  |  |  |  |  |
| **L162P** | **1** | **South Africa** |  |  |  |  |  |  |  |  |
| **S167*** | **1** | **South Africa** |  |  |  |  |  |  |  |  |
| **R169C** | **1** | **Guinea-Bissau** |  |  |  |  |  |  |  |  |
| **F170Y** | **1** | **South Korea** |  |  |  |  |  |  |  |  |

*Stop codon
