# Supplementary material for: Ultra-deep sequencing reveals high prevalence and broad structural diversity of hepatitis B surface antigen mutations in a global population
Source: PLoS One. 2017 May 4;12(5):e0172101. doi: 10.1371/journal.pone.0172101 (PMC5417417; doi:10.1371/journal.pone.0172101)
Supplement: S11 Table — The three cohorts contributing most to the pool of novel variants are highlighted (blue). Note that individual percentages do not add up to 100% because various countries share variants. (DOC) [file pone.0172101.s013.doc]

**Supplemental Table 11**

Geographic distribution of 62 novel HBsAg MHR mutations (in descending order according to novel variants per cohort). The three cohorts contributing most to the pool of novel variants are highlighted (blue). Note that individual percentages do not add up to 100% because various countries share variants.

| **Country** | **Cohort size** | **Total number of novel HBsAg MHR mutations per cohort** | **Relative contribution (%) to the pool of novel mutations (n=62)** |
| --- | --- | --- | --- |
| **South Korea** | **233** | **25** | **40.3** |
| **USA** | **200** | **21** | **33.9** |
| **South Africa** | **197** | **17** | **27.4** |
| **Guinea-Bissau** | **106** | **6** | **9.7** |
| **Vietnam** | **258** | **5** | **8.1** |
| **Saudi Arabia** | **80** | **4** | **6.5** |
| **Germany** | **68** | **4** | **6.5** |
| **Sudan** | **51** | **4** | **6.5** |
| **Venezuela** | **6** | **4** | **6.5** |
| **Philippines** | **66** | **3** | **4.8** |
| **Nicaragua** | **21** | **2** | **3.2** |
| **Cameroon** | **17** | **2** | **3.2** |
| **France** | **45** | **1** | **1.6** |
| **Senegal** | **5** | **1** | **1.6** |
| **Argentina** | **5** | **-** | **-** |
| **Dem. Republic of Congo** | **2** | **-** | **-** |
| **Ivory Coast** | **18** | **-** | **-** |
| **Peru** | **3** | **-** | **-** |
| **Spain** | **1** | **-** | **-** |
| **Thailand** | **8** | **-** | **-** |
