# Supplementary material for: Ultra-deep sequencing reveals high prevalence and broad structural diversity of hepatitis B surface antigen mutations in a global population
Source: PLoS One. 2017 May 4;12(5):e0172101. doi: 10.1371/journal.pone.0172101 (PMC5417417; doi:10.1371/journal.pone.0172101)
Supplement: S12 Table — (DOCX) [file pone.0172101.s014.docx]

**Supplemental Table 12**

**Synopsis of HBsAg MHR genotyping studies published 1995-2016 (n=54, MHR variant frequencies in descending order)**

| **Study** | | **Year** | **Cohort ethnicity** | | **Continent** | **Number of sequenced patients^1^** | | **Sequencing technique** | **Fraction of patients carrying MHR mutations (%)** |
| --- | --- | --- | --- | --- | --- | --- | --- | --- | --- |
| Riuz-Tachiquin ME et al. [55] | | 2007 | Mexico | | America | 21 | | Sanger | 95.0 |
| Yong-Lin et al. [25] | | 2012 | China | | Asia | 39 | | Sanger | 51.3 |
| Zhang et al. [56] | | 2015 | China | | Asia | 81 | | Sanger | 49.4 |
| Shi et al. [57] | | 2012 | China | | Asia | 161 | | Sanger | 46.6 |
| Song et al. [14] | | 2005 | South Korea | | Asia | 101 | | Sanger | 46.5 |
| Sayan et al. [26] | | 2012 | Turkey | | Europe | 94 | | Sanger | 46.0 |
| Srey et al. [58] | | 2006 | Cambodia | | Asia | 22 | | Sanger | 45.4 |
| Hou et al. [44] | | 2001 | China | | Asia | 46 | | Sanger | 43.0 |
| Yu et al. [59] | | 2014 | China | | Asia | 216 | | Sanger | 43.0 |
| Kwange et al. [60] | | 2013 | Kenya | | Africa | 21 | | Sanger | 42.9 |
| Mantovani et al. [61] | | 2013 | Brazil | | America | 34 | | Sanger | 41.2 |
| Avellon et al. [49] | | 2006 | Spain | | Europe | 272 | | Sanger | 39.0 |
| Oon et al. [62] | | 1995 | Singapore | | Asia | 41 | | Sanger | 39.0 |
| Neumann-Fraune et al. [28] | | 2013 | Germany | | Europe | 60 | | Sanger | 36.7 |
| Kim et al. [27] | | 2013 | South Korea | | Asia | 41 | | Sanger | 36.6 |
| Pourkarim et al. [63] | | 2014 | Iran | | Asia | 358 | | Sanger | 32.8 |
| Xia et al. [91] | | 2001 | China | | Asia | 97 | | Sanger | 30.9 |
| Al-Qudari et al. [29] | | 2016 | Saudi Arabia | | Africa/Asia | 20 | | Sanger | 30.0 |
| Davaalkham et al. [22] | | 2007 | Mongolia | | Asia | 57 | | Sanger | 29.8 |
| Huang et al. [64] | | 2012 | China | | Asia | 207 | | Sanger | 29.5 |
| Baclig et al. [65] | | 2014 | Philippines | | Asia | 53 | | Sanger | 28.3 |
| Hsu et al. [24] | | 2004 | Taiwan | | Asia | 32 | | Sanger | 28.1 |
| Kim et al. [41] | | 2014 | South Korea | | Asia | 25 | | Sanger | 28.0 |
| Roque-Afonso et al. [67] | | 2007 | France | | Europe | 180 | | Sanger | 27.8 |
| Lee et al. [68] | | 1997 | Taiwan | | Asia | 22 | | Sanger | 27.3 |
| Sayiner et al. [69] | | 2008 | Turkey | | Asia | 81 | | Sanger | 27.2 |
| Mohebbi et al. [70] | | 2012 | Iran | | Asia | 81 | | Sanger | 27.1 |
| Fylaktou et al. [71] | | 2011 | Greece | | Europe | 135 | | Sanger | 26.0 |
| Thuy et al. [72] | | 2005 | Vietnam | | Asia | 40 | | Sanger | 25.0 |
| Wakil et al. [73] | | 2002 | India | | Asia | 26 | | Sanger | 23.0 |
| Lazarevic et al. [74] | | 2010 | Serbia | | Europe | 164 | | Sanger | 22.6 |
| Hsu et al. [24] | | 2010 | Taiwan | | Asia | 31 | | Sanger | 22.6 |
| Theamboonlers et al. [75] | | 2001 | Thailand | | Asia | 49 | | Sanger | 22.4 |
| Chiou et al. [47] | | 1997 | China | | Asia | ND | | Sanger | 20.0 |
| Sa-Nguanmoo et al. [76] | | 2010 | Cambodia, Laos, Myanmar, Thailand | | Asia | 224 | | Sanger | 18.0 |
| Wang et al. [77] | | 2015 | China | | Asia | 278 | | Sanger | 17.6 |
| Liu et al. [78] | | 2012 | China | | Asia | 228 | | Sanger | 17.1 |
| Kitab et al. [79] | | 2011 | Morocco | | Africa | 134 | | Sanger | 15.0 |
| Bian et al. [53] | | 2013 | China | | Asia | 101 | | Sanger | 14.8 |
| Piñeiro et al. [23] | | 2008 | Argentina | | America | 88 | | Sanger | 14.8 |
| Hundie et al. [80] | | 2016 | Ethiopia | | Africa | 51 | | Sanger | 14.0 |
| Dong et al. [81] | | 2009 | China | | Asia | 51 | | Sanger | 14.0 |
| Al Baqlani et al. [82] | | 2014 | Oman | | Africa/Asia | 170 | | Sanger | 12.9 |
| Mallory et al. [13] | | 2011 | USA | | America | 946 | | Sanger | 11.0 |
| Hudu et al. [83] | | 2015 | Malaysia | | Asia | 55 | | Sanger | 10.9 |
| Guptan et al. [84] | | 1996 | India | | Asia | 120 | | Sanger | 10.8 |
| Meldal et al. [85] | | 2011 | Malaysia | | Asia | 77 | | Sanger | 9.0 |
| Sayan et al. [86] | | 2010 | Turkey | | Europe | 142 | | Sanger | 8.3 |
| Forbi et al. [87] | | 2013 | Ivory Coast, Ghana, Cameroon, Uganda | | Africa | 143 | | Sanger | 7.7 |
| Servant-Delmas et al. [30] | | 2010 | France | | Europe | 940 | | Sanger | 5.5 |
| Davies et al. [88] | | 2013 | Australia^2^ | | Australia | 49 | | Sanger | 4.0 |
| Kazim et al. [89] | | 2006 | India | | Asia | 57 | | Sanger | 3.5 |
| Sticchi et al. [38] | | 2013 | Italy | | Europe | 256 | | Sanger | 3.1 |
| Suppiah et al. [90] | | 2014 | Malaysia | | Asia | 93 | | Sanger | 2.2 |
| **Total number of patients** | | | | | | **7111** | |  | **26.4** |
| **Mean cohort size** | | | | | | **134** | |  | **Mean mutation frequency** |
| ^1^minimum cohort size ≥20 patients; ^2^indigenous Australians | | | | | | | | | |
|  |  | | |  | | |  |  |  |
